# Supplementary material for: An engaged research study to assess the effect of a ‘real-world’ dietary intervention on urinary bisphenol A (BPA) levels in teenagers
Source: BMJ Open. 2018 Feb 3;8(2):e018742. doi: 10.1136/bmjopen-2017-018742 (PMC5829847; doi:10.1136/bmjopen-2017-018742)
Supplement: Supplementary file 1 [file bmjopen-2017-018742supp001.pdf]

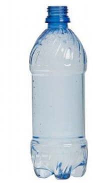

BPA: Myth and Reality
Dietary Intervention Guidelines

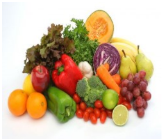

General instructions

The purpose of this dietary intervention trial is to follow a diet designed to minimise routes of exposure to the food packaging chemical bisphenol A (BPA). For the dietary intervention period, please follow as closely as possible the instruction given below. Try to maintain your diet during the intervention period to be as closely similar to your normal diet as possible, in terms of the content, amount and calorific value of the food you eat. Please record details of each meal and the drinks and snacks you consume on the forms provided. Below are some general cooking and eating tips and an indication of which foods are best to avoid and those that are considered a low source of BPA.

Cooking and eating tips for the intervention period.

The general approach is to replace any food items that fall into the 'avoidance' category with an alternative, chosen to minimise exposure to BPA

- **Switch to stainless steel and glass food storage and drink containers.**
- **Move foods to ceramic or glass food containers before microwaving.**
- **Consider a coffee filter or percolator for coffee – home coffee makers (Such as Nespresso™) may have polycarbonate-based water tanks and phthalate-based tubing.**
- **Eat out less, especially at restaurants that do not use fresh ingredients.**
- **Avoid canned food consumption. Where possible, replace with fresh produce or cardboard or tetrapack packaged alternatives.**
- **Choose fresh fruits and vegetables when possible, and frozen if not.**
- **Soak dried beans for cooking rather than tinned.**

Foods to avoid

Tinned foods. Top ten tinned foods that are reported to be sources of BPA include coconut milk, soup, meat, vegetables, meals (e.g. pasta with sauce), juice, fish, beans, meal replacement drinks, fruit.

Carbonated/fizzy drinks and juices in cans. Avoid carbonated drinks in cans and drinks stored for prolonged periods in reusable sports bottles, unless they are labelled ‘BPA free’ (many commercial sports bottles are).

Fast food from commercial outlets. Most processed food has passed through numerous processes, and each additional processing step provides an opportunity for BPA to enter through packaging or tubing. Try to replace fast and processed foods with a freshly prepared and cooked alternative.

Packaged fruit and vegetables. Replace these where possible with unpackaged, loose fruit and vegetable items as far as possible.

Convenience/ready meals. Plastics types considered safest in terms of chemical migration are recycling numbers 2 and 5. Avoid food prepared in packaging with recycling number 7, which includes many different types of polymer and mixed polymers, including polycarbonate, a source of BPA. Try to avoid foods that are designed to be heated in the microwave in their packaging.

Chocolate and ice cream. Individuals who report eating chocolate bars and ice cream on a regular basis have been reported to have higher than average BPA exposure. Try to avoid excessive consumption.

Non-food or food packaging routes of exposure

Although plastics found in consumer goods such as DVDs, CDs, computer goods and sunglasses do contain BPA, this is not an important route of exposure.

Till receipts often contain high levels of BPA, so wash your hands before eating or drinking if you have been handling them.

Dental sealants may contain BPA, so avoid any pre-planned dental work

Example daily diet

Food Item	Comments
**Breakfast**	
Cereal, Fruit	
Milk	Polypropylene or glass packaging
Bread	
Yoghurt	Choose polypropylene container
**Lunch**	
Meat or fish products	Check packaging and avoid those labelled no. 7.<br>Avoid tinned ingredients
Cheese	
Salad items, Fruit	Choose unpackaged where possible, wash before use
Pasta	
**Dinner**	
Shepherds pie	Cooked in saucepan and oven rather than microwaved in plastic
Green beans	Fresh or frozen
Bread	
**Drinks**	
Water	Water direct from tap or use stainless steel or BPA free water bottle
Tea/coffee	Prepare in teapot or cafetiere, avoid commercial coffee makers
Carbonated drinks	Avoid canned drinks and those stored in reusable containers for prolonged periods
Milk	Polypropylene or glass packaging
**Snacks**	
Fruit	
Potato crisps	
