# Supplementary material for: An engaged research study to assess the effect of a ‘real-world’ dietary intervention on urinary bisphenol A (BPA) levels in teenagers
Source: BMJ Open. 2018 Feb 3;8(2):e018742. doi: 10.1136/bmjopen-2017-018742 (PMC5829847; doi:10.1136/bmjopen-2017-018742)
Supplement: Supplementary file 2 [file bmjopen-2017-018742supp002.pdf]

Place participant barcode here	FOOD - DAY 1		DRINK – DAY 1	
**Contents of meal**	Was food packaged in plastic known or suspected to contain BPA?	Was food cooked in plastic known or suspected to contain BPA?	**Type of Drink**	Packaging (please describe) e.g. canned, plastic (Δ number), tetrapak
**Breakfast:**				
1.				
2.				
3.				
4.				
5.				
**Lunch**:				
1.				
2.				
3.				
4.				
5.				
**Dinner**:				
1.				
2.				
3.				
4.				
5.				
**Snacks**:				
1.				
2.				
3.				
4.				

Place participant barcode here	FOOD - DAY 2		DRINK – DAY 2	
**Contents of meal**	Was food packaged in plastic known or suspected to contain BPA?	Was food cooked in plastic known or suspected to contain BPA?	**Type of Drink**	Packaging (please describe) e.g. canned, plastic (Δ number), tetrapak
**Breakfast:**				
1.				
2.				
3.				
4.				
5.				
**Lunch**:				
1.				
2.				
3.				
4.				
5.				
**Dinner**:				
1.				
2.				
3.				
4.				
5.				
**Snacks**:				
1.				
2.				
3.				
4.				

Place participant barcode here	FOOD - DAY 3		DRINK – DAY 3	
**Contents of meal**	Was food packaged in plastic known or suspected to contain BPA?	Was food cooked in plastic known or suspected to contain BPA?	**Type of Drink**	Packaging (please describe) e.g. canned, plastic (∆ number), tetrapak
**Breakfast:**				
1.				
2.				
3.				
4.				
5.				
**Lunch**:				
1.				
2.				
3.				
4.				
5.				
**Dinner**:				
1.				
2.				
3.				
4.				
5.				
**Snacks**:				
1.				
2.				
3.				
4.				

Place participant barcode here	FOOD - DAY 4		DRINK – DAY 4	
**Contents of meal**	Was food packaged in plastic known or suspected to contain BPA?	Was food cooked in plastic known or suspected to contain BPA?	**Type of Drink**	Packaging (please describe) e.g. canned, plastic (Δ number), tetrapak
**Breakfast:**				
1.				
2.				
3.				
4.				
5.				
**Lunch**:				
1.				
2.				
3.				
4.				
5.				
**Dinner**:				
1.				
2.				
3.				
4.				
5.				
**Snacks**:				
1.				
2.				
3.				
4.				

Place participant barcode here	FOOD - DAY 5		DRINK – DAY 5	
**Contents of meal**	Was food packaged in plastic known or suspected to contain BPA?	Was food cooked in plastic known or suspected to contain BPA?	**Type of Drink**	Packaging (please describe) e.g. canned, plastic (Δ number), tetrapak
**Breakfast:**				
1.				
2.				
3.				
4.				
5.				
**Lunch**:				
1.				
2.				
3.				
4.				
5.				
**Dinner**:				
1.				
2.				
3.				
4.				
5.				
**Snacks**:				
1.				
2.				
3.				
4.				

Place participant barcode here	FOOD - DAY 6		DRINK – DAY 6	
**Contents of meal**	Was food packaged in plastic known or suspected to contain BPA?	Was food cooked in plastic known or suspected to contain BPA?	**Type of Drink**	Packaging (please describe) e.g. canned, plastic (Δ number), tetrapak
**Breakfast:**				
1.				
2.				
3.				
4.				
5.				
**Lunch**:				
1.				
2.				
3.				
4.				
5.				
**Dinner**:				
1.				
2.				
3.				
4.				
5.				
**Snacks**:				
1.				
2.				
3.				
4.				

Place participant barcode here	FOOD - DAY 7		DRINK – DAY 7	
**Contents of meal**	Was food packaged in plastic known or suspected to contain BPA?	Was food cooked in plastic known or suspected to contain BPA?	**Type of Drink**	Packaging (please describe) e.g. canned, plastic (Δ number), tetrapak
**Breakfast:**				
1.				
2.				
3.				
4.				
5.				
**Lunch**:				
1.				
2.				
3.				
4.				
5.				
**Dinner**:				
1.				
2.				
3.				
4.				
5.				
**Snacks**:				
1.				
2.				
3.				
4.				

Participant barcode

Additional study information.

Please do not feel obliged to answer these questions if you are uncomfortable doing so.

Gender

☐ Female
☐ Male
☐ Prefer not to say

Tobacco Usage – Have you used tobacco over the past week

☐ Yes
If so, what type and how much?____
☐ No
☐ Prefer not to say

Alcohol Usage – Have you used alcohol over the past week

☐ Yes
If so, what type and how much?____
☐ No
☐ Prefer not to say

Medication- Have you taken any medication over the last week?

☐ Yes
☐ No
☐ Prefer not to say

If so, Please name the medication____________________ ☐ Prefer not to say

Vegetarian/vegan diet - Have you eaten or drank any soya products over the past week?

☐ Yes
☐ No
☐ Prefer not to say

Your measurements - leave blank if you prefer not to say

Your height __________________________

Your weight__________________________
