# Supplementary material for: An engaged research study to assess the effect of a ‘real-world’ dietary intervention on urinary bisphenol A (BPA) levels in teenagers
Source: BMJ Open. 2018 Feb 3;8(2):e018742. doi: 10.1136/bmjopen-2017-018742 (PMC5829847; doi:10.1136/bmjopen-2017-018742)
Supplement: Supplementary file 3 [file bmjopen-2017-018742supp003.pdf]

<u>Supplementary information file 3. Example daily diet diary</u>. A score of 1 is given to each item containing suspected to contain BPA or be packaged in BPA-containing materials. Highly processed foods are also scored as 1, due to uncertainties in the processing procedures. The daily totals are summed to produce a BPA risk score for the 7 day intervention.

	**Item**	**Packaging**	**Score**
**Breakfast**			
	Homemade pancakes	None	0
	Sugar	None	0
	lemon	None	0
	milk	HDPE	0
**Lunch**			
	Homemade Cheese sandwich	none	0
	Homemade sultana cake	none	0
	water	glass	0
**Dinner**			
	Homemade omelette	none	0
	Sweetcorn	Can	1
	Rice	Cellophane	0
	Tomatoes	none	0
	water	glass	0
**Snacks**			
	Crisps (processed)	Cellophane	1
	Apple	none	0
	milk	HDPE	0
		**Total for day**	**2**
