# Supplementary material for: An engaged research study to assess the effect of a ‘real-world’ dietary intervention on urinary bisphenol A (BPA) levels in teenagers
Source: BMJ Open. 2018 Feb 3;8(2):e018742. doi: 10.1136/bmjopen-2017-018742 (PMC5829847; doi:10.1136/bmjopen-2017-018742)
Supplement: Supplementary file 4 [file bmjopen-2017-018742supp004.pdf]

Participant Barcode

BPA: Myth and Reality diet questionnaire

1. Were there any times during the week that you knowingly/unknowingly did not stick to the diet? Please tick any that apply and give indication of frequency.

School meals ☐ ____times

Restaurants/cafés ☐ ____times

Friends' houses ☐ ____times

Takeaway ☐ ____times

Other ____________________________ ☐ ____times

2. If you heated your food in a microwave, what was the food in? Tick any which apply and give indication of frequency.

A food storage container or bowl known or suspected to contain BPA ☐_____times

3. When you or your family drank water, where did your water come from? Tick any which apply and give indication of frequency.

Plastic filter jug known or suspected to contain BPA ☐_____times

Individual water bottle known or suspected to contain BPA ☐_____times

Larger water container known or suspected to contain BPA ☐_____times

4. How many times during the week did you eat food that had been stored or transported in plastic containers known or suspected to contain BPA?

____

5. How many times during the week did you eat tinned food or drink from cans?

____

6. Did the BPA reduced diet affect How much you spent on shopping?

Spent more ☐

Spent less ☐

No difference ☐

7. Did it take longer to source your food than usual?

Yes ☐ No ☐

If so, why? ____

____

8. Did it take longer to prepare food than usual?

Yes ☐ No ☐

If so, why? ____

____

9. How restricted did you feel by your food choice?

☐ Very ☐ Slightly ☐ No difference

If you felt you were restricted by the diet, why was this? ______________________

____

10. Did the diet affect your calorific intake?

Yes ☐ No ☐

If so, why? ____

____

11. How easy would you find it to sustain this diet over a longer period of time?

Very easy ☐

Easy ☐

Hard ☐

Very hard ☐

Not sure ☐

12. Is there anything else about following the diet that you would like to add?
