# Supplementary material for: An engaged research study to assess the effect of a ‘real-world’ dietary intervention on urinary bisphenol A (BPA) levels in teenagers
Source: BMJ Open. 2018 Feb 3;8(2):e018742. doi: 10.1136/bmjopen-2017-018742 (PMC5829847; doi:10.1136/bmjopen-2017-018742)
Supplement: Supplementary file 5 [file bmjopen-2017-018742supp005.pdf]

Thank you for reading this leaflet. If you wish to participate in this study, you will be asked to agree to the consent statements below in the presence of a member of the research team.

CONSENT STATEMENTS

1. I confirm that I have read this information sheet and have discussed participation in this project with my family. I have had opportunity to consider the information, ask questions and have had these questions answered satisfactorily. *You should not give consent until you are happy that you understand what the study involves.*

2. I understand that my participation is voluntary and that I am free to withdraw at any time without giving any reason, without my right to participate in the rest of the study being affected. *This means that even if you helped design this study you do not have to be a participant and you should not feel under any pressure to participate.*

3. I agree to participate in this study as a research subject. *This means that you agree to participate in a one-week diet and to provide two blood and urine samples.*

4. I understand that my anonymised blood and urine samples and linked anonymous questionnaire data will be sent to University of Exeter Medical School, Royal Devon & Exeter Hospital and my urine sample only will be sent to the Rolvaltain laboratory, a specialist BPA analysis company. *This means that laboratory staff will not know that samples belong to you, but dedicated staff at the University of Exeter, with training and experience in data protection ,will be able to link your sample data to your questionnaire data.*

5. I understand that RNA (genetic material) will be extracted from my blood and will be stored anonymously. *This means that Professor Harries' team will use our RNA to provide data that you will help analyse but may also do further research on the samples to identify reasons for any changes seen.*

6. I understand that data relating to my participation in the study will be returned anonymously to my school to be used for educational purposes. *This means that although you will get to analyse data from your samples there is no way you will know which data relates to your samples and which to other participants*.

Complaints:

If you have any complaints about the way in which this study has been carried out please contact the Chair of the University of Exeter Medical School Research Ethics Committee Peta Foxall PhD, Chair, UEMS Research Ethics Committee: P.J.D.Foxall@exeter.ac.uk.

This project has been reviewed and approved by the University of Exeter Medical School Research Ethics committee UEMS REC REFERENCE NUMBER: 15/07/074)

BPA: Myth or Reality?

A research study investigating the effect of chemicals in plastic on gene activity and whether dietary interventions can reduce BPA levels in teenagers.

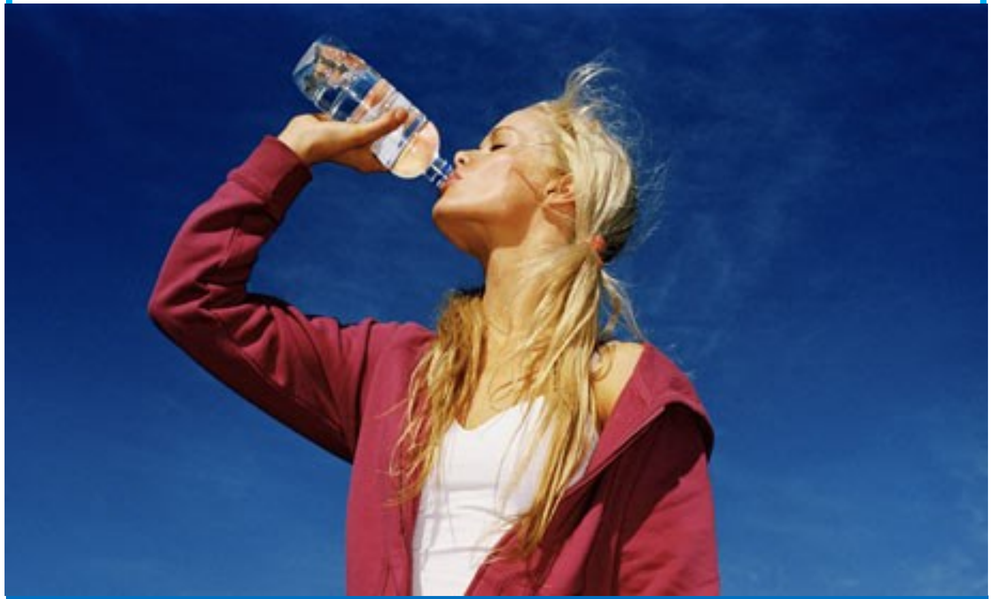

Involvement & Engagement

The aim of this year-long project is to involve teenagers in a research study that is relevant to them, by allowing them to help design a research project, analyse non-identifiable participant data and help to present and publish the outcomes.

Participation

Students will be asked to undertake a one week diet to reduce their intake of BPA, a chemical found in plastics. They will be asked to provide urine and blood samples before and after their diet.

What is BPA?

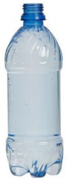

BPA (Bisphenol A) is a chemical used in the manufacture of plastics. Plastics containing BPA are found in a wide range of products including food and drink containers. BPA in these products can be ingested and there are concerns that high BPA levels in the blood could possibly affect human health. Research is therefore needed to understand its effects on the human body and how we can reduce its consumption by minor changes to our diet.

This project is being run as a student-involvement project to answer two specific questions:

1. **Can we see the effects of dietary BPA on our genes?**
2. **Can we effectively reduce BPA in our diet?**

In the past, small-scale experiments have shown that BPA levels in the human body can be reduced by rigid dietary interventions but these interventions would be difficult to implement in the "real world". In this study a one-week dietary intervention designed by teenagers will be used by them to determine whether BPA levels, and the activity of BPA-responsive genes can be effectively reduced in young people by avoiding food packaging that contains this chemical.

What will I need to do?

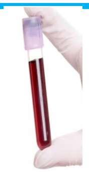

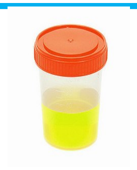

M

Day 1

- Provide a nurse with a 2.5ml blood sample and a urine sample.

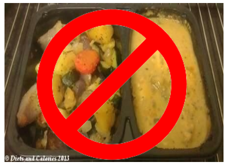

T

W

T

Day 2 - Day 8

- Follow a diet that you have helped to design .
- The diet will exclude sources of BPA as much as possible but will be nutritionally and calorifically similar to your usual diet.
- You will be asked to complete a food diary and answer a questionnaire about how easy it was to follow this diet.

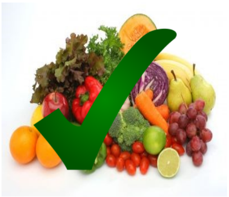

F

S

S

Day 8

- Provide a nurse with a 2.5ml blood sample and a urine sample.

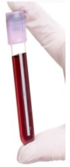

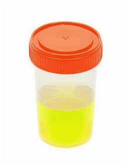

M

We recommend that you discuss the project with your family and involve them in planning what you eat and how you will prepare it.

What will happen to my samples and data?

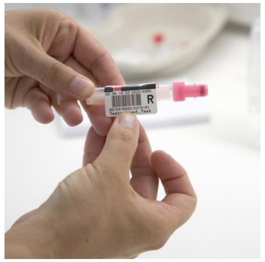

When you participate in the study you will be allocated with a numerical study ID. Your samples and data will be labelled with this number so that we can match your 'before' and 'after' diet samples with your food diary data. Once all data has been collated and coded it will be further anonymised by a person external to the project so that no data can be linked to any of the participants.

Urine samples from before and after the diet will be sent to the Royal Devon & Exeter NHS Foundation Trust for creatinine analysis and to the Rolvaltain laboratory for BPA analysis. RNA will be extracted from blood samples at the Royal Devon & Exeter Molecular Genetics Laboratory and the expression levels of two BPA-responsive genes will be measured in the samples taken before and after the diet. These anonymised RNA samples will be stored and used only by Professor Harries team for further research on the mechanisms behind our findings.

What are the benefits of taking part?

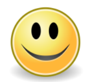

This project will help you to understand how you might be able to reduce BPA in your diet and your involvement in the design will give you an excellent insight into clinical research, community outreach and scientific practise. Your role as a participant is unlikely to have any direct health benefits.

Are there any risks in taking part?

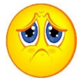

Blood samples will be taken by fully qualified and insured NHS personnel. Any potential discomfort or side-effects will be equivalent to that experienced giving a blood sample to your GP. All data will be fully anonymised before analysis. This means that you will not find out anything about your blood or urine samples. Following the diet may minimally increase the cost of your groceries for the week, but since fresh foods are usually less expensive than pre-packaged foods, we do not expect this to be an issue.

What will happen to the results of the research study?

You will be given the opportunity to help analyse anonymised data from this project and to help disseminate the outcomes of this research. It is hoped that the findings will be published in peer-reviewed journals and the wider media.

Who is organising this research?

The research is organised by Professors Lorna Harries & Tamara Galloway of the University of Exeter as part of their research program into BPA and part of the University's outreach program to involve schools in academic research.
