# Supplementary material for: An engaged research study to assess the effect of a ‘real-world’ dietary intervention on urinary bisphenol A (BPA) levels in teenagers
Source: BMJ Open. 2018 Feb 3;8(2):e018742. doi: 10.1136/bmjopen-2017-018742 (PMC5829847; doi:10.1136/bmjopen-2017-018742)
Supplement: Supplementary file 6 [file bmjopen-2017-018742supp006.pdf]

Version 4 (2/8/2015)

BPA: Myth & Reality

STUDY ID

CONSENT STATEMENTS	Please circle
1. I confirm that I have read information sheet BPA PIS Version 4 and have discussed participation in this project with my family. I have had opportunity to consider the information, ask questions and have had these questions answered satisfactorily. *You should not give consent until you are happy that you understand what the study involves.*	**YES / NO**
2. I understand that my participation is voluntary and that I am free to withdraw at any time without giving any reason, without right to participate in the rest of the study being affected. *This means that even if you helped design this study you do not have to be a participant and you should not feel under any pressure to participate.*	**YES / NO**
3. I agree to participate in this study as a research subject. *This means that you agree to participate in a one-week diet and to provide two blood and urine samples.*	**YES / NO**
4. I understand that my anonymised blood and urine samples and linked anonymous questionnaire data will be sent to University of Exeter Medical School, Royal Devon & Hospital and my urine sample only will be sent to the Rolvaltain laboratory, a specialist BPA analysis company. *This means that laboratory staff will not know that samples belong to you, but dedicated staff at the University of Exeter, with training and experience in data protection, will be able to link your sample data to your questionnaire data.*	**YES / NO**
5. I understand that RNA (genetic material) will be extracted from my blood and will be stored anonymously. *This means that Professor Harries' team will use our RNA to provide data that you will help analyse but may also do further research on the samples to identify reasons for any changes seen.*	**YES / NO**
6. I understand that data relating to my participation in the study will be returned anonymously to my school to be used for educational purposes. *This means that although you will get to analyse data from your samples there is no way you will know which data relates to your samples and which to other participants*.	**YES / NO**

Name of Participant	Signature	Date
Name of Person Obtaining Consent	Signature	Date

I the above signed testify the participant is providing voluntary and fully informed consent to participate in this study. I am on the delegation log to obtain consent for this study and are trained in obtaining consent.

This project has been reviewed and approved by the University of Exeter Medical School Research Ethics Committee UEMS REC REFERENCE NUMBER: 15/07/074)

Version 4 (2/8/2015)

BPA: Myth & Reality
