# Supplementary material for: An engaged research study to assess the effect of a ‘real-world’ dietary intervention on urinary bisphenol A (BPA) levels in teenagers
Source: BMJ Open. 2018 Feb 3;8(2):e018742. doi: 10.1136/bmjopen-2017-018742 (PMC5829847; doi:10.1136/bmjopen-2017-018742)
Supplement: Author's manuscript [file bmjopen-2017-018742.draft_revisions.pdf]

BMJ Open is committed to open peer review. As part of this commitment we make the peer review history of every article we publish publicly available.

When an article is published we post the peer reviewers' comments and the authors' responses online. We also post the versions of the paper that were used during peer review. These are the versions that the peer review comments apply to.

The versions of the paper that follow are the versions that were submitted during the peer review process. They are not the versions of record or the final published versions. They should not be cited or distributed as the published version of this manuscript.

BMJ Open is an open access journal and the full, final, typeset and author-corrected version of record of the manuscript is available on our site with no access controls, subscription charges or pay-per-view fees (http://bmjopen.bmj.com).

If you have any questions on BMJ Open's open peer review process please email editorial.bmjopen@bmj.com

Can we moderate our own exposure to bisphenol A (BPA) through dietary intervention?

Journal:	*BMJ Open*
Manuscript ID	bmjopen-2017-018742
Article Type:	Research
Date Submitted by the Author:	20-Jul-2017

Complete List of Authors:	Galloway, Tamara; University of Exeter, College of Life and Environmental Sciences<br>Baglin, Nigel; Research Projects<br>Lee, Benjamin; University of Exeter Medical School, Institute of Biomedical and Clinical Studies<br>Kocur, Anna; University of Exeter Medical School, Institute of Biomedical and Clinical Studies<br>BPA Schools, Study Consortium; University of Exeter Medical School, Institute of Biomedical and Clinical Studies<br>Shepherd, Maggie; Royal Devon and Exeter National Health Service Foundation Trust, National Institute for Health Research Exeter Clinical Research Facility<br>Steele, Anna; Royal Devon and Exeter National Health Service Foundation Trust, National Institute for Health Research Exeter Clinical Research Facility<br>Harries, Lorna; University of Exeter Medical School, Institute of Biomedical and Clinical Studies

**Primary Subject Heading**:	Public health
Secondary Subject Heading:	Communication
Keywords:	Bisphenol A, Dietary intervention, PUBLIC HEALTH, community, Engaged research

Can we moderate our own exposure to bisphenol A (BPA) through dietary intervention?

Tamara. S. Galloway[1], Nigel Baglin[2], Benjamin P Lee[3], A.L. Kocur[3], BPA schools study consortium[#], M.H. Shepherd[4], A.M. Steele[4] and L.W. Harries[3]

[1]College of Life and Environmental Sciences, University of Exeter, Exeter UK EX4 4AS.
[2]Research Projects, Exeter, UK, EX2 5DQ.
[3]RNA-Mediated Disease Mechanisms group, Institute of Biomedical and Clinical Sciences, University of Exeter Medical School, University of Exeter, Exeter, UK EX2 5DW.
[4]National Institute for Health Research Exeter Clinical Research Facility, Royal Devon and Exeter National Health Service Foundation Trust, and University of Exeter Medical School, Exeter, U.K.

[#] Consortium members listed in supplementary information file 1

Word count 3021

Correspondence to:

Prof Lorna Harries
University of Exeter Medical School,
Barrack Road,
Exeter, EX2 5DW
Tel =44 1392 406773
Email: L.W.Harries@exeter.ac.uk

Declaration of competing issues

The authors have no competing interests to declare.

For peer review only

ABSTRACT

Objective

Bisphenol A has been associated adverse human health outcomes and exposure to this compound is near-ubiquitous in the Western world. We aimed to examine whether self-moderation of BPA exposure is possible by altering diet in a real-world setting.

Design

An Engaged Research dietary intervention study designed, implemented and analysed by healthy teenagers from 6 schools and undertaken in their own homes.

Participants

104 students aged between 18 and 19 years from schools in the South West of the UK provided diet diaries and urine samples for analysis. Questionnaires and freeform comments on the ease of use were collected for qualitative analyses.

Intervention

Researcher participants designed a set of literature-informed guidelines for reduction of dietary BPA to be followed for 7 days.

Main outcome measure

Creatinine-adjusted urinary BPA levels were taken before and after the intervention. Information on packaging and food/drink ingested was used to calculate a BPA risk score for anticipated exposure. A qualitative analysis was carried out to identify themes addressing long term sustainability of the diet.

Results

For peer review only

BPA was detected in urine of 86% of participants at baseline at a median value of 1.34 ng/ml (IQR 1.82). No effect of the intervention diet on BPA levels was identified overall ($p = 0.25$), but there was a positive association in those participants who showed a drop in urinary BPA concentration post intervention and their initial BPA level ($p = 0.003$). Qualitative analysis identified themes around feelings of lifestyle restriction and the inadequacy of current labelling practices.

Conclusions

We found no evidence in this self-administered intervention study that it was possible to moderate BPA exposure by diet in a real world setting. Furthermore, our study participants indicated that they would be unlikely to sustain such a diet long term, due to the difficulty in identifying BPA-free foods.

For peer review only

Article Summary

Strengths of the study

- This study represents the largest assessment to date of the potential for moderating one's own BPA exposure through diet
- The study was carried out in a real-world setting rather than a regulated, controlled environment.
- The study was carried out in teenagers, the demographic with amongst the highest exposure.
- Qualitative analysis reveals challenges with sustaining such a diet.

Limitations of the study

- Calculation of a risk score is challenging due to the pervasive nature of BPA contamination

For peer review only

What is already known on this topic

- Bisphenol A (BPA) is an endocrine disrupting chemical ubiquitously present in our diet and environment. It has been associated with a variety of adverse human health outcomes in animal models, in human populations and in human in-vitro work.
- Previous studies have shown that it may be possible to reduce BPA exposure by following a researcher-supplied very controlled diet for a period of 3 days.

What this study adds

- Our study suggests that although it may be possible to reduce dietary BPA in a controlled setting, it is not possible to do so in a community-based 'real world' setting.
- Qualitative research suggests that even if it were possible to reduce BPA exposure by dietary means, the restrictions placed on lifestyle and the current inadequacies in labelling of BPA containing foods means that such a diet would be impossible to follow long-term, in the real world.
- Improved labelling of foods and packaging of foodstuffs suspected to contain BPA may allow consumers an informed choice on their own exposure.

INTRODUCTION

Bisphenol A is one of the world's highest production volume chemicals. It is used in the manufacture of polycarbonate and other plastic consumer products, in heat resistant papers, dental sealants and in the epoxy resin-based lining of food and drink containers [1]. BPA can be found above the detection limit in the urine of the majority of people worldwide [2]. Concern has been raised for public health, since BPA is classified as an endocrine disrupting chemical (EDC) which has been linked with several disorders in cell and animal models [3-5]. Epidemiological data in humans is more contentious, due to relatively small sample sizes and issues around causality [6]. The Endocrine Society concluded in 2015 that current evidence suggests that BPA and other endocrine disrupting chemicals may have effects on several reproductive, cardiovascular and metabolic traits in humans [7]. The current opinion of food regulatory bodies such as the European Food Standards Agency (EFSA) is that sufficient uncertainty remains to be able to exclude effects on the reproductive, immune, nervous, metabolic and cardiovascular systems and on cancer development [3] whilst the European Chemicals Agency (ECHA) has recently reclassified BPA as a chemical of very high concern due to its endocrine disrupting properties [8].

There has been wide interest in the sources of BPA and the potential for individuals to reduce their own exposure. Human exposure has been reported from inhalation of dust, uptake across the skin from thermal papers and till receipts and release from dental sealants. The main source is the ingestion of food and drink contaminated with BPA leached from packaging materials [1, 9]. BPA is rapidly metabolised in the gut wall and liver and removed from the blood by the kidneys, with a terminal half-life of 6 hours after oral ingestion [10]. BPA has been detected in food samples packaged in glass, plastic, paper and paperboard

For peer review only

cartons, with an average concentration of 0.46 ng/g, rising to over 700 ng/g for certain canned foods. Conversely, in a dietary intervention study in which 22 volunteers consumed a 3 day fresh food diet which excluded canned or packaged foods, there was a 66% reduction in urinary BPA excretion compared to concentrations before the intervention [11]. This latter study involved full dietary replacement of foodstuffs, an approach which is impractical for the population at large. A follow up study found that households who followed written recommendations produced by health care professionals showed no significant change in their BPA exposure [12].

We present an alternative, citizen-science based approach, where student volunteers designed and undertook their own intervention diet, following provision of educational materials. We questioned whether adherence to a self-designed and self-administered 'real world' diet over 7 days would lead to significant reductions in excreted urinary BPA, and if so, whether such a diet was likely to be sustainable in the long term.

METHODS

Participant group

We chose adolescents because they represent a demographic with high BPA exposure (aggregated exposure of 1.449 μg/kg body weight per day) [3, 13]. 104 students aged 17-19 from 6 local schools signed up to participate in this engaged research project, representing the largest intervention study in the population demographic with the highest BPA exposure to date [13]. Study size was arrived upon based on anticipated effect sizes from previous work of this nature [11], and we allowed for a 10% dropout rate. Students designed all of the

For peer review only

materials required for completion of the study (study protocols, food diaries, lifestyle questionnaires, patient information sheets and consent forms (see Supplementary Information files 2 to 5).

Ethical Permission

Ethical permission was granted by the University of Exeter Medical School Ethics Committee (reference number 15/07/074) and the study was carried out in accordance with the Declaration of Helsinki.

The intervention diet

Students designed a “real world” diet designed to reduce consumption of BPA by avoidance of processed foods and foods packaged in known sources of BPA [1, 9]; supplementary information file 2). We requested that calorific intake was maintained as near to their usual diet as possible and recorded details of their daily diet including all food and drink, and its associated packaging, in a self-reported food diary (Supplementary information file 3). A ‘BPA risk score’ based on instances of known or suspected exposure for each participant was then calculated. Risk scores from the final day of the intervention only were also taken, since this is most relevant to the sample collected at visit 2. Information on lifestyle factors including sex, BMI and time of urine collection was also collected (Supplementary information file 4).

For peer review only

Sample collection and measurement of urinary BPA

Urine samples were collected into BPA-free bottles (Vacutest Kima, Italy) immediately before and after the intervention, and were frozen at -20˚C within 4 hours. Each participant was sampled in the same time-slot at both visits to account for circadian variation in BPA metabolism. Samples were transported on dry ice to a commercial laboratory (Rovaltain Research Company, Aixain, France) where analysis of total BPA was assessed by gas chromatography-tandem mass spectrometry. Experimental methods were validated for linearity, detection limit and accuracy and specificity of quantification based on the Standard NF T 90-201 for determination of xenobiotics. A quality control check of known standards injected every 6 samples at two levels of concentration (0.5 ng/ml and 5 ng/ml) was quantified with each batch of unknown samples. Water-only samples were included as negative controls. Urinary creatinine was measured at the Royal Devon and Exeter Hospital using the Jaffe method on the Roche P800 platform (Roche, Mannheim, Germany), to allow correction for urine dilution. Results were expressed as a BPA:creatinine ratio. Samples where BPA was detected but quantifying at or around the limits of quantification (LoQ) were scored as LoQ/√2 according to the method of Hornung and Reed [14].

Statistical Analysis

Study population demographics for urinary BPA adjusted for creatinine at visits 1 and 2 were assessed to generate a ΔBPA continuous variable. BPA risk scores were calculated as a continuous variable. The relationship between urinary BPA levels before and after the 7 day intervention was assessed using a repeated-measure ANOVA, adjusted for sex, time of sampling and BMI, with and without correction for creatinine. The relationship between urinary BPA at visit 1 and whether or not the participants had lower BPA at visit 2 was also

For peer review only

examined by binary logistic regression, adjusted for sex, time of sampling and BMI. Here, samples showing small changes < 0.5ng/ml in either direction were omitted to avoid natural stoichiometric variation around zero. The relationship between change in BPA (ΔBPA) and BPA risk score was assessed by linear regression, adjusted for sex, time of sampling and BMI both with and without adjustment for creatinine. Statistical analysis was carried out using SPSS, v.22 (IBM, USA).

Impact of following reduced BPA diet on lifestyle

We carried out quantitative and qualitative analysis to address long-term sustainability of the diet. Data on the impact of following the diet on feelings of dietary restriction, time spent sourcing or preparing meals, calorific intake and long term sustainability were collected via a questionnaire (See Supplementary information file 4). The questionnaire also included a freeform section where participants could write about their experiences following the diet in a non-prescribed fashion for qualitative analysis. Qualitative data was assessed for thematic content by two experienced qualitative researchers. Key themes were independently identified and coded until agreement was reached.

RESULTS

Participant Characteristics

There were 104 volunteer participants in this engaged research study. Information on the characteristics of the study cohort are given in table 1.

For peer review only

<u>Table 1: Characteristics of the study population</u>. Data were available on 99 out of 104 participants. IQR = interquartile range, SD = Standard deviation. The units of BPA are ng/ml and BMI is defined as Kg/m^2. LoQ = limit of quantification. Urinary BPA levels are given both as unadjusted data and as a BPA (ng/ml) to creatinine (mg/ml) ratio.

Unadjusted urinary BPA at visit 1 (n = 98)	
median (IQR)	1.37 (2.52)
95% confidence intervals	1.58 to 2.57
mean (SD)	2.07 (2.51)
Number of samples below LoQ (0.1ng/ml)	16
Minimum value (ng/ml)	0.07
Maximum value (ng/ml)	13.55
Creatinine-adjusted urinary BPA at visit 1 (n = 98)	
median (IQR)	1.34 (1.82)
95% confidence intervals	1.38 to 2.13
mean (SD)	1.75 (1.82)
Number of samples below LoQ (0.1ng/ml)	16
Minimum value (ng/ml)	0.05
Maximum value (ng/ml)	9.52
Unadjusted urinary BPA at visit 2 (n = 99)	
median (IQR)	1.91 (2.68)
95% confidence intervals	2.15 to 4.56
mean (SD)	3.35 (6.18)
Number of samples below LoQ (0.1ng/ml)	12
Minimum value (ng/ml)	0.07
Maximum value (ng/ml)	49.6
Creatinine-adjusted urinary BPA at visit 2 (n = 99)	
median (IQR)	1.31 (2.24)
95% confidence intervals	1.46 to 8.34
mean (SD)	4.90 (16.8)
Number of samples below LoQ (0.1ng/ml)	12
Minimum value (ng/ml)	0.04
Maximum value (ng/ml)	139.33
Unadjusted ΔBPA (n = 94)	
median (IQR)	0.14
95% confidence intervals	0.15 to 2.41
mean (SD)	1.28 (5.79)
Minimum value	-8.02
Maximum value	49.5
Adjusted ΔBPA (n = 94)	
median (IQR)	0.02 (2.61)
95% confidence intervals	-0.23 to 6.53
mean (SD)	3.15 (16.5)
Minimum value	-8.6

For peer review only

For peer review only - http://bmjopen.bmj.com/site/about/guidelines.xhtml

Maximum value	133.45
BPA risk score (n = 99)	
median (IQR)	15.0 (10.3)
95% confidence intervals	15.5 to 18.4
mean (SD)	17.0 (7.12)
Demographics (n= 99)	
Sex - % male	44
Exposure to estrogens - % of cohort	14
BMI– median (IQR)	20.7 (3.45)
BMI– mean (SD)	21.2 (3.07)

BPA was detected in the urine of 86% of subjects at visit 1 prior to the intervention. Missing samples were due to non-attendance of participants or non-provision of a suitable sample. Samples below the limit of quantification of 0.1ng/ml were scored as 0.07 ng/ml (LoQ/√2).

Creatinine-adjusted urinary BPA concentrations do not change significantly after following an intervention diet designed to reduce BPA exposure for 7 days.

The median change in creatinine-adjusted urinary BPA between visits (ΔBPA) was 0.02 ng/ml with an interquartile range of 2.61 ng/ml. We identified no changes in urinary BPA between visits ($p = 0.25$; figure 1a). 3 outliers with very high urinary BPA readings at visit 2 were excluded from the analysis, since these samples lay outside the linear range of analysis, so confidence in quantification was poor. No confounding factors included in the analysis were associated with change in BPA (p = 0.78, 0.43 and 0.36 for sex, time of sample collection and BMI respectively). We also identified no change in BPA levels between visits using data uncorrected for creatinine ($p = 0.20$).

Similarly, no relationship between change in urinary BPA (ΔBPA) and BPA risk score was identified (beta coefficient 0.08, standard error 0.07, $p = 0.55$; figure 1b). No associations were noted between change in urinary BPA and BPA risk score in data not adjusted for

For peer review only

creatinine ($p = 0.27$). We found no association between ΔBPA and BPA risk score when considering only the exposure on the day prior to testing, taking into account the short half-life of BPA ($p = 0.16$ and $p = 0.33$ for adjusted and unadjusted data respectively). We conclude that the 'real world' diet designed to reduce BPA exposure had no effect on creatinine-adjusted urinary BPA concentrations in our cohort over a period of 7 days in our dataset.

Participants with highest starting urinary BPA levels were more likely to demonstrate lower BPA levels at visit 2.

We found an inverse relationship between initial BPA levels and whether a participant had reduced BPA levels at visit 2 ($p = 0.003$). These data indicate that the participants in the cohort with the highest creatinine-adjusted urinary BPA levels at visit 1 were more likely to demonstrate a drop in their urinary BPA at visit 2 (figure 2).

Following the intervention diet has significant effects on participant lifestyle

Participants indicated that following the diet had no significant cost implications on family finances, with 50% of participants reporting that it had cost more, and 50% reporting that costs had decreased or remained the same. Although participants did not spend longer preparing their food, 78% of participants reported that their shopping took longer. 58% of participants reported that the diet did not affect their calorific intake. 91% of participants reported that they felt at least slightly restricted in their food choices. 27% of participants

For peer review only

reported that they felt very restricted. Finally, 66% of participants stated that they would find it hard or very hard to follow the diet long term.

Qualitative analysis of the effect of following the diet on lifestyle

We identified 5 overriding themes in our qualitative analysis of the effect of following the diet on lifestyle. These were 1) the widespread use of plastics possibly containing BPA in food packaging ("almost everything is packaged in plastic" – participant 70, "Literally everything involved plastic" – participant 28). 2) Lack of clarity in labelling of products and packaging potentially containing BPA ("I found it really hard to know what foods I could eat … there is never a guarantee it is BPA free" – participant 43, " The biggest problem was that a lot of packaging doesn't state what type of plastic it is or whether it contains BPA" – participant 74). 3) The perceived restrictions of being on the 'real world' BPA avoidance diet ("Difficulty eating out, hard to find foods in college or 'out' that hadn't touched BPA. My family had a takeaway on Saturday night and I couldn't eat it" – participant 56, "Sometimes I can't eat / drink what I want because of the recycling number" – participant 112). 4) The impact of eating 'BPA free' was the only positive theme emerging ("I feel I have eaten much more healthily this week … I didn't eat so much junk food" – participant 74, "I ate more vegetables and less chocolate" – participant 83). 5) The impact on shopping habits ("You can't get it all from supermarkets" – Participant 37; "Had to go to more individual food shops" – participant 103).

DISCUSSION

For peer review only

Exposure to the endocrine disrupting chemical Bisphenol A (BPA) is ubiquitous [2], with growing evidence that it may be associated with adverse health outcomes [4]. Here, 104 researcher participants aged 17-19 years designed and undertook a quantitative and qualitative engaged research project designed to assess the potential for reduction of personal exposure to BPA through moderation of diet, which would have utility in a 'real world' setting.

Although levels of urinary BPA in our study cohort were slightly lower at the outset of the study in our cohort than in others [13], measureable levels were present in the vast majority of our participants. Participants were unable to achieve a reduction in their urinary BPA levels over the 7 day trial period, despite good compliance to supplied guidelines. Avoidance of BPA was not easily achieved on an individual level in our study population, with qualitative analysis indicating that participants experienced feelings of restriction and difficulties in sourcing BPA-free food due to inadequate labelling of foods and food packaging. This suggests that the intervention would be difficult to sustain in the longer term.

For peer review only

This work represents the largest group of unrelated participants in one of the highest exposure demographics to date, since previous work has focused on families and related individuals [11] [12], who may share common sources of BPA. Our intervention is a 'real world' diet, designed to a set of guidelines (such as reduction in the usage of tinned foods or foods with high levels of processing), rather than the strict, prescribed diets that have been used in other studies [11], which suggested that it was possible for participants to reduce their urinary BPA excretion by approximately 60% in a period of just 3 days [11]. In our self-designed, self-administered study this was unachievable. This may reflect the difficulty in identifying and

sourcing foods free of BPA in our commercial environment. Finally, the qualitative thematic analysis we carried out in our study has given an indication that adherence to even a 'real world' BPA reduction diet with fewer restrictions and more choice over the longer term was unlikely in our study population due to difficulties in identifying foodstuffs likely to contain less BPA.

BPA has a terminal half-life of 6 hours [10]. Spot samples may therefore not be as accurate as continuous sampling strategies (24hr urine collection). However, recent studies suggest that despite its short half-life, measureable BPA remains present for up to 43 hours post-fasting, indicating non-food exposures or accumulation in body tissues such as fat [15]. We identified no impact of time of sample collection on BPA levels in our sample set, in either creatinine-adjusted or unadjusted data, indicating that our measurements were not influenced by time since the last meal. Spot sampling as used here may therefore represent an acceptable compromise and remains a practical option in the community setting of our study.

Calculating an accurate BPA risk score is challenging. Data were self-reported, and foodstuffs are not labelled for BPA content. It is difficult to generalise across food types and large variations in BPA concentrations occur between different products of the same food type or even different lots of the same product [1]. Foods that were free of BPA-containing packaging (as far as it was possible to tell) may have been highly processed or contain food items from a variety of sources. Highly processed and 'fast' food has previously been demonstrated to be a source of BPA [16]. A study of the temporal trends seen in composite food samples found no change in the overall BPA content of the food, despite large reduction in the BPA content of some individual food items, illustrating the difficulties in effectively

For peer review only

excluding BPA from a varied diet [17]. Participants may therefore have changed BPA containing foods for other, perceived healthier choices, which may still contain BPA by virtue of processing.

BPA enters foodstuffs by leaching from polycarbonate or epoxy resin after manufacture, or by hydrolysis of the polymer itself [18]. The migration rate of BPA increases with higher temperatures [19], and with time and use, e.g. repeated use of polycarbonate water bottles [20]. Exposure to BPA can also occur through routes other than food, including dust ingestion and dermal absorption [21] and this was not taken into account in our study. A study of volunteers who purposefully handled thermal receipts showed an increase in urinary BPA excretion of up to 84%, and their BPA levels took longer to return to pre exposure levels, suggesting a difference in the bio-availability of BPA through skin and oral routes [22]. We may also have been underpowered to detect subtle changes in urinary BPA, given the heterogeneity in food choice; detection of such effects may need thousands of participants. Finally, our study, like other studies of its type, does not take account of inter-individual differences in the metabolism and excretion of BPA arising from differences in genetic background between people. BPA is metabolised primarily by UDP-glucuronosyltransferases, and altered activity polymorphisms of these enzymes have been reported [23].

Emerging evidence suggests that that BPA may be linked to several chronic human health conditions [24-28], suggesting that continued study of the human health effects of BPA exposure is justified. The opinion of the European Food Safety Authority (EFSA), is that whilst uncertainty over the human health effects of BPA exists, caution should be exercised

For peer review only

in ingestion of BPA [3]. Our data suggests that in our study population, it is unlikely that participants could moderate their own BPA exposure in the long term by self-directed modification of diet in a ‘real world’ setting, and furthermore, participants would have been reluctant to adopt such a lifestyle change in the longer term due to the restrictions in dietary choice and the effects on day to day life. Most of these barriers appear to arise from the pervasiveness of BPA in our food chain, and inadequate labelling of foods packaged in BPA-containing substances. We propose that until a definitive assessment of the health risks of BPA is available, informed choice over whether or not to consume BPA and similar chemicals in foodstuffs should be facilitated by better labelling.

CONTRIBUTORSHIP STATEMENT

TSG - Contributed to study design and co-wrote the paper

NB - Contributed to study design and participant involvement

BP - Managed the technical aspects of the project and reviewed the manuscript

ALC - Contributed to data entry and interpretation and reviewed the manuscript

BPA Schools Study Consortium members - designed and interpreted the study and contributed to the manuscript.

MHS - Carried out the qualitative analysis and reviewed the manuscript

AMS - Managed sample collection, contributed to study design and reviewed the manuscript.

LWH - PI , managed the study, wrote the manuscript

ACKNOWLEDGEMENTS

For peer review only

We acknowledge the work of the NIHR Exeter Clinical Research Facility in aiding the collection of the urine samples.

COMPETING INTERESTS

The authors have no competing interests to declare.

DATA SHARING STATEMENT

Extra data is available upon reasonable request by emailing Lorna Harries (L.W.Harries@exeter.ac.uk).

FUNDING

This study was funded by a Wellcome Trust People Award to LWH and TG (grant number 105162/Z/14/Z).

REFERENCES

For peer review only

1 Geens T, Aerts D, Berthot C*, et al.* A review of dietary and non-dietary exposure to bisphenol-A. *Food Chem Toxicol* 2012;**50**:3725-40.

2 WHO. World Health Organisation Background paper on mechanisms of action of bisphenol A and other biochemical/molecular interactions. *WHO/HSE/FOS* 2010;**11.1**.

3 EFSA. Scientific opinion on the risks to public health related to the presence of bisphenol A (BPA) in foodstuffs. *EFSA journal* 2015;**13**.

4 Rochester JR. Bisphenol A and human health: a review of the literature. *Reprod Toxicol* 2013;**42**:132-55.

5 WHO. Joint FAO/WHO Expert meeting to review toxicological and health aspects of bisphenol A Summary report. 2010.

6 Vandenberg LN, Chahoud I, Heindel JJ*, et al.* Urinary, circulating, and tissue biomonitoring studies indicate widespread exposure to bisphenol A. *Environ Health Perspect* 2010;**118**:1055-70.

7 Gore AC, Chappell VA, Fenton SE*, et al.* EDC-2: The Endocrine Society's Second Scientific Statement on Endocrine-Disrupting Chemicals. *Endocr Rev* 2015;**36**:E1-E150.

8 Agency EC. AGREEMENT OF THE MEMBER STATE COMMITTEE ON THE IDENTIFICATION OF 4,4'-ISOPROPYLIDENEDIPHENOL (BISPHENOL A) AS A SUBSTANCE OF VERY HIGH CONCERN 2017.

9 Lakind JS, Naiman DQ. Daily intake of bisphenol A and potential sources of exposure: 2005-2006 National Health and Nutrition Examination Survey. *Journal of exposure science & environmental epidemiology* 2011;**21**:272-9.

10 Dekant W, Volkel W. Human exposure to bisphenol A by biomonitoring: methods, results and assessment of environmental exposures. *Toxicol Appl Pharmacol* 2008;**228**:114-34.

For peer review only

11 Rudel RA, Gray JM, Engel CL*, et al.* Food packaging and bisphenol A and bis(2-ethyhexyl) phthalate exposure: findings from a dietary intervention. *Environ Health Perspect* 2011;**119**:914-20.

12 Sathyanarayana S, Alcedo G, Saelens BE*, et al.* Unexpected results in a randomized dietary trial to reduce phthalate and bisphenol A exposures. *Journal of exposure science & environmental epidemiology* 2013;**23**:378-84.

13 Calafat AM, Ye X, Wong LY*, et al.* Exposure of the U.S. population to bisphenol A and 4-tertiary-octylphenol: 2003-2004. *Environ Health Perspect* 2008;**116**:39-44.

14 Hornung R, Reed L. Estimation of average concentration in the presence of nondetectable values. *Appl Occupat Environ Hyg* 1990;**5**:46–51.

15 Stahlhut RW, Welshons WV, Swan SH. Bisphenol A data in NHANES suggest longer than expected half-life, substantial nonfood exposure, or both. *Environ Health Perspect* 2009;**117**:784-9.

16 Zota AR, Phillips CA, Mitro SD. Recent Fast Food Consumption and Bisphenol A and Phthalates Exposures among the U.S. Population in NHANES, 2003-2010. *Environ Health Perspect* 2016;**124**:1521-8.

17 Cao XL, Perez-Locas C, Robichaud A*, et al.* Levels and temporal trend of bisphenol A in composite food samples from Canadian Total Diet Study 2008-2012. *Food Addit Contam Part A Chem Anal Control Expo Risk Assess* 2015;**32**:2154-60.

18 Aschberger K, Castello P, Hoekstra E*, et al.* Bisphenol A and baby bottles: challenges and perspectives. 2010.

19 Le HH, Carlson EM, Chua JP*, et al.* Bisphenol A is released from polycarbonate drinking bottles and mimics the neurotoxic actions of estrogen in developing cerebellar neurons. *Toxicol Lett* 2008;**176**:149-56.

For peer review only

20 Brede C, Fjeldal P, Skjevrak I, *et al.* Increased migration levels of bisphenol A from polycarbonate baby bottles after dishwashing, boiling and brushing. *Food Addit Contam* 2003;**20**:684-9.

21 Myridakis A, Chalkiadaki G, Fotou M, *et al.* Exposure of Preschool-Age Greek Children (RHEA Cohort) to Bisphenol A, Parabens, Phthalates, and Organophosphates. *Environ Sci Technol* 2016;**50**:932-41.

22 Lv Y, Lu S, Dai Y, *et al.* Higher dermal exposure of cashiers to BPA and its association with DNA oxidative damage. *Environ Int* 2017;**98**:69-74.

23 Stingl JC, Bartels H, Viviani R, *et al.* Relevance of UDP-glucuronosyltransferase polymorphisms for drug dosing: A quantitative systematic review. *Pharmacol Ther* 2014;**141**:92-116.

24 Galloway T, Cipelli R, Guralnik J, *et al.* Daily bisphenol A excretion and associations with sex hormone concentrations: results from the InCHIANTI adult population study. *Environ Health Perspect* 2010;**118**:1603-8.

25 Melzer D, Rice NE, Lewis C, *et al.* Association of urinary bisphenol a concentration with heart disease: evidence from NHANES 2003/06. *PLoS One* 2010;**5**:e8673.

26 Song Y, Chou EL, Baecker A, *et al.* Endocrine-disrupting chemicals, risk of type 2 diabetes, and diabetes-related metabolic traits: A systematic review and meta-analysis. *J Diabetes* 2015.

27 Savastano S, Tarantino G, D'Esposito V, *et al.* Bisphenol-A plasma levels are related to inflammatory markers, visceral obesity and insulin-resistance: a cross-sectional study on adult male population. *J Transl Med* 2015;**13**:169.

28 Melzer D, Osborne NJ, Henley WE, *et al.* Urinary bisphenol A concentration and risk of future coronary artery disease in apparently healthy men and women. *Circulation* 2012;**125**:1482-90.

For peer review only

For peer review only

FIGURE LEGENDS

Figure 1. The effect of a 'real world' BPA avoidance diet on urinary BPA exposure over a 7 day period. **A**. Urinary BPA levels (ng/ml) adjusted for urinary creatinine were plotted at visit 1 before the intervention and at visit 2 after the intervention. The 3 extreme outliers have been removed. The trajectories of individual participant measurements are shown. **B**. Change in urinary BPA levels in ng/ml following the intervention diet are plotted against the self-reported BPA risk score.

Figure 2. The effect of baseline urinary BPA on the probability of achieving a drop in levels following the intervention. This graph illustrates the median urinary BPA level adjusted for creatinine at visit 1 prior to the intervention expressed relative to whether or not a reduction in urinary BPA levels was achieved following the 7 day intervention diet at visit 2. Error bars refer to the interquartile range of measurement.

For peer review only

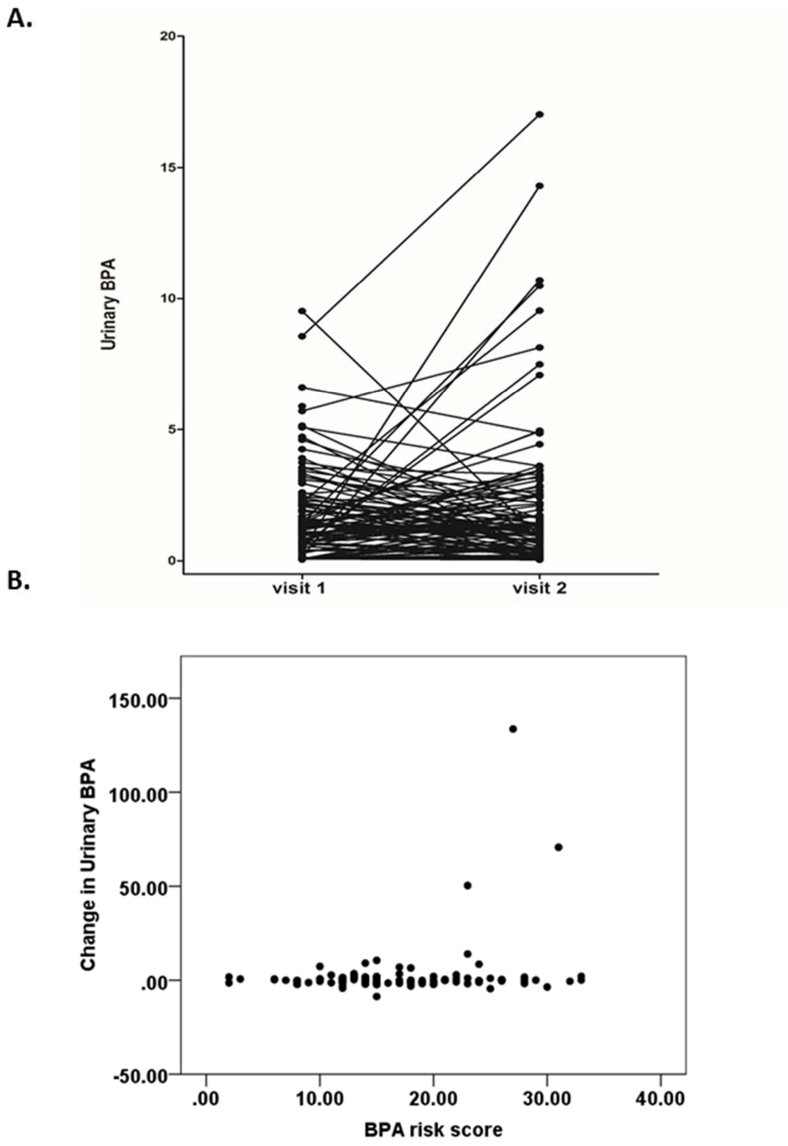

190x254mm (96 x 96 DPI)

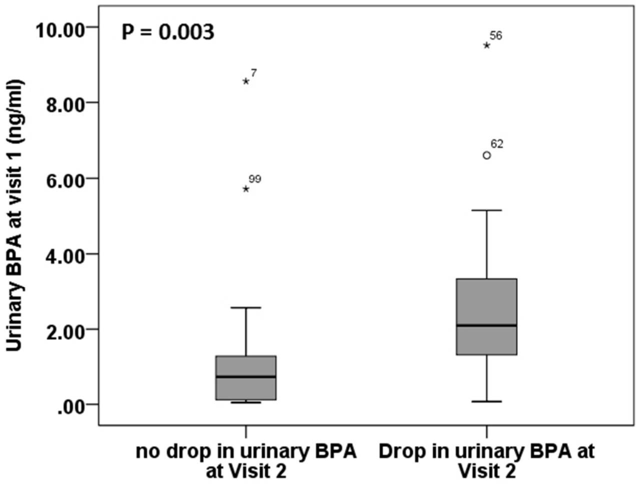

254x190mm (96 x 96 DPI)

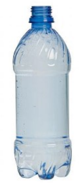

BPA: Myth and Reality
Dietary Intervention Guidelines

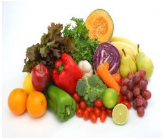

General instructions

The purpose of this dietary intervention trial is to follow a diet designed to minimise routes of exposure to the food packaging chemical bisphenol A (BPA). For the dietary intervention period, please follow as closely as possible the instruction given below. Try to maintain your diet during the intervention period to be as closely similar to your normal diet as possible, in terms of the content, amount and calorific value of the food you eat. Please record details of each meal and the drinks and snacks you consume on the forms provided. Below are some general cooking and eating tips and an indication of which foods are best to avoid and those that are considered a low source of BPA.

For peer review only

Cooking and eating tips for the intervention period.

The general approach is to replace any food items that fall into the 'avoidance' category with an alternative, chosen to minimise exposure to BPA

- **Switch to stainless steel and glass food storage and drink containers.**
- **Move foods to ceramic or glass food containers before microwaving.**
- **Consider a coffee filter or percolator for coffee – home coffee makers (Such as Nespresso^{TM}) may have polycarbonate-based water tanks and phthalate-based tubing.**
- **Eat out less, especially at restaurants that do not use fresh ingredients.**
- **Avoid canned food consumption. Where possible, replace with fresh produce or cardboard or tetrapack packaged alternatives.**
- **Choose fresh fruits and vegetables when possible, and frozen if not.**
- **Soak dried beans for cooking rather than tinned.**

Foods to avoid

Tinned foods. Top ten tinned foods that are reported to be sources of BPA include coconut milk, soup, meat, vegetables, meals (e.g. pasta with sauce), juice, fish, beans, meal replacement drinks, fruit.

Carbonated/fizzy drinks and juices in cans. Avoid carbonated drinks in cans and drinks stored for prolonged periods in reusable sports bottles, unless they are labelled ‘BPA free’ (many commercial sports bottles are).

Fast food from commercial outlets. Most processed food has passed through numerous processes, and each additional processing step provides an opportunity for BPA to enter through packaging or tubing. Try to replace fast and processed foods with a freshly prepared and cooked alternative.

Packaged fruit and vegetables. Replace these where possible with unpackaged, loose fruit and vegetable items as far as possible.

Convenience/ready meals. Plastics types considered safest in terms of chemical migration are recycling numbers 2 and 5. Avoid food prepared in packaging with recycling number 7, which includes many different types of polymer and mixed polymers, including polycarbonate, a source of BPA. Try to avoid foods that are designed to be heated in the microwave in their packaging.

Chocolate and ice cream. Individuals who report eating chocolate bars and ice cream on a regular basis have been reported to have higher than average BPA exposure. Try to avoid excessive consumption.

Non-food or food packaging routes of exposure

Although plastics found in consumer goods such as DVDs, CDs, computer goods and sunglasses do contain BPA, this is not an important route of exposure.

Till receipts often contain high levels of BPA, so wash your hands before eating or drinking if you have been handling them.

Dental sealants may contain BPA, so avoid any pre-planned dental work

Supported by wellcome trust

UNIVERSITY OF EXETER | MEDICAL SCHOOL

NHS National Institute for Health Research

Example daily diet

Food Item	Comments
**Breakfast**	
Cereal, Fruit	
Milk	Polypropylene or glass packaging
Bread	
Yoghurt	Choose polypropylene container
**Lunch**	
Meat or fish products	Check packaging and avoid those labelled no. 7. Avoid tinned ingredients
Cheese	
Salad items, Fruit	Choose unpackaged where possible, wash before use
Pasta	
**Dinner**	
Shepherds pie	Cooked in saucepan and oven rather than microwaved in plastic
Green beans	Fresh or frozen
Bread	
**Drinks**	
Water	Water direct from tap or use stainless steel or BPA free water bottle
Tea/coffee	Prepare in teapot or cafetiere, avoid commercial coffee makers
Carbonated drinks	Avoid canned drinks and those stored in reusable containers for prolonged periods
Milk	Polypropylene or glass packaging
**Snacks**	
Fruit	
Potato crisps	

For peer review only

Place participant barcode here	FOOD - DAY 1		DRINK – DAY 1	
**Contents of meal**	Was food packaged in plastic known or suspected to contain BPA?	Was food cooked in plastic known or suspected to contain BPA?	**Type of Drink**	Packaging (please describe) e.g. canned, plastic (Δ number), tetrapak
**Breakfast:**				
1.				
2.				
3.				
4.				
5.				
**Lunch**:				
1.				
2.				
3.				
4.				
5.				
**Dinner**:				
1.				
2.				
3.				
4.				
5.				
**Snacks**:				
1.				
2.				
3.				
4.				

For peer review only

Place participant barcode here	FOOD - DAY 2		DRINK – DAY 2	
**Contents of meal**	Was food packaged in plastic known or suspected to contain BPA?	Was food cooked in plastic known or suspected to contain BPA?	**Type of Drink**	Packaging (please describe) e.g. canned, plastic (Δ number), tetrapak
**Breakfast:**				
1.				
2.				
3.				
4.				
5.				
**Lunch**:				
1.				
2.				
3.				
4.				
5.				
**Dinner**:				
1.				
2.				
3.				
4.				
5.				
**Snacks**:				
1.				
2.				
3.				
4.				

For peer review only

Place participant barcode here	FOOD - DAY 3		DRINK – DAY 3	
**Contents of meal**	Was food packaged in plastic known or suspected to contain BPA?	Was food cooked in plastic known or suspected to contain BPA?	**Type of Drink**	Packaging (please describe) e.g. canned, plastic (Δ number), tetrapak
**Breakfast:**				
1.				
2.				
3.				
4.				
5.				
**Lunch**:				
1.				
2.				
3.				
4.				
5.				
**Dinner**:				
1.				
2.				
3.				
4.				
5.				
**Snacks**:				
1.				
2.				
3.				
4.				

For peer review only

Place participant barcode here	FOOD - DAY 4		DRINK – DAY 4	
**Contents of meal**	Was food packaged in plastic known or suspected to contain BPA?	Was food cooked in plastic known or suspected to contain BPA?	**Type of Drink**	Packaging (please describe) e.g. canned, plastic (Δ number), tetrapak
**Breakfast:**				
1.				
2.				
3.				
4.				
5.				
**Lunch**:				
1.				
2.				
3.				
4.				
5.				
**Dinner**:				
1.				
2.				
3.				
4.				
5.				
**Snacks**:				
1.				
2.				
3.				
4.				

For peer review only

Place participant barcode here	FOOD - DAY 5		DRINK – DAY 5	
**Contents of meal**	Was food packaged in plastic known or suspected to contain BPA?	Was food cooked in plastic known or suspected to contain BPA?	**Type of Drink**	Packaging (please describe) e.g. canned, plastic (Δ number), tetrapak
**Breakfast:**				
1.				
2.				
3.				
4.				
5.				
**Lunch**:				
1.				
2.				
3.				
4.				
5.				
**Dinner**:				
1.				
2.				
3.				
4.				
5.				
**Snacks**:				
1.				
2.				
3.				
4.				

For peer review only

Place participant barcode here	FOOD - DAY 6		DRINK – DAY 6	
**Contents of meal**	Was food packaged in plastic known or suspected to contain BPA?	Was food cooked in plastic known or suspected to contain BPA?	**Type of Drink**	Packaging (please describe) e.g. canned, plastic (Δ number), tetrapak
**Breakfast:**				
1.				
2.				
3.				
4.				
5.				
**Lunch**:				
1.				
2.				
3.				
4.				
5.				
**Dinner**:				
1.				
2.				
3.				
4.				
5.				
**Snacks**:				
1.				
2.				
3.				
4.				

For peer review only

Place participant barcode here	FOOD - DAY 7		DRINK – DAY 7	
**Contents of meal**	Was food packaged in plastic known or suspected to contain BPA?	Was food cooked in plastic known or suspected to contain BPA?	**Type of Drink**	Packaging (please describe) e.g. canned, plastic (Δ number), tetrapak
**Breakfast:**				
1.				
2.				
3.				
4.				
5.				
**Lunch**:				
1.				
2.				
3.				
4.				
5.				
**Dinner**:				
1.				
2.				
3.				
4.				
5.				
**Snacks**:				
1.				
2.				
3.				
4.				

Participant barcode

Additional study information.

Please do not feel obliged to answer these questions if you are uncomfortable doing so.

Gender

☐ Female
☐ Male
☐ Prefer not to say

Tobacco Usage – Have you used tobacco over the past week

☐ Yes
If so, what type and how much?______________________________
☐ No
☐ Prefer not to say

Alcohol Usage – Have you used alcohol over the past week

☐ Yes
If so, what type and how much?______________________________
☐ No
☐ Prefer not to say

Medication- Have you taken any medication over the last week?

☐ Yes
☐ No
☐ Prefer not to say

If so, Please name the medication______________________ ☐ Prefer not to say

Vegetarian/vegan diet - Have you eaten or drank any soya products over the past week?

☐ Yes
☐ No
☐ Prefer not to say

Your measurements - leave blank if you prefer not to say

Your height ____________________________

Your weight____________________________

Supported by wellcome trust | University of Exeter Medical School | NHS National Institute for Health Research

For peer review only - http://bmjopen.bmj.com/site/about/guidelines.xhtml

Participant Barcode

BPA: Myth and Reality diet questionnaire

1. Were there any times during the week that you knowingly/unknowingly did not stick to the diet? Please tick any that apply and give indication of frequency.

School meals ☐ ____ times

Restaurants/cafés ☐ ____ times

Friends' houses ☐ ____ times

Takeaway ☐ ____ times

Other ______________________________ ☐ ____ times

2. If you heated your food in a microwave, what was the food in? Tick any which apply and give indication of frequency.

A food storage container or bowl known or suspected to contain BPA ☐____times

3. When you or your family drank water, where did your water come from? Tick any which apply and give indication of frequency.

Plastic filter jug known or suspected to contain BPA ☐____times

Individual water bottle known or suspected to contain BPA ☐____times

Larger water container known or suspected to contain BPA ☐____times

4. How many times during the week did you eat food that had been stored or transported in plastic containers known or suspected to contain BPA?

____

5. How many times during the week did you eat tinned food or drink from cans?

____

6. Did the BPA reduced diet affect How much you spent on shopping?

Spent more ☐

Spent less ☐

No difference ☐

Participant questionnaire V3 25Jun15

For peer review only

Participant Barcode

7. Did it take longer to source your food than usual?

Yes ☐ No ☐

If so, why? ______________________________

____

8. Did it take longer to prepare food than usual?

Yes ☐ No ☐

If so, why? ______________________________

____

9. How restricted did you feel by your food choice?

☐ Very ☐ Slightly ☐ No difference

If you felt you were restricted by the diet, why was this? ______________________________

____

10. Did the diet affect your calorific intake?

Yes ☐ No ☐

If so, why? ______________________________

____

For peer review only - http://bmjopen.bmj.com/site/about/guidelines.xhtml

Participant Barcode

11. How easy would you find it to sustain this diet over a longer period of time?

Very easy ☐

Easy ☐

Hard ☐

Very hard ☐

Not sure ☐

12. Is there anything else about following the diet that you would like to add?

Supported by wellcome trust

UNIVERSITY OF EXETER | MEDICAL SCHOOL

NHS National Institute for Health Research

For peer review only - http://bmjopen.bmj.com/site/about/guidelines.xhtml

Thank you for reading this leaflet. If you wish to participate in this study, you will be asked to agree to the consent statements below in the presence of a member of the research team.

CONSENT STATEMENTS

1. I confirm that I have read this information sheet and have discussed participation in this project with my family. I have had opportunity to consider the information, ask questions and have had these questions answered satisfactorily. *You should not give consent until you are happy that you understand what the study involves.*

2. I understand that my participation is voluntary and that I am free to withdraw at any time without giving any reason, without my right to participate in the rest of the study being affected. *This means that even if you helped design this study you do not have to be a participant and you should not feel under any pressure to participate.*

3. I agree to participate in this study as a research subject. *This means that you agree to participate in a one-week diet and to provide two blood and urine samples.*

4. I understand that my anonymised blood and urine samples and linked anonymous questionnaire data will be sent to University of Exeter Medical School, Royal Devon & Exeter Hospital and my urine sample only will be sent to the Rolvaltain laboratory, a specialist BPA analysis company. *This means that laboratory staff will not know that samples belong to you, but dedicated staff at the University of Exeter, with training and experience in data protection ,will be able to link your sample data to your questionnaire data.*

5. I understand that RNA (genetic material) will be extracted from my blood and will be stored anonymously. *This means that Professor Harries' team will use our RNA to provide data that you will help analyse but may also do further research on the samples to identify reasons for any changes seen.*

6. I understand that data relating to my participation in the study will be returned anonymously to my school to be used for educational purposes. *This means that although you will get to analyse data from your samples there is no way you will know which data relates to your samples and which to other participants*.

Complaints:

If you have any complaints about the way in which this study has been carried out please contact the Chair of the University of Exeter Medical School Research Ethics Committee Peta Foxall PhD, Chair, UEMS Research Ethics Committee: P.J.D.Foxall@exeter.ac.uk.

This project has been reviewed and approved by the University of Exeter Medical School Research Ethics committee UEMS REC REFERENCE NUMBER: 15/07/074)

Information Sheet - Version 4 (2/8/15)

BPA: Myth or Reality?

A research study investigating the effect of chemicals in plastic on gene activity and whether dietary interventions can reduce BPA levels in teenagers.

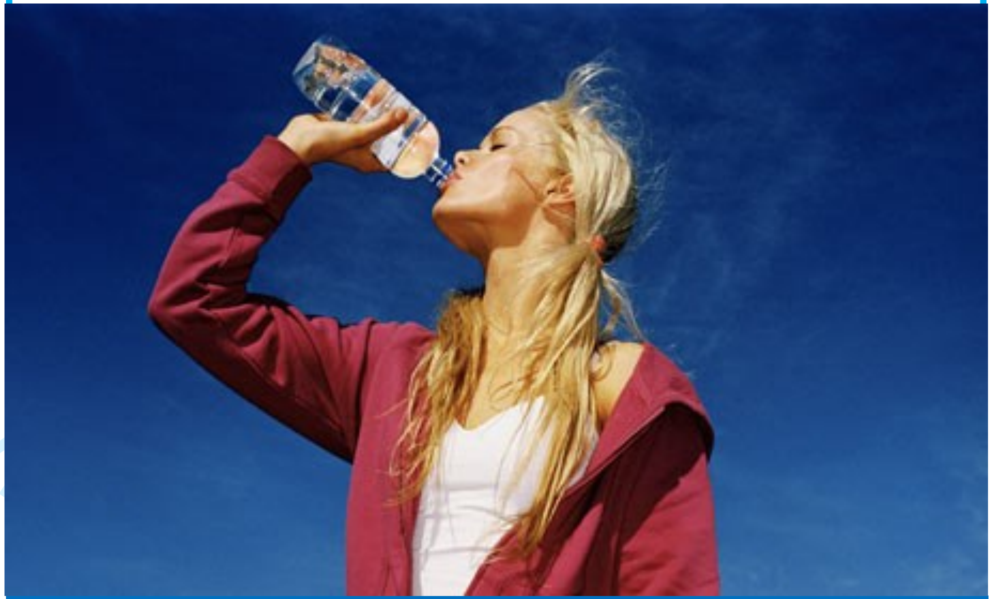

Involvement & Engagement

The aim of this year-long project is to involve teenagers in a research study that is relevant to them, by allowing them to help design a research project, analyse non-identifiable participant data and help to present and publish the outcomes.

Participation

Students will be asked to undertake a one week diet to reduce their intake of BPA, a chemical found in plastics. They will be asked to provide urine and blood samples before and after their diet.

Supported by wellcome trust
UNIVERSITY OF EXETER | MEDICAL SCHOOL
NHS National Institute for Health Research

For peer review only - http://bmjopen.bmj.com/site/about/guidelines.xhtml

What is BPA?

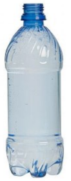

BPA (Bisphenol A) is a chemical used in the manufacture of plastics. Plastics containing BPA are found in a wide range of products including food and drink containers. BPA in these products can be ingested and there are concerns that high BPA levels in the blood could possibly affect human health. Research is therefore needed to understand its effects on the human body and how we can reduce its consumption by minor changes to our diet.

This project is being run as a student-involvement project to answer two specific questions:

1. **Can we see the effects of dietary BPA on our genes?**
2. **Can we effectively reduce BPA in our diet?**

In the past, small-scale experiments have shown that BPA levels in the human body can be reduced by rigid dietary interventions but these interventions would be difficult to implement in the "real world". In this study a one-week dietary intervention designed by teenagers will be used by them to determine whether BPA levels, and the activity of BPA-responsive genes can be effectively reduced in young people by avoiding food packaging that contains this chemical.

What will I need to do?

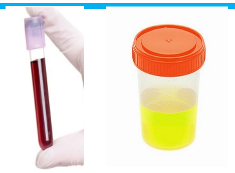

M

Day 1

- Provide a nurse with a 2.5ml blood sample and a urine sample.

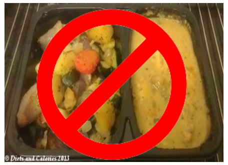

T

W

T

Day 2 - Day 8

- Follow a diet that you have helped to design .
- The diet will exclude sources of BPA as much as possible but will be nutritionally and calorifically similar to your usual diet.
- You will be asked to complete a food diary and answer a questionnaire about how easy it was to follow this diet.

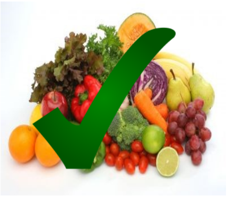

F

S

S

Day 8

- Provide a nurse with a 2.5ml blood sample and a urine sample.

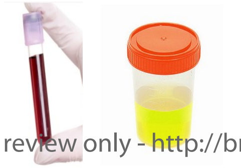

M

We recommend that you discuss the project with your family and involve them in planning what you eat and how you will prepare it.

What will happen to my samples and data?

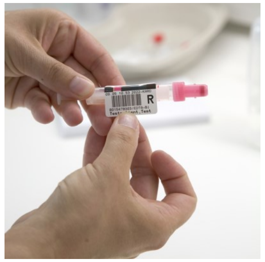

When you participate in the study you will be allocated with a numerical study ID. Your samples and data will be labelled with this number so that we can match your 'before' and 'after' diet samples with your food diary data. Once all data has been collated and coded it will be further anonymised by a person external to the project so that no data can be linked to any of the participants.

Urine samples from before and after the diet will be sent to the Royal Devon & Exeter NHS Foundation Trust for creatinine analysis and to the Rolvaltain laboratory for BPA analysis. RNA will be extracted from blood samples at the Royal Devon & Exeter Molecular Genetics Laboratory and the expression levels of two BPA-responsive genes will be measured in the samples taken before and after the diet. These anonymised RNA samples will be stored and used only by Professor Harries team for further research on the mechanisms behind our findings.

What are the benefits of taking part?

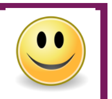

This project will help you to understand how you might be able to reduce BPA in your diet and your involvement in the design will give you an excellent insight into clinical research, community outreach and scientific practise. Your role as a participant is unlikely to have any direct health benefits.

Are there any risks in taking part?

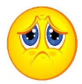

Blood samples will be taken by fully qualified and insured NHS personnel. Any potential discomfort or side-effects will be equivalent to that experienced giving a blood sample to your GP. All data will be fully anonymised before analysis. This means that you will not find out anything about your blood or urine samples. Following the diet may minimally increase the cost of your groceries for the week, but since fresh foods are usually less expensive than pre-packaged foods, we do not expect this to be an issue.

What will happen to the results of the research study?

You will be given the opportunity to help analyse anonymised data from this project and to help disseminate the outcomes of this research. It is hoped that the findings will be published in peer-reviewed journals and the wider media.

Who is organising this research?

The research is organised by Professors Lorna Harries & Tamara Galloway of the University of Exeter as part of their research program into BPA and part of the University's outreach program to involve schools in academic research.

For peer review only - http://bmjopen.bmj.com/site/about/guidelines.xhtml

Version 4 (2/8/2015)

BPA: Myth & Reality

STUDY ID

**CONSENT STATEMENTS**	**Please circle**
1. I confirm that I have read information sheet BPA PIS Version 4 and have discussed participation in this project with my family. I have had opportunity to consider the information, ask questions and have had these questions answered satisfactorily. *You should not give consent until you are happy that you understand what the study involves.*	**YES / NO**
2. I understand that my participation is voluntary and that I am free to withdraw at any time without giving any reason, without right to participate in the rest of the study being affected. *This means that even if you helped design this study you do not have to be a participant and you should not feel under any pressure to participate.*	**YES / NO**
3. I agree to participate in this study as a research subject. *This means that you agree to participate in a one-week diet and to provide two blood and urine samples.*	**YES / NO**
4. I understand that my anonymised blood and urine samples and linked anonymous questionnaire data will be sent to University of Exeter Medical School, Royal Devon & Hospital and my urine sample only will be sent to the Rolvaltain laboratory, a specialist BPA analysis company. *This means that laboratory staff will not know that samples belong to you, but dedicated staff at the University of Exeter, with training and experience in data protection, will be able to link your sample data to your questionnaire data.*	**YES / NO**
5. I understand that RNA (genetic material) will be extracted from my blood and will be stored anonymously. *This means that Professor Harries' team will use our RNA to provide data that you will help analyse but may also do further research on the samples to identify reasons for any changes seen.*	**YES / NO**
6. I understand that data relating to my participation in the study will be returned anonymously to my school to be used for educational purposes. *This means that although you will get to analyse data from your samples there is no way you will know which data relates to your samples and which to other participants*.	**YES / NO**

Name of Participant	Signature	Date
Name of Person Obtaining Consent	Signature	Date

I the above signed testify the participant is providing voluntary and fully informed consent to participate in this study. I am on the delegation log to obtain consent for this study and are trained in obtaining consent.

This project has been reviewed and approved by the University of Exeter Medical School Research Ethics Committee UEMS REC REFERENCE NUMBER: 15/07/074)

Supported by wellcome trust

UNIVERSITY OF EXETER | MEDICAL SCHOOL

NHS National Institute for Health Research

For peer review only - http://bmjopen.bmj.com/site/about/guidelines.xhtml

STROBE Statement—checklist of items that should be included in reports of observational studies

	Item No	Recommendation
**Title and abstract**	1	(*a*) Indicate the study's design with a commonly used term in the title or the abstract
		(*b*) Provide in the abstract an informative and balanced summary of what was done and what was found<br><br>**a) Page 1**<br>**b) Page 2**
**Introduction**		
Background/rationale	2	Explain the scientific background and rationale for the investigation being reported<br><br>**Page 6, line 82**
Objectives	3	State specific objectives, including any prespecified hypotheses<br><br>**Page 7, line 113**
**Methods**		
Study design	4	Present key elements of study design early in the paper<br><br>**Page 8, line 140**
Setting	5	Describe the setting, locations, and relevant dates, including periods of recruitment, exposure, follow-up, and data collection<br><br>**Page 8, line 152**
Participants	6	(*a*) *Cohort study*—Give the eligibility criteria, and the sources and methods of selection of participants. Describe methods of follow-up<br><br>**Page 7, line 123**<br><br>*Case-control study*—Give the eligibility criteria, and the sources and methods of case ascertainment and control selection. Give the rationale for the choice of cases and controls<br>*Cross-sectional study*—Give the eligibility criteria, and the sources and methods of selection of participants
		(*b*) *Cohort study*—For matched studies, give matching criteria and number of exposed and unexposed<br>*Case-control study*—For matched studies, give matching criteria and the number of controls per case
Variables	7	Clearly define all outcomes, exposures, predictors, potential confounders, and effect modifiers. Give diagnostic criteria, if applicable<br><br>Page 7, line 140, Page 8, line 152.

For peer review only - http://bmjopen.bmj.com/site/about/guidelines.xhtml
For peer review only

Data sources/ measurement	8*	For each variable of interest, give sources of data and details of methods of assessment (measurement). Describe comparability of assessment methods if there is more than one group<br><br>**Page 8, line 140, Page 8, line 152**
Bias	9	Describe any efforts to address potential sources of bias<br><br>**Page 8, line 154, page 9 line 165**
Study size	10	Explain how the study size was arrived at<br><br>**Page 7, line 127**
Quantitative variables	11	Explain how quantitative variables were handled in the analyses. If applicable, describe which groupings were chosen and why<br><br>**Page 9, line 157**
Statistical methods	12	(*a*) Describe all statistical methods, including those used to control for confounding<br><br>**Page 9, line 170**
		(*b*) Describe any methods used to examine subgroups and interactions<br><br>**Page 9, line 175**
		(*c*) Explain how missing data were addressed<br><br>**Page 9, line 164**
		(*d*) *Cohort study*—If applicable, explain how loss to follow-up was addressed<br><br>**Page 12, line 206**<br><br>*Case-control study*—If applicable, explain how matching of cases and controls was addressed<br>*Cross-sectional study*—If applicable, describe analytical methods taking account of sampling strategy
		(*e*) Describe any sensitivity analyses<br><br>**N/A**

Continued on next page

For peer review only

**Results**		
Participants	13*	(a) Report numbers of individuals at each stage of study—eg numbers potentially eligible, examined for eligibility, confirmed eligible, included in the study, completing follow-up, and analysed<br><br>**Table 1**
		(b) Give reasons for non-participation at each stage<br><br>**Page 12, line 206**
		(c) Consider use of a flow diagram<br><br>**N/A**
Descriptive data	14*	(a) Give characteristics of study participants (eg demographic, clinical, social) and information on exposures and potential confounders<br><br>**Table 1**
		(b) Indicate number of participants with missing data for each variable of interest<br><br>**Table 1**
		(c) *Cohort study*—Summarise follow-up time (eg, average and total amount)<br><br>**Page 9, line 172**
Outcome data	15*	*Cohort study*—Report numbers of outcome events or summary measures over time<br><br>**Table 1**
		Case-control study—Report numbers in each exposure category, or summary measures of exposure
		Cross-sectional study—Report numbers of outcome events or summary measures
Main results	16	(*a*) Give unadjusted estimates and, if applicable, confounder-adjusted estimates and their precision (eg, 95% confidence interval). Make clear which confounders were adjusted for and why they were included<br><br>**Page 12, line 213 to page 13 line 229**
		(*b*) Report category boundaries when continuous variables were categorized<br><br>**Page 9, line 176**
		(*c*) If relevant, consider translating estimates of relative risk into absolute risk for a meaningful

For peer review only - http://bmjopen.bmj.com/site/about/guidelines.xhtml

		time period<br><br>**N/A**
Other analyses	17	Report other analyses done—eg analyses of subgroups and interactions, and sensitivity analyses<br><br>**Page 13, line 234**
**Discussion**		
Key results	18	Summarise key results with reference to study objectives<br><br>**Page 15, line 276**
Limitations	19	Discuss limitations of the study, taking into account sources of potential bias or imprecision. Discuss both direction and magnitude of any potential bias<br><br>**Page 16, line 298**
Interpretation	20	Give a cautious overall interpretation of results considering objectives, limitations, multiplicity of analyses, results from similar studies, and other relevant evidence<br><br>**Page 17, line 334**
Generalisability	21	Discuss the generalisability (external validity) of the study results<br><br>**Page 18, line 344**
**Other information**		
Funding	22	Give the source of funding and the role of the funders for the present study and, if applicable, for the original study on which the present article is based<br><br>**Page 19, line 374**

*Give information separately for cases and controls in case-control studies and, if applicable, for exposed and unexposed groups in cohort and cross-sectional studies.

Note: An Explanation and Elaboration article discusses each checklist item and gives methodological background and published examples of transparent reporting. The STROBE checklist is best used in conjunction with this article (freely available on the Web sites of PLoS Medicine at http://www.plosmedicine.org/, Annals of Internal Medicine at http://www.annals.org/, and Epidemiology at http://www.epidem.com/). Information on the STROBE Initiative is available at www.strobe-statement.org.

For peer review only

BMJ Open

An engaged research study to assess the effect of a 'real-world' dietary intervention on urinary bisphenol A (BPA) levels in teenagers

Journal:	*BMJ Open*
Manuscript ID	bmjopen-2017-018742.R1
Article Type:	Research
Date Submitted by the Author:	23-Aug-2017
Complete List of Authors:	Galloway, Tamara; University of Exeter, College of Life and Environmental Sciences<br>Baglin, Nigel; Research Projects<br>Lee, Benjamin; University of Exeter Medical School, Institute of Biomedical and Clinical Studies<br>Kocur, Anna; University of Exeter Medical School, Institute of Biomedical and Clinical Studies<br>Shepherd, Maggie; Royal Devon and Exeter National Health Service Foundation Trust, National Institute for Health Research Exeter Clinical Research Facility<br>Steele, Anna; Royal Devon and Exeter National Health Service Foundation Trust, National Institute for Health Research Exeter Clinical Research Facility<br>BPA Schools, Study Consortium; University of Exeter Medical School, Institute of Biomedical and Clinical Studies<br>Harries, Lorna; University of Exeter Medical School, Institute of Biomedical and Clinical Studies
**Primary Subject Heading**:	Public health
Secondary Subject Heading:	Communication
Keywords:	Bisphenol A, Dietary intervention, PUBLIC HEALTH, community, Engaged research

SCHOLARONE™
Manuscripts

An engaged research study to assess the effect of a 'real-world' dietary intervention on urinary bisphenol A (BPA) levels in teenagers

Tamara. S. Galloway[1], Nigel Baglin[2], Benjamin P Lee[3], A.L. Kocur[3], M.H. Shepherd[4], A.M. Steele[4], BPA schools study consortium[#] and L.W. Harries[3]

[1]College of Life and Environmental Sciences, University of Exeter, Exeter UK EX4 4AS.
[2]Research Projects, Exeter, UK, EX2 5DQ.
[3]RNA-Mediated Disease Mechanisms group, Institute of Biomedical and Clinical Sciences, University of Exeter Medical School, University of Exeter, Exeter, UK EX2 5DW.
[4]National Institute for Health Research Exeter Clinical Research Facility, Royal Devon and Exeter National Health Service Foundation Trust, and University of Exeter Medical School, Exeter, U.K.

[#] Consortium members listed in supplementary information file 1

Word count 3021

Correspondence to:

Prof Lorna Harries
University of Exeter Medical School,
Barrack Road,
Exeter, EX2 5DW
Tel =44 1392 406773
Email: L.W.Harries@exeter.ac.uk

Declaration of competing issues

The authors have no competing interests to declare.

For peer review only

ABSTRACT

Objective

Bisphenol A has been associated adverse human health outcomes and exposure to this compound is near-ubiquitous in the Western world. We aimed to examine whether self-moderation of BPA exposure is possible by altering diet in a real-world setting.

Design

An Engaged Research dietary intervention study designed, implemented and analysed by healthy teenagers from 6 schools and undertaken in their own homes.

Participants

104 students aged between 17 and 19 years from schools in the South West of the UK provided diet diaries and urine samples for analysis.

Intervention

Researcher participants designed a set of literature-informed guidelines for reduction of dietary BPA to be followed for 7 days.

Main outcome measure

Creatinine-adjusted urinary BPA levels were taken before and after the intervention. Information on packaging and food/drink ingested was used to calculate a BPA risk score for anticipated exposure. A qualitative analysis was carried out to identify themes addressing long term sustainability of the diet.

Results

For peer review only

BPA was detected in urine of 86% of participants at baseline at a median value of 1.34 ng/ml (IQR 1.82). No effect of the intervention diet on BPA levels was identified overall (p = 0.25), but there was a positive association in those participants who showed a drop in urinary BPA concentration post intervention and their initial BPA level (p = 0.003). Qualitative analysis identified themes around feelings of lifestyle restriction and the inadequacy of current labelling practices.

Conclusions

We found no evidence in this self-administered intervention study that it was possible to moderate BPA exposure by diet in a real world setting. Furthermore, our study participants indicated that they would be unlikely to sustain such a diet long term, due to the difficulty in identifying BPA-free foods.

For peer review only

Article Summary

Strengths of the study

- This study represents the largest assessment to date of the potential for moderating one's own BPA exposure through diet
- The study was carried out in a 'real-world' setting rather than a regulated, controlled environment.
- The study was carried out in teenagers, the demographic with amongst the highest exposure.
- Qualitative analysis reveals challenges with sustaining such a diet.

Limitations of the study

- Calculation of a risk score is challenging due to the pervasive nature of BPA contamination

For peer review only

INTRODUCTION

Bisphenol A is one of the world's highest production volume chemicals. It is used in the manufacture of polycarbonate and other plastic consumer products, in heat resistant papers, dental sealants and in the epoxy resin-based lining of food and drink containers [1]. BPA can be found above the detection limit in the urine of the majority of people worldwide [2]. Concern has been raised for public health, since BPA is classified as an endocrine disrupting chemical (EDC) which has been linked with several disorders in cell and animal models [3-5]. Several epidemiological studies have also linked outcomes such as type 2 diabetes, cardiovascular disease, obesity and abnormalities of sex hormone levels with BPA levels in human populations [6-10] Epidemiological data in humans has historically been more contentious however, due to relatively small sample sizes and issues around causality [11]. The Endocrine Society concluded in 2015 that current evidence suggests that BPA and other endocrine disrupting chemicals may have effects on several reproductive, cardiovascular and metabolic traits in humans [12]. The current opinion of food regulatory bodies such as the European Food Standards Agency (EFSA) is that sufficient uncertainty remains to be able to exclude effects on the reproductive, immune, nervous, metabolic and cardiovascular systems and on cancer development [3] whilst the European Chemicals Agency (ECHA) has recently reclassified BPA as a chemical of very high concern due to its endocrine disrupting properties [13].

There has been wide interest in the sources of BPA and the potential for individuals to reduce their own exposure. Human exposure has been reported from inhalation of dust, uptake across the skin from thermal papers and till receipts and release from dental sealants. The main source is the ingestion of food and drink contaminated with BPA leached from

For peer review only

packaging materials [1, 14]. BPA is rapidly metabolised in the gut wall and liver and removed from the blood by the kidneys, with a terminal half-life of 6 hours after oral ingestion [15]. BPA has been detected in food samples packaged in glass, plastic, paper and paperboard cartons, with an average concentration of 0.46 ng/g, rising to over 700 ng/g for certain canned foods. Conversely, in a dietary intervention study in which 22 volunteers consumed a 3 day fresh food diet which excluded canned or packaged foods, there was a 66% reduction in urinary BPA excretion compared to concentrations before the intervention [16]. This latter study involved full dietary replacement of foodstuffs, an approach which is impractical for the population at large. A follow up study found that households who followed written recommendations produced by health care professionals showed no significant change in their BPA exposure [17].

We present an alternative, citizen-science based approach, where 104 student volunteers designed and undertook their own intervention diet, following provision of educational materials. We questioned whether adherence to a self-designed and self-administered 'real world' diet over 7 days would lead to significant reductions in excreted urinary BPA, and if so, whether such a diet was likely to be sustainable in the long term.

METHODS

Participant group

We chose adolescents because they represent a demographic with high BPA exposure (aggregated exposure of 1.449 µg/kg body weight per day) [3, 18]. 124 students aged 17-19 from local schools were initially invited to participate in this engaged research project, of

For peer review only

which 104 signed up to take part in some form. 94 individuals provided information and samples at both visit one and visit 2 and comprise the complete dataset. This represents the largest intervention study in the population demographic with the highest BPA exposure to date [18]. Study size was arrived upon based on anticipated effect sizes from previous work of this nature [16], and we allowed for a 10% dropout rate. Students designed all of the materials required for completion of the study (study protocols, food diaries, lifestyle questionnaires, patient information sheets and consent forms (see Supplementary Information files 2 to 6).

Ethical Permission

Ethical permission was granted by the University of Exeter Medical School Ethics Committee (reference number 15/07/074) and the study was carried out in accordance with the Declaration of Helsinki.

The intervention diet

Students designed a “real world” diet designed to reduce consumption of BPA by avoidance of processed foods and foods packaged in known sources of BPA [1, 14]; supplementary information file 2). The study was designed at the University of Exeter as a collaboration between academic staff and participating students and was developed at a series of interactive workshops attended by all parties.

For peer review only

Students were asked to minimise their intake of known sources of BPA according to a set of guidelines that had been co-designed with them based on the known literature. We requested that calorific intake was maintained as near to their usual diet as possible and recorded details of their daily diet including all food and drink, and its associated packaging, in a self-reported food diary (Supplementary information file 3). Adherence was assessed using a 'BPA risk score' based on instances of known or suspected exposure for each participant was then calculated, whereby each individual incidence of potential BPA exposure was given a score of 1. These scores were collated at the end of the 7 day trial to give a final risk score. Risk scores from the final day of the intervention only were also considered, since the half-life of BPA means that this is most relevant to the sample collected at visit 2. Information on lifestyle factors including sex, BMI and time of urine collection was also collected (Supplementary information file 4). We recognised that there may be a temptation for students to change their diets before the trial based on their new learning. To avoid this, students were also specifically asked not alter their diet before the intervention.

Sample collection and measurement of urinary BPA

Urine samples were collected into BPA-free bottles (Vacutest Kima, Italy) immediately before and after the intervention, and were frozen at -20˚C within 4 hours. Each participant was sampled twice, once at visit 1 before the intervention and once at visit 2 after the intervention. Sample collections were staggered to allow for the large number of participants passing through the facility, but students were sampled during the same time slot at both visits to account for circadian variation in BPA metabolism. The initial samples were collected during the early part of the day just prior to the students commencing the trial. The second samples were taken over the same time period 7 days later, just prior to the students

For peer review only

recommencing their usual diet. Samples were transported on dry ice to a commercial laboratory (Rovaltain Research Company, Aixain, France) where analysis of total BPA was assessed by gas chromatography-tandem mass spectrometry. Experimental methods were validated for linearity, detection limit and accuracy and specificity of quantification based on the Standard NF T 90-201 for determination of xenobiotics. A quality control check of known standards injected every 6 samples at two levels of concentration (0.5 ng/ml and 5 ng/ml) was quantified with each batch of unknown samples. Water-only samples were included as negative controls. Urinary creatinine was measured at the Royal Devon and Exeter Hospital using the Jaffe method on the Roche P800 platform (Roche, Mannheim, Germany), to allow correction for urine dilution. Results were expressed as a BPA:creatinine ratio. Samples where BPA was detected but quantifying at or around the limits of quantification (LoQ) of 0.1ng/ml were scored as LoQ/√2 according to the method of Hornung and Reed [19].

Statistical Analysis

The difference between urinary BPA adjusted for creatinine between samples taken at visits 1 and 2 was assessed to generate a ΔBPA continuous variable. BPA risk scores were calculated as a continuous variable. The relationship between urinary BPA levels before and after the 7 day intervention was assessed using a repeated-measure ANOVA, adjusted for sex, time of sampling and BMI, with and without correction for creatinine. The relationship between urinary BPA at visit 1 and whether or not the participants had lower BPA at visit 2 was also examined by binary logistic regression, adjusted for sex, time of sampling and BMI. Here, samples showing small changes < 0.5ng/ml in either direction were omitted to avoid natural stoichiometric variation around zero. The relationship between change in BPA (ΔBPA) and

For peer review only

BPA risk score was assessed by linear regression, adjusted for sex, time of sampling and BMI both with and without adjustment for creatinine. Statistical analysis was carried out using SPSS, v.22 (IBM, USA).

Impact of following reduced BPA diet on lifestyle

We carried out quantitative and qualitative analysis to address long-term sustainability of the diet. Data on the impact of following the diet on feelings of dietary restriction, time spent sourcing or preparing meals, calorific intake and long term sustainability were collected via a questionnaire (See Supplementary information file 4). The questionnaire also included a freeform section where participants could write about their experiences following the diet in a non-prescribed fashion for qualitative analysis. Qualitative data was assessed for thematic content by two experienced qualitative researchers. Key themes were independently identified and coded until agreement was reached.

RESULTS

Participant Characteristics

There were 104 volunteer participants in this engaged research study. A total of 104 students participated in the intervention, but a small number were absent or unable to produce a urine sample at both visits. A complete dataset was received from 94 students. Information on the characteristics of the study cohort are given in table 1.

For peer review only

Table 1: Characteristics of the study population. A complete dataset was available on 94 out of 104 participants. IQR = interquartile range, SD = Standard deviation. The units of BPA are ng/ml and BMI is defined as Kg/m^2. LoQ = limit of quantification. Urinary BPA levels are given both as unadjusted data and as a BPA (ng/ml) to creatinine (mg/ml) ratio.

Unadjusted urinary BPA at visit 1 (n = 98)	
median (IQR)	1.37 (2.52)
95% confidence intervals	1.58 to 2.57
mean (SD)	2.07 (2.51)
Number of samples below LoQ (0.1ng/ml)	16
Minimum value (ng/ml)	0.07
Maximum value (ng/ml)	13.55
Creatinine-adjusted urinary BPA at visit 1 (n = 98)	
median (IQR)	1.34 (1.82)
95% confidence intervals	1.38 to 2.13
mean (SD)	1.75 (1.82)
Number of samples below LoQ (0.1ng/ml)	16
Minimum value (ng/ml)	0.05
Maximum value (ng/ml)	9.52
Unadjusted urinary BPA at visit 2 (n = 99)	
median (IQR)	1.91 (2.68)
95% confidence intervals	2.15 to 4.56
mean (SD)	3.35 (6.18)
Number of samples below LoQ (0.1ng/ml)	12
Minimum value (ng/ml)	0.07
Maximum value (ng/ml)	49.6
Creatinine-adjusted urinary BPA at visit 2 (n = 99)	
median (IQR)	1.31 (2.24)
95% confidence intervals	1.46 to 8.34
mean (SD)	4.90 (16.8)
Number of samples below LoQ (0.1ng/ml)	12
Minimum value (ng/ml)	0.04
Maximum value (ng/ml)	139.33
Unadjusted ΔBPA (n = 94)	
median (IQR)	0.14
95% confidence intervals	0.15 to 2.41
mean (SD)	1.28 (5.79)
Minimum value	-8.02
Maximum value	49.5
Adjusted ΔBPA (n = 94)	
median (IQR)	0.02 (2.61)
95% confidence intervals	-0.23 to 6.53
mean (SD)	3.15 (16.5)
Minimum value	-8.6

For peer review only

Maximum value	133.45
BPA risk score (n = 99)	
median (IQR)	15.0 (10.3)
95% confidence intervals	15.5 to 18.4
mean (SD)	17.0 (7.12)
Demographics (n= 99)	
Sex - % male	44
Exposure to estrogens - % of cohort	14
BMI– median (IQR)	20.7 (3.45)
BMI– mean (SD)	21.2 (3.07)

BPA was detected in the urine of 86% of subjects at visit 1 prior to the intervention. Missing samples were due to non-attendance of participants or non-provision of a suitable sample. Samples below the limit of quantification were scored as 0.07 ng/ml (LoQ/√2).

Creatinine-adjusted urinary BPA concentrations do not change significantly after following an intervention diet designed to reduce BPA exposure for 7 days.

The median change in creatinine-adjusted urinary BPA between visits (ΔBPA) was 0.02 ng/ml with an interquartile range of 2.61 ng/ml. We identified no changes in urinary BPA between visits ($p = 0.25$; figure 1a). 3 outliers with very high urinary BPA readings at visit 2 were excluded from the analysis, since these samples lay outside the linear range of analysis, so confidence in quantification was poor. No confounding factors included in the analysis were associated with change in BPA ($p = 0.78$, 0.43 and 0.36 for sex, time of sample collection and BMI respectively). We also identified no change in BPA levels between visits using data uncorrected for creatinine ($p = 0.20$). We also assessed whether participants from different schools showed variable BPA levels at either visit 1, or change in BPA, but no such effects were noted.

For peer review only

Similarly, no relationship between change in urinary BPA (ΔBPA) and BPA risk score was identified (beta coefficient 0.08, standard error 0.07, p = 0.55; figure 1b). No associations were noted between change in urinary BPA and BPA risk score in data not adjusted for creatinine (p = 0.27). We found no association between ΔBPA and BPA risk score when considering only the exposure on the day prior to testing, taking into account the short half-life of BPA (p = 0.16 and p = 0.33 for adjusted and unadjusted data respectively).

Participants with highest starting urinary BPA levels were more likely to demonstrate lower BPA levels at visit 2.

We found an inverse relationship between initial BPA levels and whether a participant had reduced BPA levels at visit 2 (p = 0.003). These data indicate that the participants in the cohort with the highest creatinine-adjusted urinary BPA levels at visit 1 were more likely to demonstrate a drop in their urinary BPA at visit 2 (figure 2).

Following the intervention diet has significant effects on participant lifestyle

Participants indicated that following the diet had no significant cost implications on family finances, with 50% of participants reporting that it had cost more, and 50% reporting that costs had decreased or remained the same. Although participants did not spend longer preparing their food, 78% of participants reported that their shopping took longer. 58% of participants reported that the diet did not affect their calorific intake. 91% of participants reported that they felt at least slightly restricted in their food choices. 27% of participants

For peer review only

reported that they felt very restricted. Finally, 66% of participants stated that they would find it hard or very hard to follow the diet long term.

Qualitative analysis of the effect of following the diet on lifestyle

We identified 5 overriding themes in our qualitative analysis of the effect of following the diet on lifestyle. These were 1) the widespread use of plastics possibly containing BPA in food packaging ("almost everything is packaged in plastic" – participant 70, "Literally everything involved plastic" – participant 28). 2) Lack of clarity in labelling of products and packaging potentially containing BPA ("I found it really hard to know what foods I could eat … there is never a guarantee it is BPA free" – participant 43, " The biggest problem was that a lot of packaging doesn't state what type of plastic it is or whether it contains BPA" – participant 74). 3) The perceived restrictions of being on the 'real world' BPA avoidance diet ("Difficulty eating out, hard to find foods in college or 'out' that hadn't touched BPA. My family had a takeaway on Saturday night and I couldn't eat it" – participant 56, "Sometimes I can't eat / drink what I want because of the recycling number" – participant 112). 4) The impact of eating 'BPA free' was the only positive theme emerging ("I feel I have eaten much more healthily this week … I didn't eat so much junk food" – participant 74, "I ate more vegetables and less chocolate" – participant 83). 5) The impact on shopping habits ("You can't get it all from supermarkets" – Participant 37; "Had to go to more individual food shops" – participant 103).

DISCUSSION

For peer review only

Exposure to the endocrine disrupting chemical Bisphenol A (BPA) is ubiquitous [2], with growing evidence that it may be associated with adverse health outcomes [4]. Here, 104 researcher participants aged 17-19 years designed and undertook a quantitative and qualitative engaged research project designed to assess the potential for reduction of personal exposure to BPA through moderation of diet, which would have utility in a 'real world' setting. We conclude that the 'real world' diet designed to reduce BPA exposure had no effect on creatinine-adjusted urinary BPA concentrations in our cohort over a period of 7 days in our dataset.

Although levels of urinary BPA in our study cohort were slightly lower at the outset of the study in our cohort than in others [18], measureable levels were present in the vast majority of our participants. Participants were unable to achieve a reduction in their urinary BPA levels over the 7 day trial period, despite good compliance to supplied guidelines. Avoidance of BPA was not easily achieved on an individual level in our study population, with qualitative analysis indicating that participants experienced feelings of restriction and difficulties in sourcing BPA-free food due to inadequate labelling of foods and food packaging. This suggests that the intervention would be difficult to sustain in the longer term.

This work represents the largest group of unrelated participants in one of the highest exposure demographics to date, since previous work has focused on families and related individuals [16] [17], who may share common sources of BPA. Although other population demographics such as young children may have higher levels of BPA than our chosen study population [18], it would not have been possible to do the sort of engaged research project that we envisaged in this group. Our intervention is a 'real world' diet, designed to a set of guidelines

For peer review only

(such as reduction in the usage of tinned foods or foods with high levels of processing), rather than the strict, prescribed diets that have been used in other studies [16], which suggested that it was possible for participants to reduce their urinary BPA excretion by approximately 60% in a period of just 3 days [16]. In our self-designed, self-administered study this was unachievable. This may reflect the difficulty in identifying and sourcing foods free of BPA in our commercial environment. Finally, the qualitative thematic analysis we carried out in our study has given an indication that adherence to even a 'real world' BPA reduction diet with fewer restrictions and more choice over the longer term was unlikely in our study population due to difficulties in identifying foodstuffs likely to contain less BPA.

BPA has a terminal half-life of 6 hours [15]. Spot samples may therefore not be as accurate as continuous sampling strategies (24hr urine collection). However, recent studies suggest that despite its short half-life, measureable BPA remains present for up to 43 hours post-fasting, indicating non-food exposures or accumulation in body tissues such as fat [20]. We identified no impact of time of sample collection on BPA levels in our sample set, in either creatinine-adjusted or unadjusted data, indicating that our measurements were not influenced by time since the last meal. Spot sampling as used here may therefore represent an acceptable compromise and remains a practical option in the community setting of our study. The large variability in urinary BPA levels within an individual sampled at different times may also have reduced our ability to observe an effect. This could be facilitated by the use of multiple sampling, or pools of multiple urines, but was not feasible within the confines of our study.

Calculating an accurate BPA risk score is challenging. Data were self-reported, and foodstuffs are not labelled for BPA content. It is difficult to generalise across food types and

For peer review only

large variations in BPA concentrations occur between different products of the same food type or even different lots of the same product [1]. Foods that were free of BPA-containing packaging (as far as it was possible to tell) may have been highly processed or contain food items from a variety of sources. Highly processed and 'fast' food has previously been demonstrated to be a source of BPA [21]. A study of the temporal trends seen in composite food samples found no change in the overall BPA content of the food, despite large reduction in the BPA content of some individual food items, illustrating the difficulties in effectively excluding BPA from a varied diet [22]. Participants may therefore have changed BPA containing foods for other, perceived healthier choices, which may still contain BPA by virtue of processing.

BPA enters foodstuffs by leaching from polycarbonate or epoxy resin after manufacture, or by hydrolysis of the polymer itself [23]. The migration rate of BPA increases with higher temperatures [24], and with time and use, e.g. repeated use of polycarbonate water bottles [25]. Exposure to BPA can also occur through routes other than food, including dust ingestion and dermal absorption [26] and this was not taken into account in our study. A study of volunteers who purposefully handled thermal receipts showed an increase in urinary BPA excretion of up to 84%, and their BPA levels took longer to return to pre exposure levels, suggesting a difference in the bio-availability of BPA through skin and oral routes [27]. It is also possible that some manufacturers may have voluntarily reduced the amount of BPA-containing food packaging compared to their previous usage, given the attention that endocrine disrupting chemicals have received in the media. However, measurable levels of BPA were still detected in the majority of participants in our study, which suggests that there may be other, non-dietary, sources of BPA, and that exposure to BPA remains an issue. We may also have been underpowered to detect subtle changes in urinary BPA, given the

For peer review only

heterogeneity in food choice; detection of such effects may need thousands of participants. Finally, our study, like other studies of its type, does not take account of inter-individual differences in the metabolism and excretion of BPA arising from differences in genetic background between people. BPA is metabolised primarily by UDP-glucuronosyltransferases, and altered activity polymorphisms of these enzymes have been reported [28].

Emerging evidence suggests that that BPA may be linked to several chronic human health conditions [6-9, 29], suggesting that continued study of the human health effects of BPA exposure is justified. The opinion of the European Food Safety Authority (EFSA), is that whilst uncertainty over the human health effects of BPA exists, caution should be exercised in ingestion of BPA [3]. Our data suggests that in our study population, it is unlikely that participants could moderate their own BPA exposure in the long term by self-directed modification of diet in a 'real world' setting, and furthermore, participants would have been reluctant to adopt such a lifestyle change in the longer term due to the restrictions in dietary choice and the effects on day to day life. Most of these barriers appear to arise from the pervasiveness of BPA in our food chain, and inadequate labelling of foods packaged in BPA-containing substances. We propose that until a definitive assessment of the health risks of BPA is available, informed choice over whether or not to consume BPA and similar chemicals in foodstuffs should be facilitated by better labelling.

CONTRIBUTORSHIP STATEMENT

TSG - Contributed to study design and co-wrote the paper

For peer review only

NB - Contributed to study design and participant involvement

BP - Managed the technical aspects of the project and reviewed the manuscript

ALC - Contributed to data entry and interpretation and reviewed the manuscript

BPA Schools Study Consortium members - designed and interpreted the study and contributed to the manuscript.

MHS - Carried out the qualitative analysis and reviewed the manuscript

AMS - Managed sample collection, contributed to study design and reviewed the manuscript.

LWH - PI , managed the study, wrote the manuscript

ACKNOWLEDGEMENTS

We acknowledge the work of the NIHR Exeter Clinical Research Facility in aiding the collection of the urine samples.

COMPETING INTERESTS

The authors have no competing interests to declare.

DATA SHARING STATEMENT

Extra data is available upon reasonable request by emailing Lorna Harries (L.W.Harries@exeter.ac.uk).

For peer review only

FUNDING

This study was funded by a Wellcome Trust People Award to LWH and TG (grant number 105162/Z/14/Z).

REFERENCES

1 Geens T, Aerts D, Berthot C, *et al.* A review of dietary and non-dietary exposure to bisphenol-A. *Food Chem Toxicol* 2012;**50**:3725-40.

2 WHO. World Health Organisation Background paper on mechanisms of action of bisphenol A and other biochemical/molecular interactions. *WHO/HSE/FOS* 2010;**11.1**.

3 EFSA. Scientific opinion on the risks to public health related to the presence of bisphenol A (BPA) in foodstuffs. *EFSA journal* 2015;**13**.

4 Rochester JR. Bisphenol A and human health: a review of the literature. *Reprod Toxicol* 2013;**42**:132-55.

5 WHO. Joint FAO/WHO Expert meeting to review toxicological and health aspects of bisphenol A Summary report. 2010.

6 Galloway T, Cipelli R, Guralnik J, *et al.* Daily bisphenol A excretion and associations with sex hormone concentrations: results from the InCHIANTI adult population study. *Environ Health Perspect* 2010;**118**:1603-8.

For peer review only

7 Melzer D, Rice NE, Lewis C*, et al.* Association of urinary bisphenol a concentration with heart disease: evidence from NHANES 2003/06. *PLoS One* 2010;**5**:e8673.

8 Song Y, Chou EL, Baecker A*, et al.* Endocrine-disrupting chemicals, risk of type 2 diabetes, and diabetes-related metabolic traits: A systematic review and meta-analysis. *J Diabetes* 2015.

9 Savastano S, Tarantino G, D'Esposito V*, et al.* Bisphenol-A plasma levels are related to inflammatory markers, visceral obesity and insulin-resistance: a cross-sectional study on adult male population. *J Transl Med* 2015;**13**:169.

10 Braun JM. Early-life exposure to EDCs: role in childhood obesity and neurodevelopment. *Nat Rev Endocrinol* 2017;**13**:161-73.

11 Vandenberg LN, Chahoud I, Heindel JJ*, et al.* Urinary, circulating, and tissue biomonitoring studies indicate widespread exposure to bisphenol A. *Environ Health Perspect* 2010;**118**:1055-70.

12 Gore AC, Chappell VA, Fenton SE*, et al.* EDC-2: The Endocrine Society's Second Scientific Statement on Endocrine-Disrupting Chemicals. *Endocr Rev* 2015;**36**:E1-E150.

13 Agency EC. AGREEMENT OF THE MEMBER STATE COMMITTEE ON THE IDENTIFICATION OF 4,4'-ISOPROPYLIDENEDIPHENOL (BISPHENOL A) AS A SUBSTANCE OF VERY HIGH CONCERN 2017.

14 Lakind JS, Naiman DQ. Daily intake of bisphenol A and potential sources of exposure: 2005-2006 National Health and Nutrition Examination Survey. *Journal of exposure science & environmental epidemiology* 2011;**21**:272-9.

15 Dekant W, Volkel W. Human exposure to bisphenol A by biomonitoring: methods, results and assessment of environmental exposures. *Toxicol Appl Pharmacol* 2008;**228**:114-34.

For peer review only

16 Rudel RA, Gray JM, Engel CL, *et al.* Food packaging and bisphenol A and bis(2-ethyhexyl) phthalate exposure: findings from a dietary intervention. *Environ Health Perspect* 2011;**119**:914-20.

17 Sathyanarayana S, Alcedo G, Saelens BE, *et al.* Unexpected results in a randomized dietary trial to reduce phthalate and bisphenol A exposures. *Journal of exposure science & environmental epidemiology* 2013;**23**:378-84.

18 Calafat AM, Ye X, Wong LY, *et al.* Exposure of the U.S. population to bisphenol A and 4-tertiary-octylphenol: 2003-2004. *Environ Health Perspect* 2008;**116**:39-44.

19 Hornung R, Reed L. Estimation of average concentration in the presence of nondetectable values. *Appl Occupat Environ Hyg* 1990;**5**:46–51.

20 Stahlhut RW, Welshons WV, Swan SH. Bisphenol A data in NHANES suggest longer than expected half-life, substantial nonfood exposure, or both. *Environ Health Perspect* 2009;**117**:784-9.

21 Zota AR, Phillips CA, Mitro SD. Recent Fast Food Consumption and Bisphenol A and Phthalates Exposures among the U.S. Population in NHANES, 2003-2010. *Environ Health Perspect* 2016;**124**:1521-8.

22 Cao XL, Perez-Locas C, Robichaud A, *et al.* Levels and temporal trend of bisphenol A in composite food samples from Canadian Total Diet Study 2008-2012. *Food Addit Contam Part A Chem Anal Control Expo Risk Assess* 2015;**32**:2154-60.

23 Aschberger K, Castello P, Hoekstra E, *et al.* Bisphenol A and baby bottles: challenges and perspectives. 2010.

For peer review only

24 Le HH, Carlson EM, Chua JP, *et al.* Bisphenol A is released from polycarbonate drinking bottles and mimics the neurotoxic actions of estrogen in developing cerebellar neurons. *Toxicol Lett* 2008;**176**:149-56.

25 Brede C, Fjeldal P, Skjevrak I, *et al.* Increased migration levels of bisphenol A from polycarbonate baby bottles after dishwashing, boiling and brushing. *Food Addit Contam* 2003;**20**:684-9.

26 Myridakis A, Chalkiadaki G, Fotou M, *et al.* Exposure of Preschool-Age Greek Children (RHEA Cohort) to Bisphenol A, Parabens, Phthalates, and Organophosphates. *Environ Sci Technol* 2016;**50**:932-41.

27 Lv Y, Lu S, Dai Y, *et al.* Higher dermal exposure of cashiers to BPA and its association with DNA oxidative damage. *Environ Int* 2017;**98**:69-74.

28 Stingl JC, Bartels H, Viviani R, *et al.* Relevance of UDP-glucuronosyltransferase polymorphisms for drug dosing: A quantitative systematic review. *Pharmacol Ther* 2014;**141**:92-116.

29 Melzer D, Osborne NJ, Henley WE, *et al.* Urinary bisphenol A concentration and risk of future coronary artery disease in apparently healthy men and women. *Circulation* 2012;**125**:1482-90.

For peer review only

FIGURE LEGENDS

<u>Figure 1. The effect of a 'real world' BPA avoidance diet on urinary BPA exposure over a 7 day period</u>. **A**. Urinary BPA levels (ng/ml) adjusted for urinary creatinine were plotted at visit 1 before the intervention and at visit 2 after the intervention. The 3 extreme outliers have been removed. The trajectories of individual participant measurements are shown. **B**. Change in urinary BPA levels in ng/ml following the intervention diet are plotted against the self-reported BPA risk score.

<u>Figure 2. The effect of baseline urinary BPA on the probability of achieving a drop in levels following the intervention</u>. This graph illustrates the median urinary BPA level adjusted for creatinine at visit 1 prior to the intervention expressed relative to whether or not a reduction in urinary BPA levels was achieved following the 7 day intervention diet at visit 2. Error bars refer to the interquartile range of measurement.

For peer review only

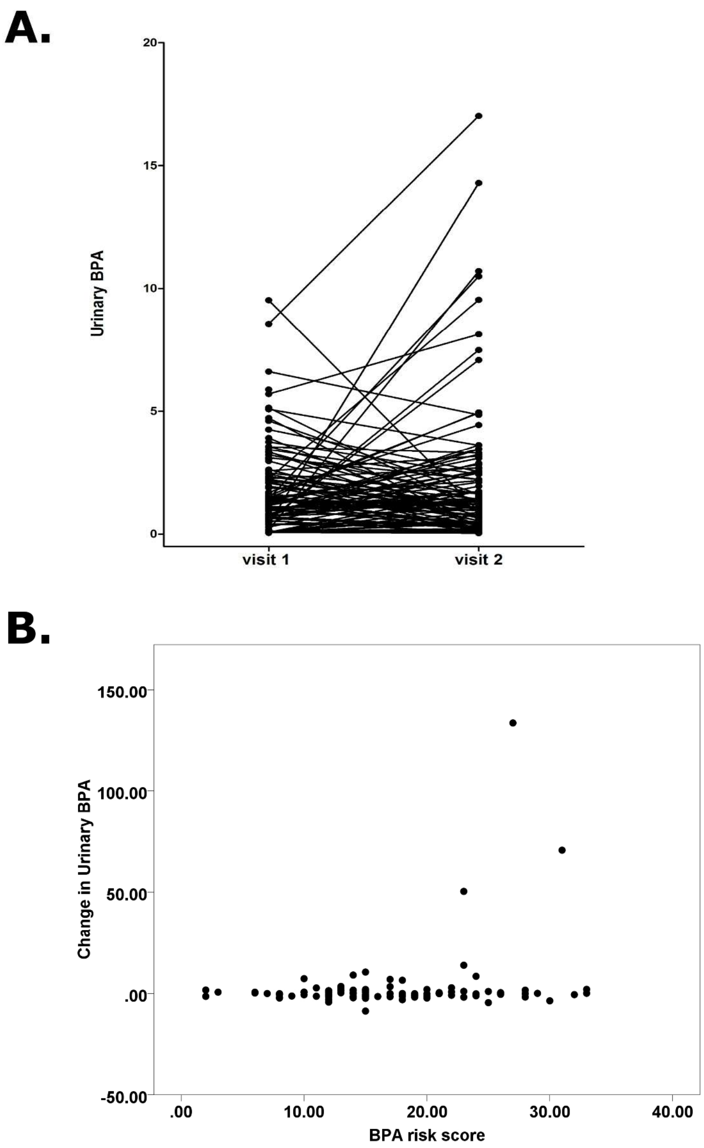

Figure 1

209x297mm (300 x 300 DPI)

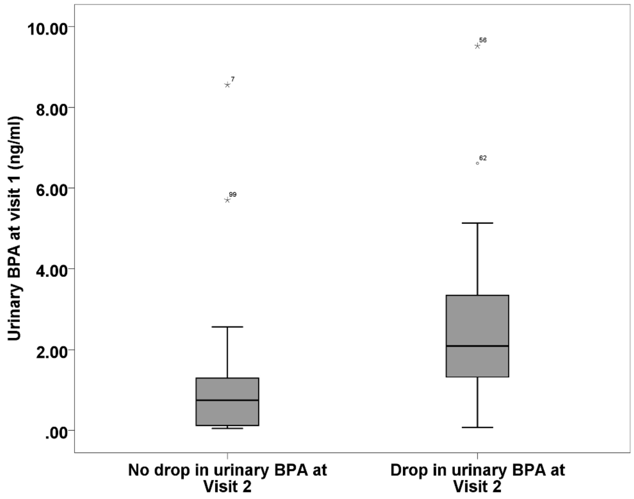

Figure 2

209x297mm (300 x 300 DPI)

Supplementary information file 1. Members of the BPA Schools study consortium

Surname	Initials	Forename	Affiliation
Mark		Hill	Clyst Vale Community College
Jasmine	R	Ayres	Clyst Vale Community College
Thomas		Blake	Clyst Vale Community College
Max		Branson	Clyst Vale Community College
Abigail	S	Bunkum	Clyst Vale Community College
Wai Chon		Cheong	Clyst Vale Community College
Lauren	J	Evans	Clyst Vale Community College
Amalia	R	Gimbuta	Clyst Vale Community College
Toby	I	Goodwin	Clyst Vale Community College
Robin	P	Hannaford	Clyst Vale Community College
Luke	J	Harris	Clyst Vale Community College
Ferne	R	Kelly	Clyst Vale Community College
Shannon	T	Reiness	Clyst Vale Community College
Eleanor	J	Self	Clyst Vale Community College
Katherine	O	Stagg	Clyst Vale Community College
Peter	JC	Boddington	Exeter School
Andrew	B	Brew	Exeter School
George	W	Hoult	Exeter School
Suraj	M	Gandhi	Exeter School
Joshua	G	Hanna	Exeter School
Harry	FZ	Hart	Exeter School
Thomas	RW	Hart	Exeter School
Harriet	M	Jenner	Exeter School
Annabel	D	Ling	Exeter School
Elspeth	O	Mabin	Exeter School
Abigail	M	Pelling	Exeter School
Rebecca	C	Richardson	Exeter School
Hannah	V	Withers	Exeter School
Nicholas	W	Long	South Dartmoor Community College
Megan	R	Abbott	South Dartmoor Community College
Alice	C	Crocker	South Dartmoor Community College
Merlin		Davies	South Dartmoor Community College
Rebecca	L	Evans	South Dartmoor Community College
Oliver	I	Mant	South Dartmoor Community College
Alexandra	C	McHugh	South Dartmoor Community College
Alex	J	Murray-Preece	South Dartmoor Community College
Louise	Y	Pearse	South Dartmoor Community College
Thomas	J	Roberts	South Dartmoor Community College
Molly		Sansom	South Dartmoor Community College
Eve	M I P	Scott	South Dartmoor Community College
Samuel	J	Tomkinson	South Dartmoor Community College
Ross		Spicer	South Dartmoor Community College
Mel	C	Parker	Honiton Community College
Ameerah	M	Askew	Honiton Community College
Kyle	T	Blackmore	Honiton Community College

For peer review only

Caitlin	L	Burnett	Honiton Community College
Isla	L	Cloke	Honiton Community College
Kira	J	Foster	Honiton Community College
Ella	L	George	Honiton Community College
Daniel	J	Hole	Honiton Community College
Lauren	J	Hunt	Honiton Community College
Jack		Lane	Honiton Community College
Callum	J	Nicholas	Honiton Community College
Ben	C	Willis	Honiton Community College
Lena		Marvolo	Exeter College
Richard	G	Lloyd	Exeter College
Riona		Adams	Exeter College
Zoe		Baker	Exeter College
Kai	S	Cartwright	Exeter College
Lucinda		Cauchois	Exeter College
Alana	L	Cram	Exeter College
Brianna	M	Dooley	Exeter College
Ella		Fermor-Kissane	Exeter College
Annick		Gielens	Exeter College
Jessica	M	Godfrey	Exeter College
Shauna		Green	Exeter College
Hesham	MH	Hanna	Exeter College
Lewis	E	Hare	Exeter College
Hannah	M	Hart	Exeter College
Freya	J	Hester	Exeter College
Nicola	LM	Hill	Exeter College
Honour		James	Exeter College
Jalil		Khalil	Exeter College
Callum		Larcombe	Exeter College
Zhengchao		Lu	Exeter College
Zoe		McCarthy	Exeter College
Qiaochu		Meng	Exeter College
Rhiannon	I	Morris	Exeter College
Son		Nguyen	Exeter College
Isobella		Perks	Exeter College
Bethan		Soanes	Exeter College
Elizabeth	L	Ward	Exeter College
Jade		Webb	Exeter College
Jemima		Western	Exeter College
Charley		Winter	Exeter College
Kerry	J	Burnham	Exeter Mathematics School
Denis		Akkavim	Exeter Mathematics School
Will	A	Bevington	Exeter Mathematics School
Luke	PJL	Bowden	Exeter Mathematics School
James	J	Cockayne	Exeter Mathematics School
Louis	GT	de Mendonça	Exeter Mathematics School
Harry	B	Donovan	Exeter Mathematics School
Alex	T	Drake	Exeter Mathematics School

For peer review only

Jack	T	Fulls	Exeter Mathematics School
Theo	HD	Greenslade	Exeter Mathematics School
Embla	F	Hocking	Exeter Mathematics School
David	A	Hooton	Exeter Mathematics School
Nat		Lea	Exeter Mathematics School
Bethany		Lee	Exeter Mathematics School
Patrick	J	Locke	Exeter Mathematics School
Sam	R	Marriott	Exeter Mathematics School
Alex	J	Pace	Exeter Mathematics School
Chris	G	Parsons	Exeter Mathematics School
Mike		Price	Exeter Mathematics School
Jon	M	Reeves	Exeter Mathematics School
Jake	J	Shiel	Exeter Mathematics School
Ethan	J	Teague	Exeter Mathematics School
Mike	D	Thornton	Exeter Mathematics School
Ruth	J	Trevelyan	Exeter Mathematics School
Donovan	P	Tripp	Exeter Mathematics School
Eleanor	M	Van Vogt	Exeter Mathematics School
Hugh	R	Willman	Exeter Mathematics School
Alex	RJF	Winter	Exeter Mathematics School
Natalie	A	Woods	Exeter Mathematics School
Jasmine	M	Wootten	Exeter Mathematics School

For peer review only

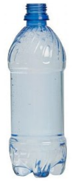

BPA: Myth and Reality
Dietary Intervention Guidelines

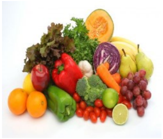

General instructions

The purpose of this dietary intervention trial is to follow a diet designed to minimise routes of exposure to the food packaging chemical bisphenol A (BPA). For the dietary intervention period, please follow as closely as possible the instruction given below. Try to maintain your diet during the intervention period to be as closely similar to your normal diet as possible, in terms of the content, amount and calorific value of the food you eat. Please record details of each meal and the drinks and snacks you consume on the forms provided. Below are some general cooking and eating tips and an indication of which foods are best to avoid and those that are considered a low source of BPA.

Cooking and eating tips for the intervention period.

The general approach is to replace any food items that fall into the 'avoidance' category with an alternative, chosen to minimise exposure to BPA

- **Switch to stainless steel and glass food storage and drink containers.**
- **Move foods to ceramic or glass food containers before microwaving.**
- **Consider a coffee filter or percolator for coffee – home coffee makers (Such as Nespresso™) may have polycarbonate-based water tanks and phthalate-based tubing.**
- **Eat out less, especially at restaurants that do not use fresh ingredients.**
- **Avoid canned food consumption. Where possible, replace with fresh produce or cardboard or tetrapack packaged alternatives.**
- **Choose fresh fruits and vegetables when possible, and frozen if not.**
- **Soak dried beans for cooking rather than tinned.**

For peer review only

Foods to avoid

Tinned foods. Top ten tinned foods that are reported to be sources of BPA include coconut milk, soup, meat, vegetables, meals (e.g. pasta with sauce), juice, fish, beans, meal replacement drinks, fruit.

Carbonated/fizzy drinks and juices in cans. Avoid carbonated drinks in cans and drinks stored for prolonged periods in reusable sports bottles, unless they are labelled 'BPA free' (many commercial sports bottles are).

Fast food from commercial outlets. Most processed food has passed through numerous processes, and each additional processing step provides an opportunity for BPA to enter through packaging or tubing. Try to replace fast and processed foods with a freshly prepared and cooked alternative.

Packaged fruit and vegetables. Replace these where possible with unpackaged, loose fruit and vegetable items as far as possible.

Convenience/ready meals. Plastics types considered safest in terms of chemical migration are recycling numbers 2 and 5. Avoid food prepared in packaging with recycling number 7, which includes many different types of polymer and mixed polymers, including polycarbonate, a source of BPA. Try to avoid foods that are designed to be heated in the microwave in their packaging.

Chocolate and ice cream. Individuals who report eating chocolate bars and ice cream on a regular basis have been reported to have higher than average BPA exposure. Try to avoid excessive consumption.

Non-food or food packaging routes of exposure

Although plastics found in consumer goods such as DVDs, CDs, computer goods and sunglasses do contain BPA, this is not an important route of exposure.

Till receipts often contain high levels of BPA, so wash your hands before eating or drinking if you have been handling them.

Dental sealants may contain BPA, so avoid any pre-planned dental work

Example daily diet

Food Item	Comments
**Breakfast**	
Cereal, Fruit	
Milk	Polypropylene or glass packaging
Bread	
Yoghurt	Choose polypropylene container
**Lunch**	
Meat or fish products	Check packaging and avoid those labelled no. 7. Avoid tinned ingredients
Cheese	
Salad items, Fruit	Choose unpackaged where possible, wash before use
Pasta	
**Dinner**	
Shepherds pie	Cooked in saucepan and oven rather than microwaved in plastic
Green beans	Fresh or frozen
Bread	
**Drinks**	
Water	Water direct from tap or use stainless steel or BPA free water bottle
Tea/coffee	Prepare in teapot or cafetiere, avoid commercial coffee makers
Carbonated drinks	Avoid canned drinks and those stored in reusable containers for prolonged periods
Milk	Polypropylene or glass packaging
**Snacks**	
Fruit	
Potato crisps	

For peer review only

Place participant barcode here	FOOD - DAY 1		DRINK – DAY 1	
**Contents of meal**	Was food packaged in plastic known or suspected to contain BPA?	Was food cooked in plastic known or suspected to contain BPA?	**Type of Drink**	Packaging (please describe) e.g. canned, plastic (Δ number), tetrapak
**Breakfast:**				
1.				
2.				
3.				
4.				
5.				
**Lunch**:				
1.				
2.				
3.				
4.				
5.				
**Dinner**:				
1.				
2.				
3.				
4.				
5.				
**Snacks**:				
1.				
2.				
3.				
4.				

For peer review only

Place participant barcode here	FOOD - DAY 2		DRINK – DAY 2	
**Contents of meal**	Was food packaged in plastic known or suspected to contain BPA?	Was food cooked in plastic known or suspected to contain BPA?	**Type of Drink**	Packaging (please describe) e.g. canned, plastic (Δ number), tetrapak
**Breakfast:**				
1.				
2.				
3.				
4.				
5.				
**Lunch**:				
1.				
2.				
3.				
4.				
5.				
**Dinner**:				
1.				
2.				
3.				
4.				
5.				
**Snacks**:				
1.				
2.				
3.				
4.				

For peer review only

Place participant barcode here	FOOD - DAY 3		DRINK – DAY 3	
**Contents of meal**	Was food packaged in plastic known or suspected to contain BPA?	Was food cooked in plastic known or suspected to contain BPA?	**Type of Drink**	Packaging (please describe) e.g. canned, plastic (Δ number), tetrapak
**Breakfast:**				
1.				
2.				
3.				
4.				
5.				
**Lunch**:				
1.				
2.				
3.				
4.				
5.				
**Dinner**:				
1.				
2.				
3.				
4.				
5.				
**Snacks**:				
1.				
2.				
3.				
4.				

For peer review only

Place participant barcode here	FOOD - DAY 4		DRINK – DAY 4	
**Contents of meal**	Was food packaged in plastic known or suspected to contain BPA?	Was food cooked in plastic known or suspected to contain BPA?	**Type of Drink**	Packaging (please describe) e.g. canned, plastic (Δ number), tetrapak
**Breakfast:**				
1.				
2.				
3.				
4.				
5.				
**Lunch**:				
1.				
2.				
3.				
4.				
5.				
**Dinner**:				
1.				
2.				
3.				
4.				
5.				
**Snacks**:				
1.				
2.				
3.				
4.				

For peer review only

Place participant barcode here	FOOD - DAY 5		DRINK – DAY 5	
**Contents of meal**	Was food packaged in plastic known or suspected to contain BPA?	Was food cooked in plastic known or suspected to contain BPA?	**Type of Drink**	Packaging (please describe) e.g. canned, plastic (Δ number), tetrapak
**Breakfast:**				
1.				
2.				
3.				
4.				
5.				
**Lunch**:				
1.				
2.				
3.				
4.				
5.				
**Dinner**:				
1.				
2.				
3.				
4.				
5.				
**Snacks**:				
1.				
2.				
3.				
4.				

For peer review only

Place participant barcode here	FOOD - DAY 6		DRINK – DAY 6	
**Contents of meal**	Was food packaged in plastic known or suspected to contain BPA?	Was food cooked in plastic known or suspected to contain BPA?	**Type of Drink**	Packaging (please describe) e.g. canned, plastic (Δ number), tetrapak
**Breakfast:**				
1.				
2.				
3.				
4.				
5.				
**Lunch**:				
1.				
2.				
3.				
4.				
5.				
**Dinner**:				
1.				
2.				
3.				
4.				
5.				
**Snacks**:				
1.				
2.				
3.				
4.				

For peer review only

Place participant barcode here	FOOD - DAY 7		DRINK – DAY 7	
**Contents of meal**	Was food packaged in plastic known or suspected to contain BPA?	Was food cooked in plastic known or suspected to contain BPA?	**Type of Drink**	Packaging (please describe) e.g. canned, plastic (Δ number), tetrapak
**Breakfast:**				
1.				
2.				
3.				
4.				
5.				
**Lunch**:				
1.				
2.				
3.				
4.				
5.				
**Dinner**:				
1.				
2.				
3.				
4.				
5.				
**Snacks**:				
1.				
2.				
3.				
4.				

Participant barcode

Additional study information.

Please do not feel obliged to answer these questions if you are uncomfortable doing so.

Gender

☐ Female
☐ Male
☐ Prefer not to say

Tobacco Usage – Have you used tobacco over the past week

☐ Yes
If so, what type and how much?____
☐ No
☐ Prefer not to say

Alcohol Usage – Have you used alcohol over the past week

☐ Yes
If so, what type and how much?____
☐ No
☐ Prefer not to say

Medication- Have you taken any medication over the last week?

☐ Yes
☐ No
☐ Prefer not to say

If so, Please name the medication____________________ ☐ Prefer not to say

Vegetarian/vegan diet - Have you eaten or drank any soya products over the past week?

☐ Yes
☐ No
☐ Prefer not to say

Your measurements - leave blank if you prefer not to say

Your height _________________________

Your weight_________________________

For peer review only

Participant Barcode

BPA: Myth and Reality diet questionnaire

1. Were there any times during the week that you knowingly/unknowingly did not stick to the diet? Please tick any that apply and give indication of frequency.

School meals ☐ _____times

Restaurants/cafés ☐ _____times

Friends' houses ☐ _____times

Takeaway ☐ _____times

Other ______________________________ ☐ _____times

2. If you heated your food in a microwave, what was the food in? Tick any which apply and give indication of frequency.

A food storage container or bowl known or suspected to contain BPA ☐_____times

3. When you or your family drank water, where did your water come from? Tick any which apply and give indication of frequency.

Plastic filter jug known or suspected to contain BPA ☐_____times

Individual water bottle known or suspected to contain BPA ☐_____times

Larger water container known or suspected to contain BPA ☐_____times

4. How many times during the week did you eat food that had been stored or transported in plastic containers known or suspected to contain BPA?

____

5. How many times during the week did you eat tinned food or drink from cans?

____

6. Did the BPA reduced diet affect How much you spent on shopping?

Spent more ☐

Spent less ☐

No difference ☐

Participant questionnaire V3 25Jun15

For peer review only

Participant Barcode

7. Did it take longer to source your food than usual?

Yes ☐ No ☐

If so, why? ______________________________

____

8. Did it take longer to prepare food than usual?

Yes ☐ No ☐

If so, why? ______________________________

____

9. How restricted did you feel by your food choice?

☐ Very ☐ Slightly ☐ No difference

If you felt you were restricted by the diet, why was this? ______________________________

____

10. Did the diet affect your calorific intake?

Yes ☐ No ☐

If so, why? ______________________________

____

For peer review only

Participant questionnaire V3 25Jun15

For peer review only - http://bmjopen.bmj.com/site/about/guidelines.xhtml

Participant Barcode

11. How easy would you find it to sustain this diet over a longer period of time?

Very easy ☐

Easy ☐

Hard ☐

Very hard ☐

Not sure ☐

12. Is there anything else about following the diet that you would like to add?

For peer review only - http://bmjopen.bmj.com/site/about/guidelines.xhtml

Thank you for reading this leaflet. If you wish to participate in this study, you will be asked to agree to the consent statements below in the presence of a member of the research team.

CONSENT STATEMENTS

1. I confirm that I have read this information sheet and have discussed participation in this project with my family. I have had opportunity to consider the information, ask questions and have had these questions answered satisfactorily. *You should not give consent until you are happy that you understand what the study involves.*

2. I understand that my participation is voluntary and that I am free to withdraw at any time without giving any reason, without my right to participate in the rest of the study being affected. *This means that even if you helped design this study you do not have to be a participant and you should not feel under any pressure to participate.*

3. I agree to participate in this study as a research subject. *This means that you agree to participate in a one-week diet and to provide two blood and urine samples.*

4. I understand that my anonymised blood and urine samples and linked anonymous questionnaire data will be sent to University of Exeter Medical School, Royal Devon & Exeter Hospital and my urine sample only will be sent to the Rolvaltain laboratory, a specialist BPA analysis company. *This means that laboratory staff will not know that samples belong to you, but dedicated staff at the University of Exeter, with training and experience in data protection ,will be able to link your sample data to your questionnaire data.*

5. I understand that RNA (genetic material) will be extracted from my blood and will be stored anonymously. *This means that Professor Harries' team will use our RNA to provide data that you will help analyse but may also do further research on the samples to identify reasons for any changes seen.*

6. I understand that data relating to my participation in the study will be returned anonymously to my school to be used for educational purposes. *This means that although you will get to analyse data from your samples there is no way you will know which data relates to your samples and which to other participants*.

Complaints:

If you have any complaints about the way in which this study has been carried out please contact the Chair of the University of Exeter Medical School Research Ethics Committee Peta Foxall PhD, Chair, UEMS Research Ethics Committee: P.J.D.Foxall@exeter.ac.uk.

This project has been reviewed and approved by the University of Exeter Medical School Research Ethics committee UEMS REC REFERENCE NUMBER: 15/07/074)

BPA: Myth or Reality?

A research study investigating the effect of chemicals in plastic on gene activity and whether dietary interventions can reduce BPA levels in teenagers.

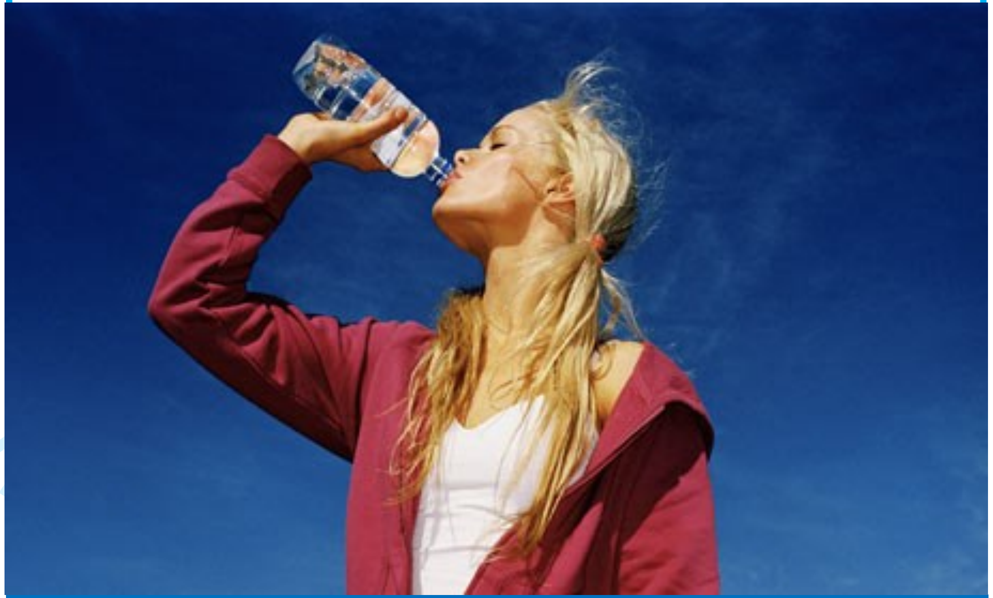

Involvement & Engagement

The aim of this year-long project is to involve teenagers in a research study that is relevant to them, by allowing them to help design a research project, analyse non-identifiable participant data and help to present and publish the outcomes.

Participation

Students will be asked to undertake a one week diet to reduce their intake of BPA, a chemical found in plastics. They will be asked to provide urine and blood samples before and after their diet.

For peer review only

For peer review only - http://bmjopen.bmj.com/site/about/guidelines.xhtml

What is BPA?

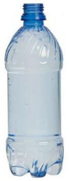

BPA (Bisphenol A) is a chemical used in the manufacture of plastics. Plastics containing BPA are found in a wide range of products including food and drink containers. BPA in these products can be ingested and there are concerns that high BPA levels in the blood could possibly affect human health. Research is therefore needed to understand its effects on the human body and how we can reduce its consumption by minor changes to our diet.

This project is being run as a student-involvement project to answer two specific questions:

1. **Can we see the effects of dietary BPA on our genes?**
2. **Can we effectively reduce BPA in our diet?**

In the past, small-scale experiments have shown that BPA levels in the human body can be reduced by rigid dietary interventions but these interventions would be difficult to implement in the “real world”. In this study a one-week dietary intervention designed by teenagers will be used by them to determine whether BPA levels, and the activity of BPA-responsive genes can be effectively reduced in young people by avoiding food packaging that contains this chemical.

What will I need to do?

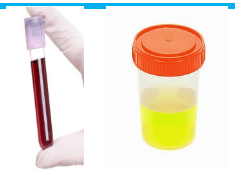

M

Day 1

- Provide a nurse with a 2.5ml blood sample and a urine sample.

Day 2 - Day 8

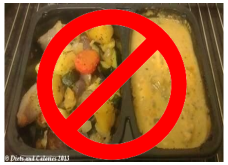

T W T

- Follow a diet that you have helped to design .
- The diet will exclude sources of BPA as much as possible but will be nutritionally and calorifically similar to your usual diet.
- You will be asked to complete a food diary and answer a questionnaire about how easy it was to follow this diet.

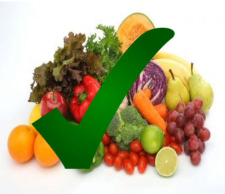

F S S

Day 8

- Provide a nurse with a 2.5ml blood sample and a urine sample.

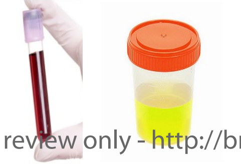

M

We recommend that you discuss the project with your family and involve them in planning what you eat and how you will prepare it.

What will happen to my samples and data?

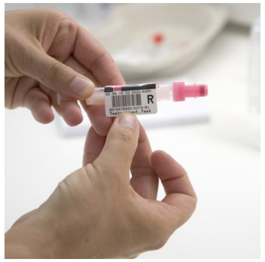

When you participate in the study you will be allocated with a numerical study ID. Your samples and data will be labelled with this number so that we can match your ‘before’ and ‘after’ diet samples with your food diary data. Once all data has been collated and coded it will be further anonymised by a person external to the project so that no data can be linked to any of the participants.

Urine samples from before and after the diet will be sent to the Royal Devon & Exeter NHS Foundation Trust for creatinine analysis and to the Rolvaltain laboratory for BPA analysis. RNA will be extracted from blood samples at the Royal Devon & Exeter Molecular Genetics Laboratory and the expression levels of two BPA-responsive genes will be measured in the samples taken before and after the diet. These anonymised RNA samples will be stored and used only by Professor Harries team for further research on the mechanisms behind our findings.

What are the benefits of taking part?

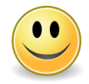

This project will help you to understand how you might be able to reduce BPA in your diet and your involvement in the design will give you an excellent insight into clinical research, community outreach and scientific practise. Your role as a participant is unlikely to have any direct health benefits.

Are there any risks in taking part?

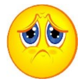

Blood samples will be taken by fully qualified and insured NHS personnel. Any potential discomfort or side-effects will be equivalent to that experienced giving a blood sample to your GP. All data will be fully anonymised before analysis. This means that you will not find out anything about your blood or urine samples. Following the diet may minimally increase the cost of your groceries for the week, but since fresh foods are usually less expensive than pre-packaged foods, we do not expect this to be an issue.

What will happen to the results of the research study?

You will be given the opportunity to help analyse anonymised data from this project and to help disseminate the outcomes of this research. It is hoped that the findings will be published in peer-reviewed journals and the wider media.

Who is organising this research?

The research is organised by Professors Lorna Harries & Tamara Galloway of the University of Exeter as part of their research program into BPA and part of the University’s outreach program to involve schools in academic research.

For peer review only - http://bmjopen.bmj.com/site/about/guidelines.xhtml

Version 4 (2/8/2015)

BPA: Myth & Reality

STUDY ID

CONSENT STATEMENTS	Please circle
1. I confirm that I have read information sheet BPA PIS Version 4 and have discussed participation in this project with my family. I have had opportunity to consider the information, ask questions and have had these questions answered satisfactorily. *You should not give consent until you are happy that you understand what the study involves.*	**YES / NO**
2. I understand that my participation is voluntary and that I am free to withdraw at any time without giving any reason, without right to participate in the rest of the study being affected. *This means that even if you helped design this study you do not have to be a participant and you should not feel under any pressure to participate.*	**YES / NO**
3. I agree to participate in this study as a research subject. *This means that you agree to participate in a one-week diet and to provide two blood and urine samples.*	**YES / NO**
4. I understand that my anonymised blood and urine samples and linked anonymous questionnaire data will be sent to University of Exeter Medical School, Royal Devon & Hospital and my urine sample only will be sent to the Rolvaltain laboratory, a specialist BPA analysis company. *This means that laboratory staff will not know that samples belong to you, but dedicated staff at the University of Exeter, with training and experience in data protection, will be able to link your sample data to your questionnaire data.*	**YES / NO**
5. I understand that RNA (genetic material) will be extracted from my blood and will be stored anonymously. *This means that Professor Harries' team will use our RNA to provide data that you will help analyse but may also do further research on the samples to identify reasons for any changes seen.*	**YES / NO**
6. I understand that data relating to my participation in the study will be returned anonymously to my school to be used for educational purposes. *This means that although you will get to analyse data from your samples there is no way you will know which data relates to your samples and which to other participants*.	**YES / NO**

Name of Participant	Signature	Date
Name of Person Obtaining Consent	Signature	Date

I the above signed testify the participant is providing voluntary and fully informed consent to participate in this study. I am on the delegation log to obtain consent for this study and are trained in obtaining consent.

This project has been reviewed and approved by the University of Exeter Medical School Research Ethics Committee UEMS REC REFERENCE NUMBER: 15/07/074)

For peer review only

Version 4 (2/8/2015)

BPA: Myth & Reality

For peer review only

STROBE Statement—checklist of items that should be included in reports of observational studies

	Item No	Recommendation
**Title and abstract**	1	(*a*) Indicate the study's design with a commonly used term in the title or the abstract
		(*b*) Provide in the abstract an informative and balanced summary of what was done and what was found<br><br>**a) Page 1**<br>**b) Page 2**
**Introduction**		
Background/rationale	2	Explain the scientific background and rationale for the investigation being reported<br><br>**Page 6, line 82**
Objectives	3	State specific objectives, including any prespecified hypotheses<br><br>**Page 7, line 113**
**Methods**		
Study design	4	Present key elements of study design early in the paper<br><br>**Page 8, line 140**
Setting	5	Describe the setting, locations, and relevant dates, including periods of recruitment, exposure, follow-up, and data collection<br><br>**Page 8, line 152**
Participants	6	(*a*) *Cohort study*—Give the eligibility criteria, and the sources and methods of selection of participants. Describe methods of follow-up<br><br>**Page 7, line 123**<br><br>*Case-control study*—Give the eligibility criteria, and the sources and methods of case ascertainment and control selection. Give the rationale for the choice of cases and controls<br>*Cross-sectional study*—Give the eligibility criteria, and the sources and methods of selection of participants
		(*b*) *Cohort study*—For matched studies, give matching criteria and number of exposed and unexposed<br>*Case-control study*—For matched studies, give matching criteria and the number of controls per case
Variables	7	Clearly define all outcomes, exposures, predictors, potential confounders, and effect modifiers. Give diagnostic criteria, if applicable<br><br>Page 7, line 140, Page 8, line 152.

For peer review only

Data sources/ measurement	8*	For each variable of interest, give sources of data and details of methods of assessment (measurement). Describe comparability of assessment methods if there is more than one group<br><br>**Page 8, line 140, Page 8, line 152**
Bias	9	Describe any efforts to address potential sources of bias<br><br>**Page 8, line 154, page 9 line 165**
Study size	10	Explain how the study size was arrived at<br><br>**Page 7, line 127**
Quantitative variables	11	Explain how quantitative variables were handled in the analyses. If applicable, describe which groupings were chosen and why<br><br>**Page 9, line 157**
Statistical methods	12	(*a*) Describe all statistical methods, including those used to control for confounding<br><br>**Page 9, line 170**
		(*b*) Describe any methods used to examine subgroups and interactions<br><br>**Page 9, line 175**
		(*c*) Explain how missing data were addressed<br><br>**Page 9, line 164**
		(*d*) *Cohort study*—If applicable, explain how loss to follow-up was addressed<br><br>**Page 12, line 206**<br><br>*Case-control study*—If applicable, explain how matching of cases and controls was addressed<br>*Cross-sectional study*—If applicable, describe analytical methods taking account of sampling strategy
		(*e*) Describe any sensitivity analyses<br><br>**N/A**

Continued on next page

For peer review only - http://bmjopen.bmj.com/site/about/guidelines.xhtml

For peer review only

**Results**		
Participants	13*	(a) Report numbers of individuals at each stage of study—eg numbers potentially eligible, examined for eligibility, confirmed eligible, included in the study, completing follow-up, and analysed<br><br>**Table 1**
		(b) Give reasons for non-participation at each stage<br><br>**Page 12, line 206**
		(c) Consider use of a flow diagram<br><br>**N/A**
Descriptive data	14*	(a) Give characteristics of study participants (eg demographic, clinical, social) and information on exposures and potential confounders<br><br>**Table 1**
		(b) Indicate number of participants with missing data for each variable of interest<br><br>**Table 1**
		(c) *Cohort study*—Summarise follow-up time (eg, average and total amount)<br><br>**Page 9, line 172**
Outcome data	15*	*Cohort study*—Report numbers of outcome events or summary measures over time<br><br>**Table 1**
		Case-control study—Report numbers in each exposure category, or summary measures of exposure
		Cross-sectional study—Report numbers of outcome events or summary measures
Main results	16	(*a*) Give unadjusted estimates and, if applicable, confounder-adjusted estimates and their precision (eg, 95% confidence interval). Make clear which confounders were adjusted for and why they were included<br><br>**Page 12, line 213 to page 13 line 229**
		(*b*) Report category boundaries when continuous variables were categorized<br><br>**Page 9, line 176**
		(*c*) If relevant, consider translating estimates of relative risk into absolute risk for a meaningful

For peer review only

		time period<br><br>**N/A**
Other analyses	17	Report other analyses done—eg analyses of subgroups and interactions, and sensitivity analyses<br><br>**Page 13, line 234**
**Discussion**		
Key results	18	Summarise key results with reference to study objectives<br><br>**Page 15, line 276**
Limitations	19	Discuss limitations of the study, taking into account sources of potential bias or imprecision. Discuss both direction and magnitude of any potential bias<br><br>**Page 16, line 298**
Interpretation	20	Give a cautious overall interpretation of results considering objectives, limitations, multiplicity of analyses, results from similar studies, and other relevant evidence<br><br>**Page 17, line 334**
Generalisability	21	Discuss the generalisability (external validity) of the study results<br><br>**Page 18, line 344**
**Other information**		
Funding	22	Give the source of funding and the role of the funders for the present study and, if applicable, for the original study on which the present article is based<br><br>**Page 19, line 374**

*Give information separately for cases and controls in case-control studies and, if applicable, for exposed and unexposed groups in cohort and cross-sectional studies.

Note: An Explanation and Elaboration article discusses each checklist item and gives methodological background and published examples of transparent reporting. The STROBE checklist is best used in conjunction with this article (freely available on the Web sites of PLoS Medicine at http://www.plosmedicine.org/, Annals of Internal Medicine at http://www.annals.org/, and Epidemiology at http://www.epidem.com/). Information on the STROBE Initiative is available at www.strobe-statement.org.

For peer review only

BMJ Open

An engaged research study to assess the effect of a 'real-world' dietary intervention on urinary bisphenol A (BPA) levels in teenagers

Journal:	*BMJ Open*
Manuscript ID	bmjopen-2017-018742.R2
Article Type:	Research
Date Submitted by the Author:	07-Nov-2017
Complete List of Authors:	Galloway, Tamara; University of Exeter, College of Life and Environmental Sciences<br>Baglin, Nigel; Research Projects<br>Lee, Benjamin; University of Exeter Medical School, Institute of Biomedical and Clinical Studies<br>Kocur, Anna; University of Exeter Medical School, Institute of Biomedical and Clinical Studies<br>Shepherd, Maggie; Royal Devon and Exeter National Health Service Foundation Trust, National Institute for Health Research Exeter Clinical Research Facility<br>Steele, Anna; Royal Devon and Exeter National Health Service Foundation Trust, National Institute for Health Research Exeter Clinical Research Facility<br>BPA Schools, Study Consortium; University of Exeter Medical School, Institute of Biomedical and Clinical Studies<br>Harries, Lorna; University of Exeter Medical School, Institute of Biomedical and Clinical Studies
**Primary Subject Heading**:	Public health
Secondary Subject Heading:	Communication
Keywords:	Bisphenol A, Dietary intervention, PUBLIC HEALTH, community, Engaged research

SCHOLARONE™
Manuscripts

An engaged research study to assess the effect of a 'real-world' dietary intervention on urinary bisphenol A (BPA) levels in teenagers

Tamara. S. Galloway[1], Nigel Baglin[2], Benjamin P Lee[3], A.L. Kocur[3], M.H. Shepherd[4], A.M. Steele[4], BPA schools study consortium[5,6,7,8,9,10] and L.W. Harries[3]

[1]*College of Life and Environmental Sciences, University of Exeter, Exeter UK EX4 4AS.*
[2]*Research Projects, Exeter, UK, EX2 5DQ.*
[3]*RNA-Mediated Disease Mechanisms group, Institute of Biomedical and Clinical Sciences, University of Exeter Medical School, University of Exeter, Exeter, UK EX2 5DW.*
[4]*National Institute for Health Research Exeter Clinical Research Facility, Royal Devon and Exeter National Health Service Foundation Trust, and University of Exeter Medical School, Exeter, U.K.*
[5]*Clyst Vale Community College, Broadclyst, Exeter Ex5 3AJ*
[6]*Exeter School, Exeter, Devon, EX2 4NS*
[7]*South Dartmoor Community College, Ashburton, Devon, TQ13 7EW*
[8]*Honiton Community College, Honiton, Devon, EX14 1QT*
[9]*Exeter College, Exeter, Devon, EX4 4JS*
[10]*Exeter Mathematics School, Exeter, Devon, EX4 3PU*

Word count 3021

Correspondence to:

Prof Lorna Harries
University of Exeter Medical School,
Barrack Road,
Exeter, EX2 5DW
Tel =44 1392 406773
Email: L.W.Harries@exeter.ac.uk

Declaration of competing issues

The authors have no competing interests to declare.

For peer review only

ABSTRACT

Objective

Bisphenol A has been associated adverse human health outcomes and exposure to this compound is near-ubiquitous in the Western world. We aimed to examine whether self-moderation of BPA exposure is possible by altering diet in a real-world setting.

Design

An Engaged Research dietary intervention study designed, implemented and analysed by healthy teenagers from 6 schools and undertaken in their own homes.

Participants

A total of 94 students aged between 17 and 19 years from schools in the South West of the UK provided diet diaries and urine samples for analysis.

Intervention

Researcher participants designed a set of literature-informed guidelines for reduction of dietary BPA to be followed for 7 days.

Main outcome measure

Creatinine-adjusted urinary BPA levels were taken before and after the intervention. Information on packaging and food/drink ingested was used to calculate a BPA risk score for anticipated exposure. A qualitative analysis was carried out to identify themes addressing long term sustainability of the diet.

Results

For peer review only

BPA was detected in urine of 86% of participants at baseline at a median value of 1.34 ng/ml (IQR 1.82). No effect of the intervention diet on BPA levels was identified overall (p = 0.25), but there was a positive association in those participants who showed a drop in urinary BPA concentration post intervention and their initial BPA level (p = 0.003). Qualitative analysis identified themes around feelings of lifestyle restriction and the inadequacy of current labelling practices.

Conclusions

We found no evidence in this self-administered intervention study that it was possible to moderate BPA exposure by diet in a real world setting. Furthermore, our study participants indicated that they would be unlikely to sustain such a diet long term, due to the difficulty in identifying BPA-free foods.

For peer review only

Article Summary

Strengths of the study

- This study represents the largest assessment to date of the potential for moderating one's own BPA exposure through diet
- The study was carried out in a 'real-world' setting rather than a regulated, controlled environment.
- The study was carried out in teenagers, the demographic with amongst the highest exposure.
- Qualitative analysis reveals challenges with sustaining such a diet.

Limitations of the study

- Calculation of a risk score is challenging due to the pervasive nature of BPA contamination

For peer review only

INTRODUCTION

Bisphenol A is one of the world's highest production volume chemicals. It is used in the manufacture of polycarbonate and other plastic consumer products, in heat resistant papers, dental sealants and in the epoxy resin-based lining of food and drink containers [1]. BPA can be found above the detection limit in the urine of the majority of people worldwide [2]. Concern has been raised for public health, since BPA is classified as an endocrine disrupting chemical (EDC) which has been linked with several disorders in cell and animal models [3-5]. Several epidemiological studies have also linked outcomes such as type 2 diabetes, cardiovascular disease, obesity and abnormalities of sex hormone levels with BPA levels in human populations [6-10] Epidemiological data in humans has historically been more contentious however, due to relatively small sample sizes and issues around causality [11]. The Endocrine Society concluded in 2015 that current evidence suggests that BPA and other endocrine disrupting chemicals may have effects on several reproductive, cardiovascular and metabolic traits in humans [12]. The current opinion of food regulatory bodies such as the European Food Standards Agency (EFSA) is that sufficient uncertainty remains to be able to exclude effects on the reproductive, immune, nervous, metabolic and cardiovascular systems and on cancer development [3] whilst the European Chemicals Agency (ECHA) has recently reclassified BPA as a chemical of very high concern due to its endocrine disrupting properties [13].

There has been wide interest in the sources of BPA and the potential for individuals to reduce their own exposure. Human exposure has been reported from inhalation of dust, uptake across the skin from thermal papers and till receipts and release from dental sealants. The main source is the ingestion of food and drink contaminated with BPA leached from

For peer review only

packaging materials [1, 14]. BPA is rapidly metabolised in the gut wall and liver and removed from the blood by the kidneys, with a terminal half-life of 6 hours after oral ingestion [15]. BPA has been detected in food samples packaged in glass, plastic, paper and paperboard cartons, with an average concentration of 0.46 ng/g, rising to over 700 ng/g for certain canned foods. Conversely, in a dietary intervention study in which 22 volunteers consumed a 3 day fresh food diet which excluded canned or packaged foods, there was a 66% reduction in urinary BPA excretion compared to concentrations before the intervention [16]. This latter study involved full dietary replacement of foodstuffs, an approach which is impractical for the population at large. A follow up study found that households who followed written recommendations produced by health care professionals showed no significant change in their BPA exposure [17].

We present an alternative, citizen-science based approach, where 108 student volunteers designed and undertook their own intervention diet, following provision of educational materials. We questioned whether adherence to a self-designed and self-administered ‘real world’ diet over 7 days would lead to significant reductions in excreted urinary BPA, and if so, whether such a diet was likely to be sustainable in the long term.

METHODS

Participant group

We chose adolescents because it has been shown that they have higher concentrations of BPA than adults (aggregated exposure of 1.449 μg/kg body weight per day) [3, 18]. A total of 108 students aged 17-19 from local schools were initially invited to participate in this engaged

For peer review only

research project. Six schools participated in this project (Clyst Vale Community College - 14 students; Exeter School - 12 students; South Dartmoor Community College - 13 students; Honiton Community College - 11 students; Exeter College – 29 students and Exeter Mathematics School – 29 students). Information and samples were available from 94 individuals at both visit one and visit 2 and comprise the complete dataset. This represents the largest intervention study in the population demographic with the one of the highest BPA exposures to date [18]. The number of students invited to participate was based on anticipated effect sizes from previous work of this nature [16], and we allowed for a 10% dropout rate. Students designed all of the materials required for completion of the study (study protocols, food diaries, lifestyle questionnaires, patient information sheets and consent forms (see Supplementary Information files 1 to 6).

Ethical Permission

Ethical permission was granted by the University of Exeter Medical School Ethics Committee (reference number 15/07/074) and the study was carried out in accordance with the Declaration of Helsinki.

The intervention diet

Students designed a “real world” diet designed to reduce consumption of BPA by avoidance of processed foods and foods packaged in known sources of BPA [1, 14]; supplementary information file 1). The study was designed at the University of Exeter as a collaboration

For peer review only

between academic staff and participating students and was developed at a series of interactive workshops attended by all parties.

Students were asked to minimise their intake of known sources of BPA according to a set of guidelines that had been co-designed with them based on the known literature. We requested that calorific intake was maintained as near to their usual diet as possible and recorded details of their daily diet including all food and drink, and its associated packaging, in a self-reported food diary (Supplementary information file 2). Adherence was assessed using a 'BPA risk score'; each individual dietary item potentially containing BPA was given a score of 1. Heavily processed items were also scored 1 per item. These scores were collated at the end of the 7 day trial to give a final risk score. An example of scores for a single participant on a single day is given in supplementary information file 3. Given the short half-life of BPA, we also carried out a secondary analysis considering only the BPA risk score from the 24 hours immediately preceding the second sample. Information on lifestyle factors including sex, BMI and time of urine collection was also collected (Supplementary information file 4). We recognised that there may be a temptation for students to change their diets before the trial based on their new learning. To avoid this, students were also specifically asked not alter their diet before the intervention.

Sample collection and measurement of urinary BPA

Urine samples were collected into BPA-free bottles (Vacutest Kima, Italy) immediately before and after the intervention, and were frozen at -20˚C within 4 hours. Each participant was sampled twice, once at visit 1 before the intervention and once at visit 2 after the

For peer review only

intervention. Sample collections were staggered to allow for the large number of participants passing through the facility, but students were sampled during the same time slot at both visits to account for circadian variation in BPA metabolism. The initial samples were collected during the early part of the day just prior to the students commencing the trial. The second samples were taken over the same time period 7 days later, just prior to the students recommencing their usual diet. Samples were transported on dry ice to a commercial laboratory (Rovaltain Research Company, Aixain, France) where analysis of total BPA was assessed by gas chromatography-tandem mass spectrometry. Experimental methods were validated for linearity, detection limit and accuracy and specificity of quantification based on the Standard NF T 90-201 for determination of xenobiotics. A quality control check of known standards injected every 6 samples at two levels of concentration (0.5 ng/ml and 5 ng/ml) was quantified with each batch of unknown samples. Water-only samples were included as negative controls. Urinary creatinine was measured at the Royal Devon and Exeter Hospital using the Jaffe method on the Roche P800 platform (Roche, Mannheim, Germany), to allow correction for urine dilution. Results were expressed as a BPA:creatinine ratio. Samples where BPA was detected but quantifying at or around the limits of quantification (LoQ) of 0.1ng/ml were scored as LoQ/√2 according to the method of Hornung and Reed [19].

Statistical Analysis

The difference between urinary BPA adjusted for creatinine between samples taken at visits 1 and 2 was assessed to generate a ΔBPA continuous variable. BPA risk scores were calculated as a continuous variable. The relationship between urinary BPA levels before and after the 7 day intervention was assessed using a repeated-measure ANOVA, adjusted for sex, time of

For peer review only

sampling and BMI, with and without correction for creatinine. The relationship between urinary BPA at visit 1 and whether or not the participants had lower BPA at visit 2 was also examined by binary logistic regression, adjusted for sex, time of sampling and BMI. Here, samples showing small changes < 0.5ng/ml in either direction were omitted to avoid natural stoichiometric variation around zero. The relationship between change in BPA (ΔBPA) and BPA risk score was assessed by linear regression, adjusted for sex, time of sampling and BMI both with and without adjustment for creatinine. Statistical analysis was carried out using SPSS, v.22 (IBM, USA).

Impact of following reduced BPA diet on lifestyle

We carried out quantitative and qualitative analysis to address long-term sustainability of the diet. Data on the impact of following the diet on feelings of dietary restriction, time spent sourcing or preparing meals, calorific intake and long term sustainability were collected via a questionnaire (See Supplementary information file 4). The questionnaire also included a freeform section where participants could write about their experiences following the diet in a non-prescribed fashion for qualitative analysis. Qualitative data was assessed for thematic content by two experienced qualitative researchers. Key themes were independently identified and coded until agreement was reached.

RESULTS

Participant Characteristics

For peer review only

There were 108 volunteer participants invited to participate in this engaged research study. A small number were absent or unable to produce a urine sample at both visits. A complete dataset was thus received from 94 students. Information on the characteristics of the study cohort are given in table 1.

Table 1: Characteristics of the study population. A complete dataset was available on 94 out of 108 participants. IQR = interquartile range, SD = Standard deviation. The units of BPA are ng/ml and BMI is defined as Kg/m^2. LoQ = limit of quantification. Urinary BPA levels are given both as unadjusted data and as a BPA (ng/ml) to creatinine (mg/ml) ratio.

Unadjusted urinary BPA at visit 1 (n = 98)	
median (IQR)	1.37 (2.52)
95% confidence intervals	1.58 to 2.57
mean (SD)	2.07 (2.51)
Number of samples below LoQ (0.1ng/ml)	16
Minimum value (ng/ml)	0.07
Maximum value (ng/ml)	13.55
Creatinine-adjusted urinary BPA at visit 1 (n = 98)	
median (IQR)	1.34 (1.82)
95% confidence intervals	1.38 to 2.13
mean (SD)	1.75 (1.82)
Number of samples below LoQ (0.1ng/ml)	16
Minimum value (ng/ml)	0.05
Maximum value (ng/ml)	9.52
Unadjusted urinary BPA at visit 2 (n = 99)	
median (IQR)	1.91 (2.68)
95% confidence intervals	2.15 to 4.56
mean (SD)	3.35 (6.18)
Number of samples below LoQ (0.1ng/ml)	12
Minimum value (ng/ml)	0.07
Maximum value (ng/ml)	49.6
Creatinine-adjusted urinary BPA at visit 2 (n = 99)	
median (IQR)	1.31 (2.24)
95% confidence intervals	1.46 to 8.34
mean (SD)	4.90 (16.8)
Number of samples below LoQ (0.1ng/ml)	12
Minimum value (ng/ml)	0.04
Maximum value (ng/ml)	139.33
Unadjusted ΔBPA (n = 94)	

For peer review only

median (IQR)	0.14
95% confidence intervals	0.15 to 2.41
mean (SD)	1.28 (5.79)
Minimum value	-8.02
Maximum value	49.5
Adjusted ΔBPA (n = 94)	
median (IQR)	0.02 (2.61)
95% confidence intervals	-0.23 to 6.53
mean (SD)	3.15 (16.5)
Minimum value	-8.6
Maximum value	133.45
BPA risk score (n = 99)	
median (IQR)	15.0 (10.3)
95% confidence intervals	15.5 to 18.4
mean (SD)	17.0 (7.12)
Demographics (n= 99)	
Sex - % male	44
Exposure to estrogens - % of cohort	14
BMI– median (IQR)	20.7 (3.45)
BMI– mean (SD)	21.2 (3.07)

BPA was detected in the urine of 86% of subjects at visit 1 prior to the intervention. Missing samples were due to non-attendance of participants or non-provision of a suitable sample. Samples below the limit of quantification were scored as 0.07 ng/ml (LoQ/√2).

Creatinine-adjusted urinary BPA concentrations do not change significantly after following an intervention diet designed to reduce BPA exposure for 7 days.

The median change in creatinine-adjusted urinary BPA between visits (ΔBPA) was 0.02 ng/ml with an interquartile range of 2.61 ng/ml. We identified no changes in urinary BPA between visits ($p = 0.25$; figure 1a). Three outliers with very high urinary BPA readings at visit 2 were excluded from the analysis, since these samples lay outside the linear range of analysis, so confidence in quantification was poor. No confounding factors included in the analysis were associated with change in BPA ($p = 0.78$, 0.43 and 0.36 for sex, time of sample collection and BMI respectively). We also identified no change in BPA levels between visits

For peer review only

using data uncorrected for creatinine (p = 0.20). We also assessed whether participants from different schools showed variable BPA levels at either visit 1, or change in BPA, but no such effects were noted.

Similarly, no relationship between change in urinary BPA (ΔBPA) and BPA risk score was identified (beta coefficient 0.08, standard error 0.07, p = 0.55; figure 1b). No associations were noted between change in urinary BPA and BPA risk score in data not adjusted for creatinine (p = 0.27). We found no association between ΔBPA and BPA risk score when considering only the exposure on the day prior to testing, taking into account the short half-life of BPA (p = 0.16 and p = 0.33 for adjusted and unadjusted data respectively).

Participants with highest starting urinary BPA levels were more likely to demonstrate lower BPA levels at visit 2.

We found an inverse relationship between initial BPA levels and whether a participant had reduced BPA levels at visit 2 (p = 0.003). These data indicate that the participants in the cohort with the highest creatinine-adjusted urinary BPA levels at visit 1 were more likely to demonstrate a drop in their urinary BPA at visit 2 (figure 2).

Following the intervention diet has significant effects on participant lifestyle

Participants indicated that following the diet had no significant cost implications on family finances, with 50% of participants reporting that it had cost more, and 50% reporting that

For peer review only

costs had decreased or remained the same. Although participants did not spend longer preparing their food, 78% of participants reported that their shopping took longer. Calorific intake was not affected for the majority of participants (58%) of participants. A large percentage of the cohort (91%) reported that they felt at least slightly restricted in their food choices and 27% of participants reported that they felt very restricted. Finally, 66% of participants stated that they would find it hard or very hard to follow the diet long term.

Qualitative analysis of the effect of following the diet on lifestyle

We identified 5 overriding themes in our qualitative analysis of the effect of following the diet on lifestyle. These were 1) the widespread use of plastics possibly containing BPA in food packaging (“almost everything is packaged in plastic” – participant 70, “Literally everything involved plastic” – participant 28). 2) Lack of clarity in labelling of products and packaging potentially containing BPA (“I found it really hard to know what foods I could eat … there is never a guarantee it is BPA free” – participant 43, " The biggest problem was that a lot of packaging doesn’t state what type of plastic it is or whether it contains BPA” – participant 74). 3) The perceived restrictions of being on the ‘real world’ BPA avoidance diet (“Difficulty eating out, hard to find foods in college or ‘out’ that hadn’t touched BPA. My family had a takeaway on Saturday night and I couldn’t eat it” – participant 56, “Sometimes I can’t eat / drink what I want because of the recycling number” – participant 112). 4) The impact of eating ‘BPA free’ was the only positive theme emerging (“I feel I have eaten much more healthily this week … I didn’t eat so much junk food” – participant 74, “I ate more vegetables and less chocolate” – participant 83). 5) The impact on shopping habits (“You can’t get it all from supermarkets” – Participant 37; “Had to go to more individual food shops” – participant 103).

For peer review only

DISCUSSION

Exposure to the endocrine disrupting chemical Bisphenol A (BPA) is ubiquitous [2], with growing evidence that it may be associated with adverse health outcomes [4]. Here, 94 researcher participants aged 17-19 years designed and undertook a quantitative and qualitative engaged research project designed to assess the potential for reduction of personal exposure to BPA through moderation of diet, which would have utility in a 'real world' setting. We conclude that the 'real world' diet designed to reduce BPA exposure had no effect on creatinine-adjusted urinary BPA concentrations in our cohort over a period of 7 days in our dataset.

Although levels of urinary BPA in our study cohort were slightly lower at the outset of the study in our cohort than in others [18], measureable levels were present in the vast majority of our participants. Participants were unable to achieve a reduction in their urinary BPA levels over the 7 day trial period, despite good compliance to supplied guidelines. Avoidance of BPA was not easily achieved on an individual level in our study population, with qualitative analysis indicating that participants experienced feelings of restriction and difficulties in sourcing BPA-free food due to inadequate labelling of foods and food packaging. This suggests that the intervention would be difficult to sustain in the longer term.

This work represents the largest group of unrelated participants in a high exposure demographic_to date, since previous work has focused on families and related individuals [16] [17], who may share common sources of BPA. Although other population demographics

For peer review only

such as young children may have higher levels of BPA than our chosen study population [18], it would not have been possible to do the sort of engaged research project that we envisaged in this group. Our intervention is a ‘real world’ diet, designed to a set of guidelines (such as reduction in the usage of tinned foods or foods with high levels of processing), rather than the strict, prescribed diets that have been used in other studies [16], which suggested that it was possible for participants to reduce their urinary BPA excretion by approximately 60% in a period of just 3 days [16]. In our self-designed, self-administered study this was unachievable. This may reflect the difficulty in identifying and sourcing foods free of BPA in our commercial environment. Finally, the qualitative thematic analysis we carried out in our study has given an indication that adherence to even a ’real world’ BPA reduction diet with fewer restrictions and more choice over the longer term was unlikely in our study population due to difficulties in identifying foodstuffs likely to contain less BPA.

BPA has a terminal half-life of 6 hours [15]. Spot samples may therefore not be as accurate as continuous sampling strategies (24hr urine collection). However, recent studies suggest that despite its short half-life, measureable BPA remains present for up to 43 hours post-fasting, indicating non-food exposures or accumulation in body tissues such as fat [20]. We identified no impact of time of sample collection on BPA levels in our sample set, in either creatinine-adjusted or unadjusted data, indicating that our measurements were not influenced by time since the last meal. Spot sampling as used here may therefore represent an acceptable compromise and remains a practical option in the community setting of our study. The large variability in urinary BPA levels within an individual sampled at different times may also have reduced our ability to observe an effect. This could be facilitated by the use of multiple sampling, or pools of multiple urines, but was not feasible within the confines of our study.

For peer review only

Calculating an accurate BPA risk score is challenging. Data were self-reported, and foodstuffs are not labelled for BPA content. It is difficult to generalise across food types and large variations in BPA concentrations occur between different products of the same food type or even different lots of the same product [1]. Foods that were free of BPA-containing packaging (as far as it was possible to tell) may have been highly processed or contain food items from a variety of sources. Highly processed and 'fast' food has previously been demonstrated to be a source of BPA [21]. A study of the temporal trends seen in composite food samples found no change in the overall BPA content of the food, despite large reduction in the BPA content of some individual food items, illustrating the difficulties in effectively excluding BPA from a varied diet [22]. Participants may therefore have changed BPA containing foods for other, perceived healthier choices, which may still contain BPA by virtue of processing.

BPA enters foodstuffs by leaching from polycarbonate or epoxy resin after manufacture, or by hydrolysis of the polymer itself [23]. The migration rate of BPA increases with higher temperatures [24], and with time and use, e.g. repeated use of polycarbonate water bottles [25]. Exposure to BPA can also occur through routes other than food, including dust ingestion and dermal absorption [26] and this was not taken into account in our study. A study of volunteers who purposefully handled thermal receipts showed an increase in urinary BPA excretion of up to 84%, and their BPA levels took longer to return to pre exposure levels, suggesting a difference in the bio-availability of BPA through skin and oral routes [27]. It is also possible that some manufacturers may have voluntarily reduced the amount of BPA-containing food packaging compared to their previous usage, given the attention that

For peer review only

endocrine disrupting chemicals have received in the media. However, measurable levels of BPA were still detected in the majority of participants in our study, which suggests that there may be other, non-dietary, sources of BPA, and that exposure to BPA remains an issue. We may also have been underpowered to detect subtle changes in urinary BPA, given the heterogeneity in food choice; detection of such effects may need thousands of participants. Finally, our study, like other studies of its type, does not take account of inter-individual differences in the metabolism and excretion of BPA arising from differences in genetic background between people. BPA is metabolised primarily by UDP-glucuronosyltransferases, and altered activity polymorphisms of these enzymes have been reported [28].

Emerging evidence suggests that that BPA may be linked to several chronic human health conditions [6-9, 29], suggesting that continued study of the human health effects of BPA exposure is justified. The opinion of the European Food Safety Authority (EFSA), is that whilst uncertainty over the human health effects of BPA exists, caution should be exercised in ingestion of BPA [3]. Our data suggests that in our study population, it is unlikely that participants could moderate their own BPA exposure in the long term by self-directed modification of diet in a 'real world' setting, and furthermore, participants would have been reluctant to adopt such a lifestyle change in the longer term due to the restrictions in dietary choice and the effects on day to day life. Most of these barriers appear to arise from the pervasiveness of BPA in our food chain, and inadequate labelling of foods packaged in BPA-containing substances. We propose that until a definitive assessment of the health risks of BPA is available, informed choice over whether or not to consume BPA and similar chemicals in foodstuffs should be facilitated by better labelling.

For peer review only

CONTRIBUTORSHIP STATEMENT

TSG - Contributed to study design and co-wrote the paper

NB - Contributed to study design and participant involvement

BP - Managed the technical aspects of the project and reviewed the manuscript

ALK - Contributed to data entry and interpretation and reviewed the manuscript

BPA Schools Study Consortium members - designed and interpreted the study and contributed to the manuscript.

MHS - Carried out the qualitative analysis and reviewed the manuscript

AMS - Managed sample collection, contributed to study design and reviewed the manuscript.

LWH - PI , managed the study, wrote the manuscript

ACKNOWLEDGEMENTS

We acknowledge the work of the NIHR Exeter Clinical Research Facility in aiding the collection of the urine samples.

COMPETING INTERESTS

The authors have no competing interests to declare.

For peer review only

DATA SHARING STATEMENT

Data are available upon reasonable request by emailing Lorna Harries (L.W.Harries@exeter.ac.uk).

FUNDING

This study was funded by a Wellcome Trust People Award to LWH and TG (grant number 105162/Z/14/Z).

REFERENCES

1 Geens T, Aerts D, Berthot C*, et al.* A review of dietary and non-dietary exposure to bisphenol-A. *Food Chem Toxicol* 2012;**50**:3725-40.

2 WHO. World Health Organisation Background paper on mechanisms of action of bisphenol A and other biochemical/molecular interactions. *WHO/HSE/FOS* 2010;**11.1**.

3 EFSA. Scientific opinion on the risks to public health related to the presence of bisphenol A (BPA) in foodstuffs. *EFSA journal* 2015;**13**.

4 Rochester JR. Bisphenol A and human health: a review of the literature. *Reprod Toxicol* 2013;**42**:132-55.

For peer review only

5 WHO. Joint FAO/WHO Expert meeting to review toxicological and health aspects of bisphenol A Summary report. 2010.

6 Galloway T, Cipelli R, Guralnik J, *et al.* Daily bisphenol A excretion and associations with sex hormone concentrations: results from the InCHIANTI adult population study. *Environ Health Perspect* 2010;**118**:1603-8.

7 Melzer D, Rice NE, Lewis C, *et al.* Association of urinary bisphenol a concentration with heart disease: evidence from NHANES 2003/06. *PLoS One* 2010;**5**:e8673.

8 Song Y, Chou EL, Baecker A, *et al.* Endocrine-disrupting chemicals, risk of type 2 diabetes, and diabetes-related metabolic traits: A systematic review and meta-analysis. *J Diabetes* 2015.

9 Savastano S, Tarantino G, D'Esposito V, *et al.* Bisphenol-A plasma levels are related to inflammatory markers, visceral obesity and insulin-resistance: a cross-sectional study on adult male population. *J Transl Med* 2015;**13**:169.

10 Braun JM. Early-life exposure to EDCs: role in childhood obesity and neurodevelopment. *Nat Rev Endocrinol* 2017;**13**:161-73.

11 Vandenberg LN, Chahoud I, Heindel JJ, *et al.* Urinary, circulating, and tissue biomonitoring studies indicate widespread exposure to bisphenol A. *Environ Health Perspect* 2010;**118**:1055-70.

12 Gore AC, Chappell VA, Fenton SE, *et al.* EDC-2: The Endocrine Society's Second Scientific Statement on Endocrine-Disrupting Chemicals. *Endocr Rev* 2015;**36**:E1-E150.

For peer review only

13 Agency EC. AGREEMENT OF THE MEMBER STATE COMMITTEE ON THE IDENTIFICATION OF 4,4'-ISOPROPYLIDENEDIPHENOL (BISPHENOL A) AS A SUBSTANCE OF VERY HIGH CONCERN 2017.

14 Lakind JS, Naiman DQ. Daily intake of bisphenol A and potential sources of exposure: 2005-2006 National Health and Nutrition Examination Survey. *Journal of exposure science & environmental epidemiology* 2011;**21**:272-9.

15 Dekant W, Volkel W. Human exposure to bisphenol A by biomonitoring: methods, results and assessment of environmental exposures. *Toxicol Appl Pharmacol* 2008;**228**:114-34.

16 Rudel RA, Gray JM, Engel CL*, et al.* Food packaging and bisphenol A and bis(2-ethyhexyl) phthalate exposure: findings from a dietary intervention. *Environ Health Perspect* 2011;**119**:914-20.

17 Sathyanarayana S, Alcedo G, Saelens BE*, et al.* Unexpected results in a randomized dietary trial to reduce phthalate and bisphenol A exposures. *Journal of exposure science & environmental epidemiology* 2013;**23**:378-84.

18 Calafat AM, Ye X, Wong LY*, et al.* Exposure of the U.S. population to bisphenol A and 4-tertiary-octylphenol: 2003-2004. *Environ Health Perspect* 2008;**116**:39-44.

19 Hornung R, Reed L. Estimation of average concentration in the presence of nondetectable values. *Appl Occupat Environ Hyg* 1990;**5**:46–51.

For peer review only

20 Stahlhut RW, Welshons WV, Swan SH. Bisphenol A data in NHANES suggest longer than expected half-life, substantial nonfood exposure, or both. *Environ Health Perspect* 2009;**117**:784-9.

21 Zota AR, Phillips CA, Mitro SD. Recent Fast Food Consumption and Bisphenol A and Phthalates Exposures among the U.S. Population in NHANES, 2003-2010. *Environ Health Perspect* 2016;**124**:1521-8.

22 Cao XL, Perez-Locas C, Robichaud A*, et al.* Levels and temporal trend of bisphenol A in composite food samples from Canadian Total Diet Study 2008-2012. *Food Addit Contam Part A Chem Anal Control Expo Risk Assess* 2015;**32**:2154-60.

23 Aschberger K, Castello P, Hoekstra E*, et al.* Bisphenol A and baby bottles: challenges and perspectives. 2010.

24 Le HH, Carlson EM, Chua JP*, et al.* Bisphenol A is released from polycarbonate drinking bottles and mimics the neurotoxic actions of estrogen in developing cerebellar neurons. *Toxicol Lett* 2008;**176**:149-56.

25 Brede C, Fjeldal P, Skjevrak I*, et al.* Increased migration levels of bisphenol A from polycarbonate baby bottles after dishwashing, boiling and brushing. *Food Addit Contam* 2003;**20**:684-9.

26 Myridakis A, Chalkiadaki G, Fotou M*, et al.* Exposure of Preschool-Age Greek Children (RHEA Cohort) to Bisphenol A, Parabens, Phthalates, and Organophosphates. *Environ Sci Technol* 2016;**50**:932-41.

For peer review only

27 Lv Y, Lu S, Dai Y, *et al.* Higher dermal exposure of cashiers to BPA and its association with DNA oxidative damage. *Environ Int* 2017;**98**:69-74.

28 Stingl JC, Bartels H, Viviani R, *et al.* Relevance of UDP-glucuronosyltransferase polymorphisms for drug dosing: A quantitative systematic review. *Pharmacol Ther* 2014;**141**:92-116.

29 Melzer D, Osborne NJ, Henley WE, *et al.* Urinary bisphenol A concentration and risk of future coronary artery disease in apparently healthy men and women. *Circulation* 2012;**125**:1482-90.

For peer review only

FIGURE LEGENDS

Figure 1. The effect of a 'real world' BPA avoidance diet on urinary BPA exposure over a 7 day period. **A**. Urinary BPA levels (ng/ml) adjusted for urinary creatinine were plotted at visit 1 before the intervention and at visit 2 after the intervention. The 3 extreme outliers have been removed. The trajectories of individual participant measurements are shown. **B**. Change in urinary BPA levels in ng/ml following the intervention diet are plotted against the self-reported BPA risk score.

Figure 2. The effect of baseline urinary BPA on the probability of achieving a drop in levels following the intervention. This graph illustrates the median urinary BPA level adjusted for creatinine at visit 1 prior to the intervention expressed relative to whether or not a reduction in urinary BPA levels was achieved following the 7 day intervention diet at visit 2. Error bars refer to the interquartile range of measurement.

For peer review only

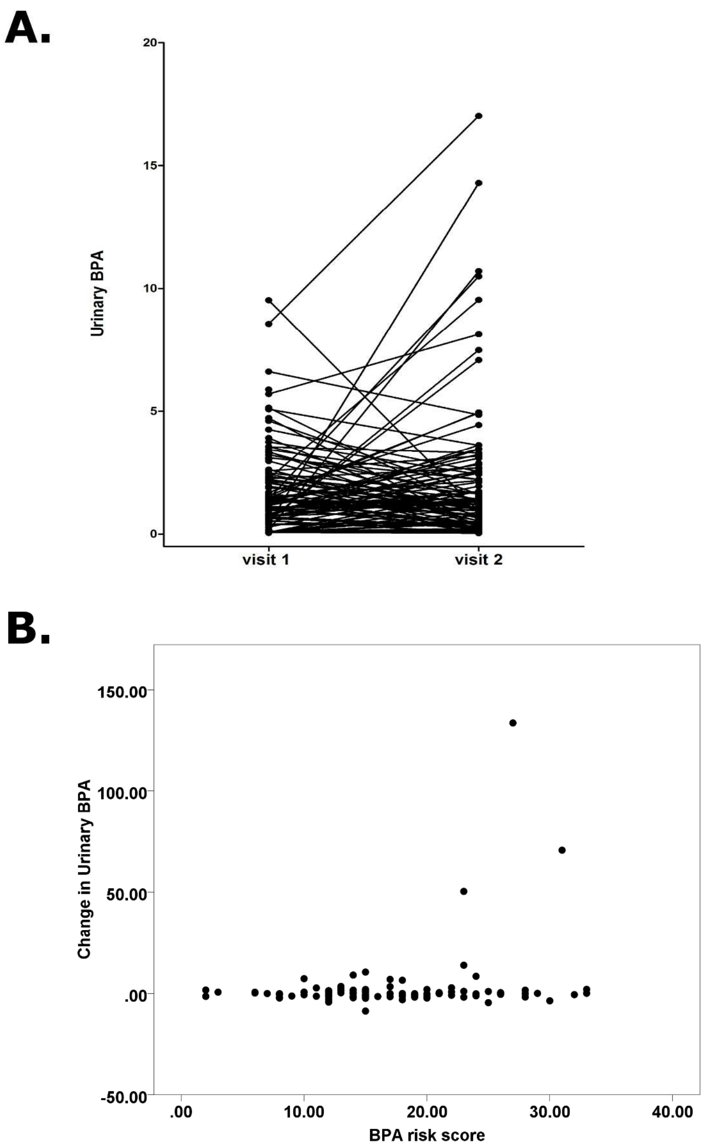

Figure 1

209x297mm (300 x 300 DPI)

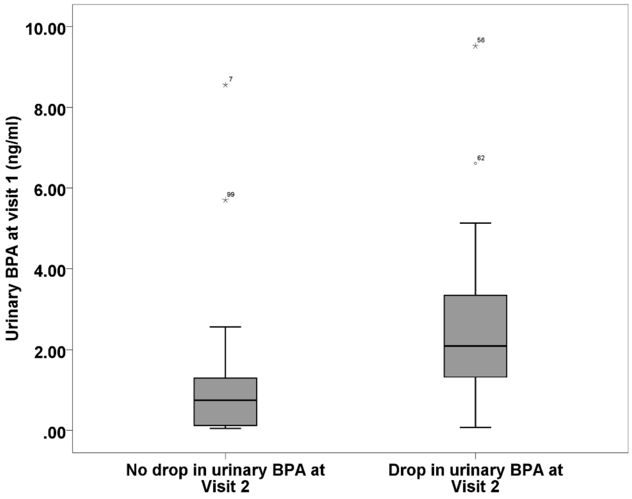

Figure 2

209x297mm (300 x 300 DPI)

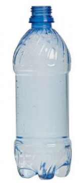

BPA: Myth and Reality
Dietary Intervention Guidelines

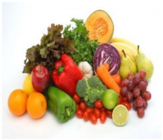

General instructions

The purpose of this dietary intervention trial is to follow a diet designed to minimise routes of exposure to the food packaging chemical bisphenol A (BPA). For the dietary intervention period, please follow as closely as possible the instruction given below. Try to maintain your diet during the intervention period to be as closely similar to your normal diet as possible, in terms of the content, amount and calorific value of the food you eat. Please record details of each meal and the drinks and snacks you consume on the forms provided. Below are some general cooking and eating tips and an indication of which foods are best to avoid and those that are considered a low source of BPA.

Cooking and eating tips for the intervention period.

The general approach is to replace any food items that fall into the 'avoidance' category with an alternative, chosen to minimise exposure to BPA

- **Switch to stainless steel and glass food storage and drink containers.**
- **Move foods to ceramic or glass food containers before microwaving.**
- **Consider a coffee filter or percolator for coffee – home coffee makers (Such as Nespresso^{TM}) may have polycarbonate-based water tanks and phthalate-based tubing.**
- **Eat out less, especially at restaurants that do not use fresh ingredients.**
- **Avoid canned food consumption. Where possible, replace with fresh produce or cardboard or tetrapack packaged alternatives.**
- **Choose fresh fruits and vegetables when possible, and frozen if not.**
- **Soak dried beans for cooking rather than tinned.**

For peer review only - http://bmjopen.bmj.com/site/about/guidelines.xhtml

For peer review only

Foods to avoid

Tinned foods. Top ten tinned foods that are reported to be sources of BPA include coconut milk, soup, meat, vegetables, meals (e.g. pasta with sauce), juice, fish, beans, meal replacement drinks, fruit.

Carbonated/fizzy drinks and juices in cans. Avoid carbonated drinks in cans and drinks stored for prolonged periods in reusable sports bottles, unless they are labelled 'BPA free' (many commercial sports bottles are).

Fast food from commercial outlets. Most processed food has passed through numerous processes, and each additional processing step provides an opportunity for BPA to enter through packaging or tubing. Try to replace fast and processed foods with a freshly prepared and cooked alternative.

Packaged fruit and vegetables. Replace these where possible with unpackaged, loose fruit and vegetable items as far as possible.

Convenience/ready meals. Plastics types considered safest in terms of chemical migration are recycling numbers 2 and 5. Avoid food prepared in packaging with recycling number 7, which includes many different types of polymer and mixed polymers, including polycarbonate, a source of BPA. Try to avoid foods that are designed to be heated in the microwave in their packaging.

Chocolate and ice cream. Individuals who report eating chocolate bars and ice cream on a regular basis have been reported to have higher than average BPA exposure. Try to avoid excessive consumption.

Non-food or food packaging routes of exposure

Although plastics found in consumer goods such as DVDs, CDs, computer goods and sunglasses do contain BPA, this is not an important route of exposure.

Till receipts often contain high levels of BPA, so wash your hands before eating or drinking if you have been handling them.

Dental sealants may contain BPA, so avoid any pre-planned dental work

Example daily diet

Food Item	Comments
**Breakfast**	
Cereal, Fruit	
Milk	Polypropylene or glass packaging
Bread	
Yoghurt	Choose polypropylene container
**Lunch**	
Meat or fish products	Check packaging and avoid those labelled no. 7. Avoid tinned ingredients
Cheese	
Salad items, Fruit	Choose unpackaged where possible, wash before use
Pasta	
**Dinner**	
Shepherds pie	Cooked in saucepan and oven rather than microwaved in plastic
Green beans	Fresh or frozen
Bread	
**Drinks**	
Water	Water direct from tap or use stainless steel or BPA free water bottle
Tea/coffee	Prepare in teapot or cafetiere, avoid commercial coffee makers
Carbonated drinks	Avoid canned drinks and those stored in reusable containers for prolonged periods
Milk	Polypropylene or glass packaging
**Snacks**	
Fruit	
Potato crisps	

For peer review only

Place participant barcode here	FOOD - DAY 1		DRINK – DAY 1	
**Contents of meal**	Was food packaged in plastic known or suspected to contain BPA?	Was food cooked in plastic known or suspected to contain BPA?	**Type of Drink**	Packaging (please describe) e.g. canned, plastic (Δ number), tetrapak
**Breakfast:**				
1.				
2.				
3.				
4.				
5.				
**Lunch**:				
1.				
2.				
3.				
4.				
5.				
**Dinner**:				
1.				
2.				
3.				
4.				
5.				
**Snacks**:				
1.				
2.				
3.				
4.				

For peer review only

Place participant barcode here	FOOD - DAY 2		DRINK – DAY 2	
**Contents of meal**	Was food packaged in plastic known or suspected to contain BPA?	Was food cooked in plastic known or suspected to contain BPA?	**Type of Drink**	Packaging (please describe) e.g. canned, plastic (Δ number), tetrapak
**Breakfast:**				
1.				
2.				
3.				
4.				
5.				
**Lunch**:				
1.				
2.				
3.				
4.				
5.				
**Dinner**:				
1.				
2.				
3.				
4.				
5.				
**Snacks**:				
1.				
2.				
3.				
4.				

For peer review only

Place participant barcode here	FOOD - DAY 3		DRINK – DAY 3	
**Contents of meal**	Was food packaged in plastic known or suspected to contain BPA?	Was food cooked in plastic known or suspected to contain BPA?	**Type of Drink**	Packaging (please describe) e.g. canned, plastic (Δ number), tetrapak
**Breakfast:**				
1.				
2.				
3.				
4.				
5.				
**Lunch**:				
1.				
2.				
3.				
4.				
5.				
**Dinner**:				
1.				
2.				
3.				
4.				
5.				
**Snacks**:				
1.				
2.				
3.				
4.				

For peer review only

Place participant barcode here	FOOD - DAY 4		DRINK – DAY 4	
**Contents of meal**	Was food packaged in plastic known or suspected to contain BPA?	Was food cooked in plastic known or suspected to contain BPA?	**Type of Drink**	Packaging (please describe) e.g. canned, plastic (Δ number), tetrapak
**Breakfast:**				
1.				
2.				
3.				
4.				
5.				
**Lunch**:				
1.				
2.				
3.				
4.				
5.				
**Dinner**:				
1.				
2.				
3.				
4.				
5.				
**Snacks**:				
1.				
2.				
3.				
4.				

For peer review only

Place participant barcode here	FOOD - DAY 5		DRINK – DAY 5	
**Contents of meal**	Was food packaged in plastic known or suspected to contain BPA?	Was food cooked in plastic known or suspected to contain BPA?	**Type of Drink**	Packaging (please describe) e.g. canned, plastic (Δ number), tetrapak
**Breakfast:**				
1.				
2.				
3.				
4.				
5.				
**Lunch**:				
1.				
2.				
3.				
4.				
5.				
**Dinner**:				
1.				
2.				
3.				
4.				
5.				
**Snacks**:				
1.				
2.				
3.				
4.				

For peer review only

Place participant barcode here	FOOD - DAY 6		DRINK – DAY 6	
**Contents of meal**	Was food packaged in plastic known or suspected to contain BPA?	Was food cooked in plastic known or suspected to contain BPA?	**Type of Drink**	Packaging (please describe) e.g. canned, plastic (Δ number), tetrapak
**Breakfast:**				
1.				
2.				
3.				
4.				
5.				
**Lunch**:				
1.				
2.				
3.				
4.				
5.				
**Dinner**:				
1.				
2.				
3.				
4.				
5.				
**Snacks**:				
1.				
2.				
3.				
4.				

For peer review only

Place participant barcode here	FOOD - DAY 7		DRINK – DAY 7	
**Contents of meal**	Was food packaged in plastic known or suspected to contain BPA?	Was food cooked in plastic known or suspected to contain BPA?	**Type of Drink**	Packaging (please describe) e.g. canned, plastic (Δ number), tetrapak
**Breakfast:**				
1.				
2.				
3.				
4.				
5.				
**Lunch**:				
1.				
2.				
3.				
4.				
5.				
**Dinner**:				
1.				
2.				
3.				
4.				
5.				
**Snacks**:				
1.				
2.				
3.				
4.				

For peer review only

Participant barcode

Additional study information.

Please do not feel obliged to answer these questions if you are uncomfortable doing so.

Gender

☐ Female
☐ Male
☐ Prefer not to say

Tobacco Usage – Have you used tobacco over the past week

☐ Yes
If so, what type and how much?_______________________________________
☐ No
☐ Prefer not to say

Alcohol Usage – Have you used alcohol over the past week

☐ Yes
If so, what type and how much?_______________________________________
☐ No
☐ Prefer not to say

Medication- Have you taken any medication over the last week?

☐ Yes
☐ No
☐ Prefer not to say

If so, Please name the medication_____________________ ☐ Prefer not to say

Vegetarian/vegan diet - Have you eaten or drank any soya products over the past week?

☐ Yes
☐ No
☐ Prefer not to say

Your measurements - leave blank if you prefer not to say

Your height ___________________________

Your weight___________________________

For peer review only

<u>Supplementary information file 3. Example daily diet diary</u>. A score of 1 is given to each item containing suspected to contain BPA or be packaged in BPA-containing materials. Highly processed foods are also scored as 1, due to uncertainties in the processing procedures. The daily totals are summed to produce a BPA risk score for the 7 day intervention.

	**Item**	**Packaging**	**Score**
**Breakfast**			
	Homemade pancakes	None	0
	Sugar	None	0
	lemon	None	0
	milk	HDPE	0
**Lunch**			
	Homemade Cheese sandwich	none	0
	Homemade sultana cake	none	0
	water	glass	0
**Dinner**			
	Homemade omelette	none	0
	Sweetcorn	Can	1
	Rice	Cellophane	0
	Tomatoes	none	0
	water	glass	0
**Snacks**			
	Crisps (processed)	Cellophane	1
	Apple	none	0
	milk	HDPE	0
		**Total for day**	**2**

For peer review only

Participant Barcode

BPA: Myth and Reality diet questionnaire

1. Were there any times during the week that you knowingly/unknowingly did not stick to the diet? Please tick any that apply and give indication of frequency.

School meals ☐ ____ times

Restaurants/cafés ☐ ____ times

Friends' houses ☐ ____ times

Takeaway ☐ ____ times

Other ______________________________ ☐ ____ times

2. If you heated your food in a microwave, what was the food in? Tick any which apply and give indication of frequency.

A food storage container or bowl known or suspected to contain BPA ☐____times

3. When you or your family drank water, where did your water come from? Tick any which apply and give indication of frequency.

Plastic filter jug known or suspected to contain BPA ☐____times

Individual water bottle known or suspected to contain BPA ☐____times

Larger water container known or suspected to contain BPA ☐____times

4. How many times during the week did you eat food that had been stored or transported in plastic containers known or suspected to contain BPA?

____

5. How many times during the week did you eat tinned food or drink from cans?

____

6. Did the BPA reduced diet affect How much you spent on shopping?

Spent more ☐

Spent less ☐

No difference ☐

Participant questionnaire V3 25Jun15

For peer review only

Participant Barcode

7. Did it take longer to source your food than usual?

Yes ☐ No ☐

If so, why? ______________________________

____

8. Did it take longer to prepare food than usual?

Yes ☐ No ☐

If so, why? ______________________________

____

9. How restricted did you feel by your food choice?

☐ Very ☐ Slightly ☐ No difference

If you felt you were restricted by the diet, why was this? ______________________________

____

10. Did the diet affect your calorific intake?

Yes ☐ No ☐

If so, why? ______________________________

____

For peer review only - http://bmjopen.bmj.com/site/about/guidelines.xhtml

For peer review only

Participant Barcode

11. How easy would you find it to sustain this diet over a longer period of time?

Very easy ☐

Easy ☐

Hard ☐

Very hard ☐

Not sure ☐

12. Is there anything else about following the diet that you would like to add?

For

For peer review only - http://bmjopen.bmj.com/site/about/guidelines.xhtml

Thank you for reading this leaflet. If you wish to participate in this study, you will be asked to agree to the consent statements below in the presence of a member of the research team.

CONSENT STATEMENTS

1. I confirm that I have read this information sheet and have discussed participation in this project with my family. I have had opportunity to consider the information, ask questions and have had these questions answered satisfactorily. *You should not give consent until you are happy that you understand what the study involves.*

2. I understand that my participation is voluntary and that I am free to withdraw at any time without giving any reason, without my right to participate in the rest of the study being affected. *This means that even if you helped design this study you do not have to be a participant and you should not feel under any pressure to participate.*

3. I agree to participate in this study as a research subject. *This means that you agree to participate in a one-week diet and to provide two blood and urine samples.*

4. I understand that my anonymised blood and urine samples and linked anonymous questionnaire data will be sent to University of Exeter Medical School, Royal Devon & Exeter Hospital and my urine sample only will be sent to the Rolvaltain laboratory, a specialist BPA analysis company. *This means that laboratory staff will not know that samples belong to you, but dedicated staff at the University of Exeter, with training and experience in data protection ,will be able to link your sample data to your questionnaire data.*

5. I understand that RNA (genetic material) will be extracted from my blood and will be stored anonymously. *This means that Professor Harries' team will use our RNA to provide data that you will help analyse but may also do further research on the samples to identify reasons for any changes seen.*

6. I understand that data relating to my participation in the study will be returned anonymously to my school to be used for educational purposes. *This means that although you will get to analyse data from your samples there is no way you will know which data relates to your samples and which to other participants*.

Complaints:

If you have any complaints about the way in which this study has been carried out please contact the Chair of the University of Exeter Medical School Research Ethics Committee Peta Foxall PhD, Chair, UEMS Research Ethics Committee: P.J.D.Foxall@exeter.ac.uk.

This project has been reviewed and approved by the University of Exeter Medical School Research Ethics committee UEMS REC REFERENCE NUMBER: 15/07/074)

Information Sheet - Version 4 (2/8/15)

BPA: Myth or Reality?

A research study investigating the effect of chemicals in plastic on gene activity and whether dietary interventions can reduce BPA levels in teenagers.

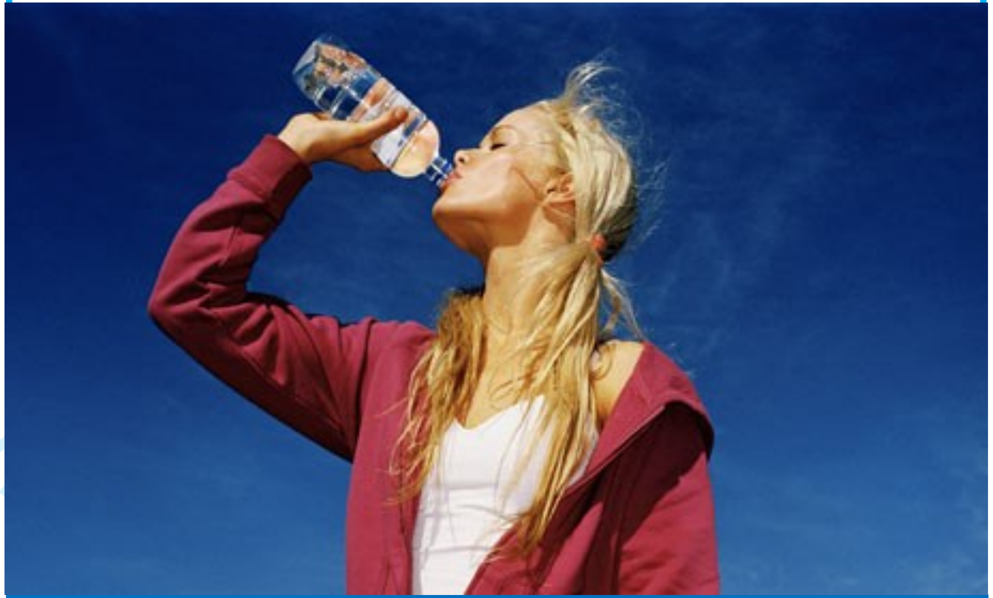

Involvement & Engagement

The aim of this year-long project is to involve teenagers in a research study that is relevant to them, by allowing them to help design a research project, analyse non-identifiable participant data and help to present and publish the outcomes.

Participation

Students will be asked to undertake a one week diet to reduce their intake of BPA, a chemical found in plastics. They will be asked to provide urine and blood samples before and after their diet.

For peer review only - http://bmjopen.bmj.com/site/about/guidelines.xhtml

What is BPA?

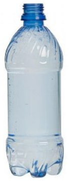

BPA (Bisphenol A) is a chemical used in the manufacture of plastics. Plastics containing BPA are found in a wide range of products including food and drink containers. BPA in these products can be ingested and there are concerns that high BPA levels in the blood could possibly affect human health. Research is therefore needed to understand its effects on the human body and how we can reduce its consumption by minor changes to our diet.

This project is being run as a student-involvement project to answer two specific questions:

1. **Can we see the effects of dietary BPA on our genes?**
2. **Can we effectively reduce BPA in our diet?**

In the past, small-scale experiments have shown that BPA levels in the human body can be reduced by rigid dietary interventions but these interventions would be difficult to implement in the “real world”. In this study a one-week dietary intervention designed by teenagers will be used by them to determine whether BPA levels, and the activity of BPA-responsive genes can be effectively reduced in young people by avoiding food packaging that contains this chemical.

What will I need to do?

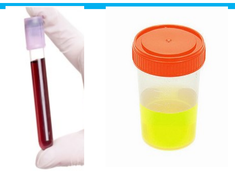

M

Day 1

- Provide a nurse with a 2.5ml blood sample and a urine sample.

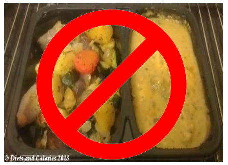

T

W

T

Day 2 - Day 8

- Follow a diet that you have helped to design .
- The diet will exclude sources of BPA as much as possible but will be nutritionally and calorifically similar to your usual diet.
- You will be asked to complete a food diary and answer a questionnaire about how easy it was to follow this diet.

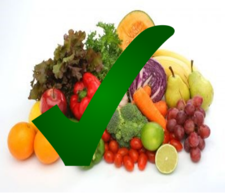

F

S

S

Day 8

- Provide a nurse with a 2.5ml blood sample and a urine sample.

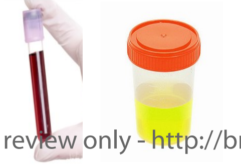

M

We recommend that you discuss the project with your family and involve them in planning what you eat and how you will prepare it.

What will happen to my samples and data?

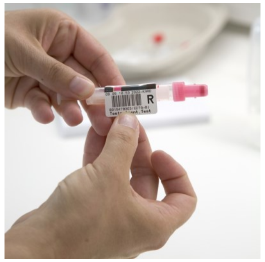

When you participate in the study you will be allocated with a numerical study ID. Your samples and data will be labelled with this number so that we can match your ‘before’ and ‘after’ diet samples with your food diary data. Once all data has been collated and coded it will be further anonymised by a person external to the project so that no data can be linked to any of the participants.

Urine samples from before and after the diet will be sent to the Royal Devon & Exeter NHS Foundation Trust for creatinine analysis and to the Rolvaltain laboratory for BPA analysis. RNA will be extracted from blood samples at the Royal Devon & Exeter Molecular Genetics Laboratory and the expression levels of two BPA-responsive genes will be measured in the samples taken before and after the diet. These anonymised RNA samples will be stored and used only by Professor Harries team for further research on the mechanisms behind our findings.

What are the benefits of taking part?

This project will help you to understand how you might be able to reduce BPA in your diet and your involvement in the design will give you an excellent insight into clinical research, community outreach and scientific practise. Your role as a participant is unlikely to have any direct health benefits.

Are there any risks in taking part?

Blood samples will be taken by fully qualified and insured NHS personnel. Any potential discomfort or side-effects will be equivalent to that experienced giving a blood sample to your GP. All data will be fully anonymised before analysis. This means that you will not find out anything about your blood or urine samples. Following the diet may minimally increase the cost of your groceries for the week, but since fresh foods are usually less expensive than pre-packaged foods, we do not expect this to be an issue.

What will happen to the results of the research study?

You will be given the opportunity to help analyse anonymised data from this project and to help disseminate the outcomes of this research. It is hoped that the findings will be published in peer-reviewed journals and the wider media.

Who is organising this research?

The research is organised by Professors Lorna Harries & Tamara Galloway of the University of Exeter as part of their research program into BPA and part of the University’s outreach program to involve schools in academic research.

Version 4 (2/8/2015)

BPA: Myth & Reality

STUDY ID

**CONSENT STATEMENTS**	**Please circle**
1. I confirm that I have read information sheet BPA PIS Version 4 and have discussed participation in this project with my family. I have had opportunity to consider the information, ask questions and have had these questions answered satisfactorily. *You should not give consent until you are happy that you understand what the study involves.*	**YES / NO**
2. I understand that my participation is voluntary and that I am free to withdraw at any time without giving any reason, without right to participate in the rest of the study being affected. *This means that even if you helped design this study you do not have to be a participant and you should not feel under any pressure to participate.*	**YES / NO**
3. I agree to participate in this study as a research subject. *This means that you agree to participate in a one-week diet and to provide two blood and urine samples.*	**YES / NO**
4. I understand that my anonymised blood and urine samples and linked anonymous questionnaire data will be sent to University of Exeter Medical School, Royal Devon & Hospital and my urine sample only will be sent to the Rolvaltain laboratory, a specialist BPA analysis company. *This means that laboratory staff will not know that samples belong to you, but dedicated staff at the University of Exeter, with training and experience in data protection, will be able to link your sample data to your questionnaire data.*	**YES / NO**
5. I understand that RNA (genetic material) will be extracted from my blood and will be stored anonymously. *This means that Professor Harries' team will use our RNA to provide data that you will help analyse but may also do further research on the samples to identify reasons for any changes seen.*	**YES / NO**
6. I understand that data relating to my participation in the study will be returned anonymously to my school to be used for educational purposes. *This means that although you will get to analyse data from your samples there is no way you will know which data relates to your samples and which to other participants*.	**YES / NO**

Name of Participant	Signature	Date
Name of Person Obtaining Consent	Signature	Date

I the above signed testify the participant is providing voluntary and fully informed consent to participate in this study. I am on the delegation log to obtain consent for this study and are trained in obtaining consent.

This project has been reviewed and approved by the University of Exeter Medical School Research Ethics Committee UEMS REC REFERENCE NUMBER: 15/07/074)

For peer review only

Version 4 (2/8/2015)

BPA: Myth & Reality

For peer review only

STROBE Statement—checklist of items that should be included in reports of observational studies

	Item No	Recommendation
**Title and abstract**	1	(*a*) Indicate the study's design with a commonly used term in the title or the abstract
		(*b*) Provide in the abstract an informative and balanced summary of what was done and what was found<br><br>**a) Page 1**<br>**b) Page 2**
**Introduction**		
Background/rationale	2	Explain the scientific background and rationale for the investigation being reported<br><br>**Page 6, line 82**
Objectives	3	State specific objectives, including any prespecified hypotheses<br><br>**Page 7, line 113**
**Methods**		
Study design	4	Present key elements of study design early in the paper<br><br>**Page 8, line 140**
Setting	5	Describe the setting, locations, and relevant dates, including periods of recruitment, exposure, follow-up, and data collection<br><br>**Page 8, line 152**
Participants	6	(*a*) *Cohort study*—Give the eligibility criteria, and the sources and methods of selection of participants. Describe methods of follow-up<br><br>**Page 7, line 123**<br><br>*Case-control study*—Give the eligibility criteria, and the sources and methods of case ascertainment and control selection. Give the rationale for the choice of cases and controls<br>*Cross-sectional study*—Give the eligibility criteria, and the sources and methods of selection of participants
		(*b*) *Cohort study*—For matched studies, give matching criteria and number of exposed and unexposed<br>*Case-control study*—For matched studies, give matching criteria and the number of controls per case
Variables	7	Clearly define all outcomes, exposures, predictors, potential confounders, and effect modifiers. Give diagnostic criteria, if applicable<br><br>Page 7, line 140, Page 8, line 152.

For peer review only

Data sources/ measurement	8*	For each variable of interest, give sources of data and details of methods of assessment (measurement). Describe comparability of assessment methods if there is more than one group<br><br>**Page 8, line 140, Page 8, line 152**
Bias	9	Describe any efforts to address potential sources of bias<br><br>**Page 8, line 154, page 9 line 165**
Study size	10	Explain how the study size was arrived at<br><br>**Page 7, line 127**
Quantitative variables	11	Explain how quantitative variables were handled in the analyses. If applicable, describe which groupings were chosen and why<br><br>**Page 9, line 157**
Statistical methods	12	(*a*) Describe all statistical methods, including those used to control for confounding<br><br>**Page 9, line 170**
		(*b*) Describe any methods used to examine subgroups and interactions<br><br>**Page 9, line 175**
		(*c*) Explain how missing data were addressed<br><br>**Page 9, line 164**
		(*d*) *Cohort study*—If applicable, explain how loss to follow-up was addressed<br><br>**Page 12, line 206**<br><br>*Case-control study*—If applicable, explain how matching of cases and controls was addressed<br>*Cross-sectional study*—If applicable, describe analytical methods taking account of sampling strategy
		(*e*) Describe any sensitivity analyses<br><br>**N/A**

Continued on next page

For peer review only - http://bmjopen.bmj.com/site/about/guidelines.xhtml

**Results**		
Participants	13*	(a) Report numbers of individuals at each stage of study—eg numbers potentially eligible, examined for eligibility, confirmed eligible, included in the study, completing follow-up, and analysed<br><br>**Table 1**
		(b) Give reasons for non-participation at each stage<br><br>**Page 12, line 206**
		(c) Consider use of a flow diagram<br><br>**N/A**
Descriptive data	14*	(a) Give characteristics of study participants (eg demographic, clinical, social) and information on exposures and potential confounders<br><br>**Table 1**
		(b) Indicate number of participants with missing data for each variable of interest<br><br>**Table 1**
		(c) *Cohort study*—Summarise follow-up time (eg, average and total amount)<br><br>**Page 9, line 172**
Outcome data	15*	*Cohort study*—Report numbers of outcome events or summary measures over time<br><br>**Table 1**
		Case-control study—Report numbers in each exposure category, or summary measures of exposure
		Cross-sectional study—Report numbers of outcome events or summary measures
Main results	16	(*a*) Give unadjusted estimates and, if applicable, confounder-adjusted estimates and their precision (eg, 95% confidence interval). Make clear which confounders were adjusted for and why they were included<br><br>**Page 12, line 213 to page 13 line 229**
		(*b*) Report category boundaries when continuous variables were categorized<br><br>**Page 9, line 176**
		(*c*) If relevant, consider translating estimates of relative risk into absolute risk for a meaningful

For peer review only

		time period<br><br>**N/A**
Other analyses	17	Report other analyses done—eg analyses of subgroups and interactions, and sensitivity analyses<br><br>**Page 13, line 234**
**Discussion**		
Key results	18	Summarise key results with reference to study objectives<br><br>**Page 15, line 276**
Limitations	19	Discuss limitations of the study, taking into account sources of potential bias or imprecision. Discuss both direction and magnitude of any potential bias<br><br>**Page 16, line 298**
Interpretation	20	Give a cautious overall interpretation of results considering objectives, limitations, multiplicity of analyses, results from similar studies, and other relevant evidence<br><br>**Page 17, line 334**
Generalisability	21	Discuss the generalisability (external validity) of the study results<br><br>**Page 18, line 344**
**Other information**		
Funding	22	Give the source of funding and the role of the funders for the present study and, if applicable, for the original study on which the present article is based<br><br>**Page 19, line 374**

*Give information separately for cases and controls in case-control studies and, if applicable, for exposed and unexposed groups in cohort and cross-sectional studies.

Note: An Explanation and Elaboration article discusses each checklist item and gives methodological background and published examples of transparent reporting. The STROBE checklist is best used in conjunction with this article (freely available on the Web sites of PLoS Medicine at http://www.plosmedicine.org/, Annals of Internal Medicine at http://www.annals.org/, and Epidemiology at http://www.epidem.com/). Information on the STROBE Initiative is available at www.strobe-statement.org.

For peer review only

For peer review only - http://bmjopen.bmj.com/site/about/guidelines.xhtml

BMJ Open

An engaged research study to assess the effect of a 'real-world' dietary intervention on urinary bisphenol A (BPA) levels in teenagers

Journal:	*BMJ Open*
Manuscript ID	bmjopen-2017-018742.R3
Article Type:	Research
Date Submitted by the Author:	23-Nov-2017
Complete List of Authors:	Galloway, Tamara; University of Exeter, College of Life and Environmental Sciences<br>Baglin, Nigel; Research Projects<br>Lee, Benjamin; University of Exeter Medical School, Institute of Biomedical and Clinical Studies<br>Kocur, Anna; University of Exeter Medical School, Institute of Biomedical and Clinical Studies<br>Shepherd, Maggie; Royal Devon and Exeter National Health Service Foundation Trust, National Institute for Health Research Exeter Clinical Research Facility<br>Steele, Anna; Royal Devon and Exeter National Health Service Foundation Trust, National Institute for Health Research Exeter Clinical Research Facility<br>BPA Schools, Study Consortium; University of Exeter Medical School, Institute of Biomedical and Clinical Studies<br>Harries, Lorna; University of Exeter Medical School, Institute of Biomedical and Clinical Studies
**Primary Subject Heading**:	Public health
Secondary Subject Heading:	Communication
Keywords:	Bisphenol A, Dietary intervention, PUBLIC HEALTH, community, Engaged research

SCHOLARONE™
Manuscripts

An engaged research study to assess the effect of a 'real-world' dietary intervention on urinary bisphenol A (BPA) levels in teenagers

Tamara. S. Galloway[1], Nigel Baglin[2], Benjamin P Lee[3], A.L. Kocur[3], M.H. Shepherd[4], A.M. Steele[4], BPA schools study consortium[5,6,7,8,9,10] and L.W. Harries[3]

[1]*College of Life and Environmental Sciences, University of Exeter, Exeter UK EX4 4AS.*
[2]*Research Projects, Exeter, UK, EX2 5DQ.*
[3]*RNA-Mediated Disease Mechanisms group, Institute of Biomedical and Clinical Sciences, University of Exeter Medical School, University of Exeter, Exeter, UK EX2 5DW.*
[4]*National Institute for Health Research Exeter Clinical Research Facility, Royal Devon and Exeter National Health Service Foundation Trust, and University of Exeter Medical School, Exeter, U.K.*
[5]*Clyst Vale Community College, Broadclyst, Exeter Ex5 3AJ*
[6]*Exeter School, Exeter, Devon, EX2 4NS*
[7]*South Dartmoor Community College, Ashburton, Devon, TQ13 7EW*
[8]*Honiton Community College, Honiton, Devon, EX14 1QT*
[9]*Exeter College, Exeter, Devon, EX4 4JS*
[10]*Exeter Mathematics School, Exeter, Devon, EX4 3PU*

Word count 3021

Correspondence to:

Prof Lorna Harries
University of Exeter Medical School,
Barrack Road,
Exeter, EX2 5DW
Tel =44 1392 406773
Email: L.W.Harries@exeter.ac.uk

Declaration of competing issues

The authors have no competing interests to declare.

For peer review only

ABSTRACT

Objective

Bisphenol A has been associated adverse human health outcomes and exposure to this compound is near-ubiquitous in the Western world. We aimed to examine whether self-moderation of BPA exposure is possible by altering diet in a real-world setting.

Design

An Engaged Research dietary intervention study designed, implemented and analysed by healthy teenagers from 6 schools and undertaken in their own homes.

Participants

A total of 94 students aged between 17 and 19 years from schools in the South West of the UK provided diet diaries and urine samples for analysis.

Intervention

Researcher participants designed a set of literature-informed guidelines for reduction of dietary BPA to be followed for 7 days.

Main outcome measure

Creatinine-adjusted urinary BPA levels were taken before and after the intervention. Information on packaging and food/drink ingested was used to calculate a BPA risk score for anticipated exposure. A qualitative analysis was carried out to identify themes addressing long term sustainability of the diet.

Results

For peer review only

BPA was detected in urine of 86% of participants at baseline at a median value of 1.22 ng/ml (IQR 1.99). No effect of the intervention diet on BPA levels was identified overall ($p = 0.25$), but there was a positive association in those participants who showed a drop in urinary BPA concentration post intervention and their initial BPA level ($p = 0.003$). Qualitative analysis identified themes around feelings of lifestyle restriction and the inadequacy of current labelling practices.

Conclusions

We found no evidence in this self-administered intervention study that it was possible to moderate BPA exposure by diet in a real world setting. Furthermore, our study participants indicated that they would be unlikely to sustain such a diet long term, due to the difficulty in identifying BPA-free foods.

For peer review only

Article Summary

Strengths of the study

- This study represents the largest assessment to date of the potential for moderating one's own BPA exposure through diet
- The study was carried out in a 'real-world' setting rather than a regulated, controlled environment.
- The study was carried out in teenagers, the demographic with amongst the highest exposure.
- Qualitative analysis reveals challenges with sustaining such a diet.

Limitations of the study

- Calculation of a risk score is challenging due to the pervasive nature of BPA contamination

For peer review only

INTRODUCTION

Bisphenol A is one of the world's highest production volume chemicals. It is used in the manufacture of polycarbonate and other plastic consumer products, in heat resistant papers, dental sealants and in the epoxy resin-based lining of food and drink containers [1]. BPA can be found above the detection limit in the urine of the majority of people worldwide [2]. Concern has been raised for public health, since BPA is classified as an endocrine disrupting chemical (EDC) which has been linked with several disorders in cell and animal models [3-5]. Several epidemiological studies have also linked outcomes such as type 2 diabetes, cardiovascular disease, obesity and abnormalities of sex hormone levels with BPA levels in human populations [6-10] Epidemiological data in humans has historically been more contentious however, due to relatively small sample sizes and issues around causality [11]. The Endocrine Society concluded in 2015 that current evidence suggests that BPA and other endocrine disrupting chemicals may have effects on several reproductive, cardiovascular and metabolic traits in humans [12]. The current opinion of food regulatory bodies such as the European Food Standards Agency (EFSA) is that sufficient uncertainty remains to be able to exclude effects on the reproductive, immune, nervous, metabolic and cardiovascular systems and on cancer development [3] whilst the European Chemicals Agency (ECHA) has recently reclassified BPA as a chemical of very high concern due to its endocrine disrupting properties [13].

There has been wide interest in the sources of BPA and the potential for individuals to reduce their own exposure. Human exposure has been reported from inhalation of dust, uptake across the skin from thermal papers and till receipts and release from dental sealants. The main source is the ingestion of food and drink contaminated with BPA leached from

For peer review only

packaging materials [1, 14]. BPA is rapidly metabolised in the gut wall and liver and removed from the blood by the kidneys, with a terminal half-life of 6 hours after oral ingestion [15]. BPA has been detected in food samples packaged in glass, plastic, paper and paperboard cartons, with an average concentration of 0.46 ng/g, rising to over 700 ng/g for certain canned foods. Conversely, in a dietary intervention study in which 22 volunteers consumed a 3 day fresh food diet which excluded canned or packaged foods, there was a 66% reduction in urinary BPA excretion compared to concentrations before the intervention [16]. This latter study involved full dietary replacement of foodstuffs, an approach which is impractical for the population at large. A follow up study found that households who followed written recommendations produced by health care professionals showed no significant change in their BPA exposure [17].

We present an alternative, citizen-science based approach, where 108 student volunteers designed and undertook their own intervention diet, following provision of educational materials. We questioned whether adherence to a self-designed and self-administered ‘real world’ diet over 7 days would lead to significant reductions in excreted urinary BPA, and if so, whether such a diet was likely to be sustainable in the long term.

METHODS

Participant group

We chose adolescents because it has been shown that they have higher concentrations of BPA than adults (aggregated exposure of 1.449 μg/kg body weight per day) [3, 18]. A total of 108 students aged 17-19 from local schools were initially invited to participate in this engaged

For peer review only

research project. Six schools participated in this project (Clyst Vale Community College - 14 students; Exeter School - 12 students; South Dartmoor Community College - 13 students; Honiton Community College - 11 students; Exeter College – 29 students and Exeter Mathematics School – 29 students). Information and samples were available from 94 individuals at both visit one and visit 2 and comprise the complete dataset. This represents the largest intervention study in the population demographic with the one of the highest BPA exposures to date [18]. The number of students invited to participate was based on anticipated effect sizes from previous work of this nature [16], and we allowed for a 10% dropout rate. Students designed all of the materials required for completion of the study (study protocols, food diaries, lifestyle questionnaires, patient information sheets and consent forms (see Supplementary Information files 1 to 6).

Ethical Permission

Ethical permission was granted by the University of Exeter Medical School Ethics Committee (reference number 15/07/074) and the study was carried out in accordance with the Declaration of Helsinki.

The intervention diet

Students designed a "real world" diet designed to reduce consumption of BPA by avoidance of processed foods and foods packaged in known sources of BPA [1, 14]; supplementary information file 1). The study was designed at the University of Exeter as a collaboration

For peer review only

between academic staff and participating students and was developed at a series of interactive workshops attended by all parties.

Students were asked to minimise their intake of known sources of BPA according to a set of guidelines that had been co-designed with them based on the known literature. We requested that calorific intake was maintained as near to their usual diet as possible and recorded details of their daily diet including all food and drink, and its associated packaging, in a self-reported food diary (Supplementary information file 2). Adherence was assessed using a ‘BPA risk score’; each individual dietary item potentially containing BPA was given a score of 1. Heavily processed items were also scored 1 per item. These scores were collated at the end of the 7 day trial to give a final risk score. An example of scores for a single participant on a single day is given in supplementary information file 3. Given the short half-life of BPA, we also carried out a secondary analysis considering only the BPA risk score from the 24 hours immediately preceding the second sample. Information on lifestyle factors including sex, BMI and time of urine collection was also collected (Supplementary information file 4). We recognised that there may be a temptation for students to change their diets before the trial based on their new learning. To avoid this, students were also specifically asked not alter their diet before the intervention.

Sample collection and measurement of urinary BPA

Urine samples were collected into BPA-free bottles (Vacutest Kima, Italy) immediately before and after the intervention, and were frozen at -20˚C within 4 hours. Each participant was sampled twice, once at visit 1 before the intervention and once at visit 2 after the

For peer review only

intervention. Sample collections were staggered to allow for the large number of participants passing through the facility, but students were sampled during the same time slot at both visits to account for circadian variation in BPA metabolism. The initial samples were collected during the early part of the day just prior to the students commencing the trial. The second samples were taken over the same time period 7 days later, just prior to the students recommencing their usual diet. Samples were transported on dry ice to a commercial laboratory (Rovaltain Research Company, Aixain, France) where analysis of total BPA was assessed by gas chromatography-tandem mass spectrometry. Experimental methods were validated for linearity, detection limit and accuracy and specificity of quantification based on the Standard NF T 90-201 for determination of xenobiotics. A quality control check of known standards injected every 6 samples at two levels of concentration (0.5 ng/ml and 5 ng/ml) was quantified with each batch of unknown samples. Water-only samples were included as negative controls. Urinary creatinine was measured at the Royal Devon and Exeter Hospital using the Jaffe method on the Roche P800 platform (Roche, Mannheim, Germany), to allow correction for urine dilution. Results were expressed as a BPA:creatinine ratio. Samples where BPA was detected but quantifying at or around the limits of quantification (LoQ) of 0.1ng/ml were scored as LoQ/√2 according to the method of Hornung and Reed [19].

Statistical Analysis

The difference between urinary BPA adjusted for creatinine between samples taken at visits 1 and 2 was assessed to generate a ΔBPA continuous variable. BPA risk scores were calculated as a continuous variable. The relationship between urinary BPA levels before and after the 7 day intervention was assessed using a repeated-measure ANOVA, adjusted for sex, time of

For peer review only

sampling and BMI, with and without correction for creatinine. The relationship between urinary BPA at visit 1 and whether or not the participants had lower BPA at visit 2 was also examined by binary logistic regression, adjusted for sex, time of sampling and BMI. Here, samples showing small changes < 0.5ng/ml in either direction were omitted to avoid natural stoichiometric variation around zero. The relationship between change in BPA (ΔBPA) and BPA risk score was assessed by linear regression, adjusted for sex, time of sampling and BMI both with and without adjustment for creatinine. Statistical analysis was carried out using SPSS, v.22 (IBM, USA).

Impact of following reduced BPA diet on lifestyle

We carried out quantitative and qualitative analysis to address long-term sustainability of the diet. Data on the impact of following the diet on feelings of dietary restriction, time spent sourcing or preparing meals, calorific intake and long term sustainability were collected via a questionnaire (See Supplementary information file 4). The questionnaire also included a freeform section where participants could write about their experiences following the diet in a non-prescribed fashion for qualitative analysis. Qualitative data was assessed for thematic content by two experienced qualitative researchers. Key themes were independently identified and coded until agreement was reached.

RESULTS

Participant Characteristics

For peer review only

There were 108 volunteer participants invited to participate in this engaged research study. A small number were absent or unable to produce a urine sample at both visits. A complete dataset was thus received from 94 students. Information on the characteristics of the study cohort are given in table 1.

Table 1: Characteristics of the study population. A complete dataset was available on 94 out of 108 participants. IQR = interquartile range, SD = Standard deviation. The units of BPA are ng/ml and BMI is defined as Kg/m^2. LoQ = limit of quantification. Urinary BPA levels are given both as unadjusted data and as a BPA (ng/ml) to creatinine (mg/ml) ratio.

Unadjusted urinary BPA at visit 1 (n = 94)	
median (IQR)	1.01 (2.01)
95% confidence intervals	1.19 to 2.57
mean (SD)	1.88 (2.68)
Number of samples below LoQ (0.1ng/ml)	15
Minimum value (ng/ml)	0.07
Maximum value (ng/ml)	13.55
Creatinine-adjusted urinary BPA at visit 1 (n = 94)	
median (IQR)	1.22 (1.99)
95% confidence intervals	1.16 to 1.20
mean (SD)	1.58 (1.64)
Number of samples below LoQ (0.1ng/ml)	15
Minimum value (ng/ml)	0.05
Maximum value (ng/ml)	8.56
Unadjusted urinary BPA at visit 2 (n = 94)	
median (IQR)	1.47 (2.87)
95% confidence intervals	1.59 to 3.97
mean (SD)	2.78 (4.64)
Number of samples below LoQ (0.1ng/ml)	12
Minimum value (ng/ml)	0.07
Maximum value (ng/ml)	31.2
Creatinine-adjusted urinary BPA at visit 2 (n = 94)	
median (IQR)	1.24 (2.51)
95% confidence intervals	1.21 to 5.01
mean (SD)	3.13 (7.36)
Number of samples below LoQ (0.1ng/ml)	12
Minimum value (ng/ml)	0.04
Maximum value (ng/ml)	53.42
Unadjusted ΔBPA (n = 94)	

For peer review only

median (IQR)	0.06 (2.09)
95% confidence intervals	0.05 to 1.75
mean (SD)	0.90 (3.32)
Minimum value	-6.42
Maximum value	17.64
Adjusted ΔBPA (n = 94)	
median (IQR)	0.05 (2.94)
95% confidence intervals	-0.28 to 3.39
mean (SD)	1.55 (7.16)
Minimum value	-4.47
Maximum value	50.38
BPA risk score (n = 94)	
median (IQR)	17.0 (11.0)
95% confidence intervals	15.4 to 18.8
mean (SD)	17.1 (6.63)
Demographics (n= 94)	
Sex - % male	44
Exposure to estrogens - % of cohort	15
BMI– median (IQR)	20.7 (3.43)
BMI– mean (SD)	21.3 (3.13)

BPA was detected in the urine of 86% of subjects at visit 1 prior to the intervention. Missing samples were due to non-attendance of participants or non-provision of a suitable sample. Samples below the limit of quantification were scored as 0.07 ng/ml (LoQ/√2).

Creatinine-adjusted urinary BPA concentrations do not change significantly after following an intervention diet designed to reduce BPA exposure for 7 days.

The median change in creatinine-adjusted urinary BPA between visits (ΔBPA) was 0.05 ng/ml with an interquartile range of 2.94 ng/ml. We identified no changes in urinary BPA between visits ($p = 0.25$; figure 1a). Three outliers with very high urinary BPA readings at visit 2 were excluded from the analysis, since these samples lay outside the linear range of analysis, so confidence in quantification was poor. No confounding factors included in the analysis were associated with change in BPA ($p = 0.78$, 0.43 and 0.36 for sex, time of sample collection and BMI respectively). We also identified no change in BPA levels between visits

For peer review only

using data uncorrected for creatinine ($p = 0.20$). We also assessed whether participants from different schools showed variable BPA levels at either visit 1, or change in BPA, but no such effects were noted.

Similarly, no relationship between change in urinary BPA (ΔBPA) and BPA risk score was identified (beta coefficient 0.08, standard error 0.07, $p = 0.55$; figure 1b). No associations were noted between change in urinary BPA and BPA risk score in data not adjusted for creatinine ($p = 0.27$). We found no association between ΔBPA and BPA risk score when considering only the exposure on the day prior to testing, taking into account the short half-life of BPA ($p = 0.16$ and $p = 0.33$ for adjusted and unadjusted data respectively).

Participants with highest starting urinary BPA levels were more likely to demonstrate lower BPA levels at visit 2.

We found an inverse relationship between initial BPA levels and whether a participant had reduced BPA levels at visit 2 ($p = 0.003$). These data indicate that the participants in the cohort with the highest creatinine-adjusted urinary BPA levels at visit 1 were more likely to demonstrate a drop in their urinary BPA at visit 2 (figure 2).

Following the intervention diet has significant effects on participant lifestyle

Participants indicated that following the diet had no significant cost implications on family finances, with 50% of participants reporting that it had cost more, and 50% reporting that

For peer review only

costs had decreased or remained the same. Although participants did not spend longer preparing their food, 78% of participants reported that their shopping took longer. Calorific intake was not affected for the majority of participants (58%) of participants. A large percentage of the cohort (91%) reported that they felt at least slightly restricted in their food choices and 27% of participants reported that they felt very restricted. Finally, 66% of participants stated that they would find it hard or very hard to follow the diet long term.

Qualitative analysis of the effect of following the diet on lifestyle

We identified 5 overriding themes in our qualitative analysis of the effect of following the diet on lifestyle. These were 1) the widespread use of plastics possibly containing BPA in food packaging ("almost everything is packaged in plastic" – participant 70, "Literally everything involved plastic" – participant 28). 2) Lack of clarity in labelling of products and packaging potentially containing BPA ("I found it really hard to know what foods I could eat … there is never a guarantee it is BPA free" – participant 43, " The biggest problem was that a lot of packaging doesn't state what type of plastic it is or whether it contains BPA" – participant 74). 3) The perceived restrictions of being on the 'real world' BPA avoidance diet ("Difficulty eating out, hard to find foods in college or 'out' that hadn't touched BPA. My family had a takeaway on Saturday night and I couldn't eat it" – participant 56, "Sometimes I can't eat / drink what I want because of the recycling number" – participant 112). 4) The impact of eating 'BPA free' was the only positive theme emerging ("I feel I have eaten much more healthily this week … I didn't eat so much junk food" – participant 74, "I ate more vegetables and less chocolate" – participant 83). 5) The impact on shopping habits ("You can't get it all from supermarkets" – Participant 37; "Had to go to more individual food shops" – participant 103).

For peer review only

DISCUSSION

Exposure to the endocrine disrupting chemical Bisphenol A (BPA) is ubiquitous [2], with growing evidence that it may be associated with adverse health outcomes [4]. Here, 94 researcher participants aged 17-19 years designed and undertook a quantitative and qualitative engaged research project designed to assess the potential for reduction of personal exposure to BPA through moderation of diet, which would have utility in a ‘real world’ setting. We conclude that the ‘real world’ diet designed to reduce BPA exposure had no effect on creatinine-adjusted urinary BPA concentrations in our cohort over a period of 7 days in our dataset.

Although levels of urinary BPA in our study cohort were slightly lower at the outset of the study in our cohort than in others [18], measureable levels were present in the vast majority of our participants. Participants were unable to achieve a reduction in their urinary BPA levels over the 7 day trial period, despite good compliance to supplied guidelines. Avoidance of BPA was not easily achieved on an individual level in our study population, with qualitative analysis indicating that participants experienced feelings of restriction and difficulties in sourcing BPA-free food due to inadequate labelling of foods and food packaging. This suggests that the intervention would be difficult to sustain in the longer term.

This work represents the largest group of unrelated participants in a high exposure demographic_to date, since previous work has focused on families and related individuals [16] [17], who may share common sources of BPA. Although other population demographics

For peer review only

such as young children may have higher levels of BPA than our chosen study population [18], it would not have been possible to do the sort of engaged research project that we envisaged in this group. Our intervention is a 'real world' diet, designed to a set of guidelines (such as reduction in the usage of tinned foods or foods with high levels of processing), rather than the strict, prescribed diets that have been used in other studies [16], which suggested that it was possible for participants to reduce their urinary BPA excretion by approximately 60% in a period of just 3 days [16]. In our self-designed, self-administered study this was unachievable. This may reflect the difficulty in identifying and sourcing foods free of BPA in our commercial environment. Finally, the qualitative thematic analysis we carried out in our study has given an indication that adherence to even a 'real world' BPA reduction diet with fewer restrictions and more choice over the longer term was unlikely in our study population due to difficulties in identifying foodstuffs likely to contain less BPA.

BPA has a terminal half-life of 6 hours [15]. Spot samples may therefore not be as accurate as continuous sampling strategies (24hr urine collection). However, recent studies suggest that despite its short half-life, measureable BPA remains present for up to 43 hours post-fasting, indicating non-food exposures or accumulation in body tissues such as fat [20]. We identified no impact of time of sample collection on BPA levels in our sample set, in either creatinine-adjusted or unadjusted data, indicating that our measurements were not influenced by time since the last meal. Spot sampling as used here may therefore represent an acceptable compromise and remains a practical option in the community setting of our study. The large variability in urinary BPA levels within an individual sampled at different times may also have reduced our ability to observe an effect. This could be facilitated by the use of multiple sampling, or pools of multiple urines, but was not feasible within the confines of our study.

For peer review only

Calculating an accurate BPA risk score is challenging. Data were self-reported, and foodstuffs are not labelled for BPA content. It is difficult to generalise across food types and large variations in BPA concentrations occur between different products of the same food type or even different lots of the same product [1]. Foods that were free of BPA-containing packaging (as far as it was possible to tell) may have been highly processed or contain food items from a variety of sources. Highly processed and 'fast' food has previously been demonstrated to be a source of BPA [21]. A study of the temporal trends seen in composite food samples found no change in the overall BPA content of the food, despite large reduction in the BPA content of some individual food items, illustrating the difficulties in effectively excluding BPA from a varied diet [22]. Participants may therefore have changed BPA containing foods for other, perceived healthier choices, which may still contain BPA by virtue of processing.

BPA enters foodstuffs by leaching from polycarbonate or epoxy resin after manufacture, or by hydrolysis of the polymer itself [23]. The migration rate of BPA increases with higher temperatures [24], and with time and use, e.g. repeated use of polycarbonate water bottles [25]. Exposure to BPA can also occur through routes other than food, including dust ingestion and dermal absorption [26] and this was not taken into account in our study. A study of volunteers who purposefully handled thermal receipts showed an increase in urinary BPA excretion of up to 84%, and their BPA levels took longer to return to pre exposure levels, suggesting a difference in the bio-availability of BPA through skin and oral routes [27]. It is also possible that some manufacturers may have voluntarily reduced the amount of BPA-containing food packaging compared to their previous usage, given the attention that

For peer review only

endocrine disrupting chemicals have received in the media. However, measurable levels of BPA were still detected in the majority of participants in our study, which suggests that there may be other, non-dietary, sources of BPA, and that exposure to BPA remains an issue. We may also have been underpowered to detect subtle changes in urinary BPA, given the heterogeneity in food choice; detection of such effects may need thousands of participants. Finally, our study, like other studies of its type, does not take account of inter-individual differences in the metabolism and excretion of BPA arising from differences in genetic background between people. BPA is metabolised primarily by UDP-glucuronosyltransferases, and altered activity polymorphisms of these enzymes have been reported [28].

Emerging evidence suggests that that BPA may be linked to several chronic human health conditions [6-9, 29], suggesting that continued study of the human health effects of BPA exposure is justified. The opinion of the European Food Safety Authority (EFSA), is that whilst uncertainty over the human health effects of BPA exists, caution should be exercised in ingestion of BPA [3]. Our data suggests that in our study population, it is unlikely that participants could moderate their own BPA exposure in the long term by self-directed modification of diet in a ‘real world’ setting, and furthermore, participants would have been reluctant to adopt such a lifestyle change in the longer term due to the restrictions in dietary choice and the effects on day to day life. Most of these barriers appear to arise from the pervasiveness of BPA in our food chain, and inadequate labelling of foods packaged in BPA-containing substances. We propose that until a definitive assessment of the health risks of BPA is available, informed choice over whether or not to consume BPA and similar chemicals in foodstuffs should be facilitated by better labelling.

For peer review only

CONTRIBUTORSHIP STATEMENT

TSG - Contributed to study design and co-wrote the paper

NB - Contributed to study design and participant involvement

BP - Managed the technical aspects of the project and reviewed the manuscript

ALK - Contributed to data entry and interpretation and reviewed the manuscript

BPA Schools Study Consortium members - designed and interpreted the study and contributed to the manuscript.

MHS - Carried out the qualitative analysis and reviewed the manuscript

AMS - Managed sample collection, contributed to study design and reviewed the manuscript.

LWH - PI, managed the study, wrote the manuscript

ACKNOWLEDGEMENTS

We acknowledge the work of the NIHR Exeter Clinical Research Facility in aiding the collection of the urine samples.

COMPETING INTERESTS

The authors have no competing interests to declare.

For peer review only

DATA SHARING STATEMENT

Data are available upon reasonable request by emailing Lorna Harries (L.W.Harries@exeter.ac.uk).

FUNDING

This study was funded by a Wellcome Trust People Award to LWH and TG (grant number 105162/Z/14/Z).

REFERENCES

1 Geens T, Aerts D, Berthot C*, et al.* A review of dietary and non-dietary exposure to bisphenol-A. *Food Chem Toxicol* 2012;**50**:3725-40.

2 WHO. World Health Organisation Background paper on mechanisms of action of bisphenol A and other biochemical/molecular interactions. *WHO/HSE/FOS* 2010;**11.1**.

3 EFSA. Scientific opinion on the risks to public health related to the presence of bisphenol A (BPA) in foodstuffs. *EFSA journal* 2015;**13**.

4 Rochester JR. Bisphenol A and human health: a review of the literature. *Reprod Toxicol* 2013;**42**:132-55.

For peer review only

5 WHO. Joint FAO/WHO Expert meeting to review toxicological and health aspects of bisphenol A Summary report. 2010.

6 Galloway T, Cipelli R, Guralnik J*, et al.* Daily bisphenol A excretion and associations with sex hormone concentrations: results from the InCHIANTI adult population study. *Environ Health Perspect* 2010;**118**:1603-8.

7 Melzer D, Rice NE, Lewis C*, et al.* Association of urinary bisphenol a concentration with heart disease: evidence from NHANES 2003/06. *PLoS One* 2010;**5**:e8673.

8 Song Y, Chou EL, Baecker A*, et al.* Endocrine-disrupting chemicals, risk of type 2 diabetes, and diabetes-related metabolic traits: A systematic review and meta-analysis. *J Diabetes* 2015.

9 Savastano S, Tarantino G, D'Esposito V*, et al.* Bisphenol-A plasma levels are related to inflammatory markers, visceral obesity and insulin-resistance: a cross-sectional study on adult male population. *J Transl Med* 2015;**13**:169.

10 Braun JM. Early-life exposure to EDCs: role in childhood obesity and neurodevelopment. *Nat Rev Endocrinol* 2017;**13**:161-73.

11 Vandenberg LN, Chahoud I, Heindel JJ*, et al.* Urinary, circulating, and tissue biomonitoring studies indicate widespread exposure to bisphenol A. *Environ Health Perspect* 2010;**118**:1055-70.

12 Gore AC, Chappell VA, Fenton SE*, et al.* EDC-2: The Endocrine Society's Second Scientific Statement on Endocrine-Disrupting Chemicals. *Endocr Rev* 2015;**36**:E1-E150.

For peer review only

13 Agency EC. AGREEMENT OF THE MEMBER STATE COMMITTEE ON THE IDENTIFICATION OF 4,4'-ISOPROPYLIDENEDIPHENOL (BISPHENOL A) AS A SUBSTANCE OF VERY HIGH CONCERN 2017.

14 Lakind JS, Naiman DQ. Daily intake of bisphenol A and potential sources of exposure: 2005-2006 National Health and Nutrition Examination Survey. *Journal of exposure science & environmental epidemiology* 2011;**21**:272-9.

15 Dekant W, Volkel W. Human exposure to bisphenol A by biomonitoring: methods, results and assessment of environmental exposures. *Toxicol Appl Pharmacol* 2008;**228**:114-34.

16 Rudel RA, Gray JM, Engel CL*, et al.* Food packaging and bisphenol A and bis(2-ethyhexyl) phthalate exposure: findings from a dietary intervention. *Environ Health Perspect* 2011;**119**:914-20.

17 Sathyanarayana S, Alcedo G, Saelens BE*, et al.* Unexpected results in a randomized dietary trial to reduce phthalate and bisphenol A exposures. *Journal of exposure science & environmental epidemiology* 2013;**23**:378-84.

18 Calafat AM, Ye X, Wong LY*, et al.* Exposure of the U.S. population to bisphenol A and 4-tertiary-octylphenol: 2003-2004. *Environ Health Perspect* 2008;**116**:39-44.

19 Hornung R, Reed L. Estimation of average concentration in the presence of nondetectable values. *Appl Occupat Environ Hyg* 1990;**5**:46–51.

For peer review only

20 Stahlhut RW, Welshons WV, Swan SH. Bisphenol A data in NHANES suggest longer than expected half-life, substantial nonfood exposure, or both. *Environ Health Perspect* 2009;**117**:784-9.

21 Zota AR, Phillips CA, Mitro SD. Recent Fast Food Consumption and Bisphenol A and Phthalates Exposures among the U.S. Population in NHANES, 2003-2010. *Environ Health Perspect* 2016;**124**:1521-8.

22 Cao XL, Perez-Locas C, Robichaud A*, et al.* Levels and temporal trend of bisphenol A in composite food samples from Canadian Total Diet Study 2008-2012. *Food Addit Contam Part A Chem Anal Control Expo Risk Assess* 2015;**32**:2154-60.

23 Aschberger K, Castello P, Hoekstra E*, et al.* Bisphenol A and baby bottles: challenges and perspectives. 2010.

24 Le HH, Carlson EM, Chua JP*, et al.* Bisphenol A is released from polycarbonate drinking bottles and mimics the neurotoxic actions of estrogen in developing cerebellar neurons. *Toxicol Lett* 2008;**176**:149-56.

25 Brede C, Fjeldal P, Skjevrak I*, et al.* Increased migration levels of bisphenol A from polycarbonate baby bottles after dishwashing, boiling and brushing. *Food Addit Contam* 2003;**20**:684-9.

26 Myridakis A, Chalkiadaki G, Fotou M*, et al.* Exposure of Preschool-Age Greek Children (RHEA Cohort) to Bisphenol A, Parabens, Phthalates, and Organophosphates. *Environ Sci Technol* 2016;**50**:932-41.

For peer review only

27 Lv Y, Lu S, Dai Y*, et al.* Higher dermal exposure of cashiers to BPA and its association with DNA oxidative damage. *Environ Int* 2017;**98**:69-74.

28 Stingl JC, Bartels H, Viviani R*, et al.* Relevance of UDP-glucuronosyltransferase polymorphisms for drug dosing: A quantitative systematic review. *Pharmacol Ther* 2014;**141**:92-116.

29 Melzer D, Osborne NJ, Henley WE*, et al.* Urinary bisphenol A concentration and risk of future coronary artery disease in apparently healthy men and women. *Circulation* 2012;**125**:1482-90.

For peer review only

FIGURE LEGENDS

Figure 1. The effect of a 'real world' BPA avoidance diet on urinary BPA exposure over a 7 day period. **A**. Urinary BPA levels (ng/ml) adjusted for urinary creatinine were plotted at visit 1 before the intervention and at visit 2 after the intervention. The 3 extreme outliers have been removed. The trajectories of individual participant measurements are shown. **B**. Change in urinary BPA levels in ng/ml following the intervention diet are plotted against the self-reported BPA risk score.

Figure 2. The effect of baseline urinary BPA on the probability of achieving a drop in levels following the intervention. This graph illustrates the median urinary BPA level adjusted for creatinine at visit 1 prior to the intervention expressed relative to whether or not a reduction in urinary BPA levels was achieved following the 7 day intervention diet at visit 2. Error bars refer to the interquartile range of measurement.

For peer review only

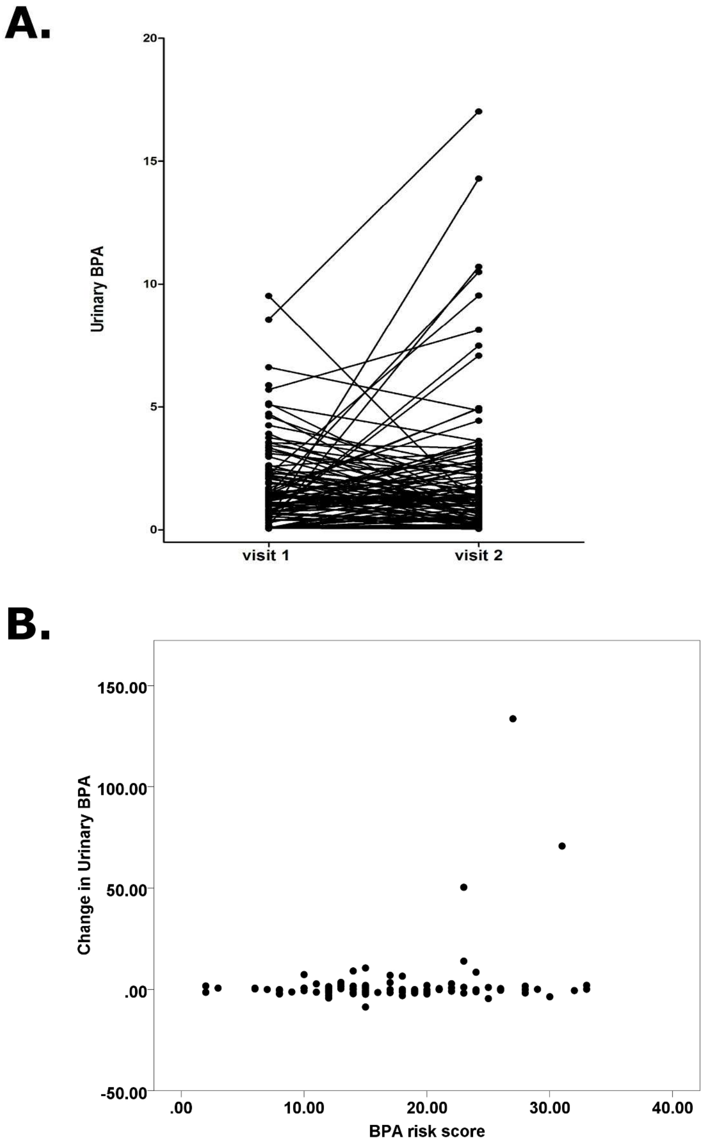

Figure 1

209x297mm (300 x 300 DPI)

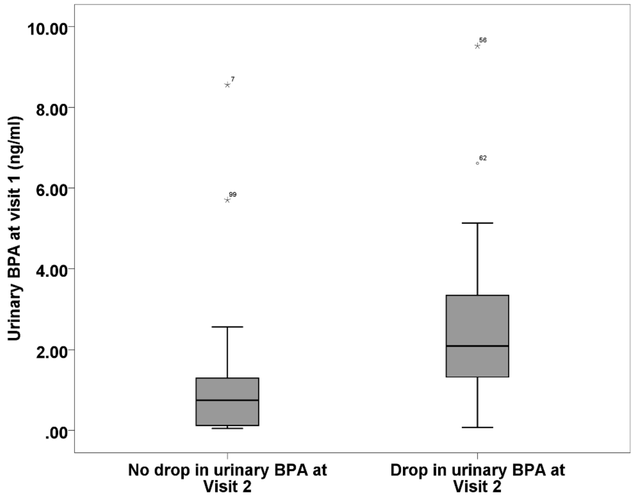

Figure 2

209x297mm (300 x 300 DPI)

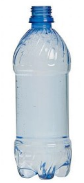

BPA: Myth and Reality
Dietary Intervention Guidelines

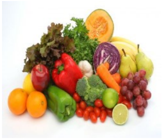

General instructions

The purpose of this dietary intervention trial is to follow a diet designed to minimise routes of exposure to the food packaging chemical bisphenol A (BPA). For the dietary intervention period, please follow as closely as possible the instruction given below. Try to maintain your diet during the intervention period to be as closely similar to your normal diet as possible, in terms of the content, amount and calorific value of the food you eat. Please record details of each meal and the drinks and snacks you consume on the forms provided. Below are some general cooking and eating tips and an indication of which foods are best to avoid and those that are considered a low source of BPA.

Cooking and eating tips for the intervention period.

The general approach is to replace any food items that fall into the ‘avoidance’ category with an alternative, chosen to minimise exposure to BPA

- **Switch to stainless steel and glass food storage and drink containers.**
- **Move foods to ceramic or glass food containers before microwaving.**
- **Consider a coffee filter or percolator for coffee – home coffee makers (Such as Nespresso^{TM}) may have polycarbonate-based water tanks and phthalate-based tubing.**
- **Eat out less, especially at restaurants that do not use fresh ingredients.**
- **Avoid canned food consumption. Where possible, replace with fresh produce or cardboard or tetrapack packaged alternatives.**
- **Choose fresh fruits and vegetables when possible, and frozen if not.**
- **Soak dried beans for cooking rather than tinned.**

For peer review only

Foods to avoid

Tinned foods. Top ten tinned foods that are reported to be sources of BPA include coconut milk, soup, meat, vegetables, meals (e.g. pasta with sauce), juice, fish, beans, meal replacement drinks, fruit.

Carbonated/fizzy drinks and juices in cans. Avoid carbonated drinks in cans and drinks stored for prolonged periods in reusable sports bottles, unless they are labelled 'BPA free' (many commercial sports bottles are).

Fast food from commercial outlets. Most processed food has passed through numerous processes, and each additional processing step provides an opportunity for BPA to enter through packaging or tubing. Try to replace fast and processed foods with a freshly prepared and cooked alternative.

Packaged fruit and vegetables. Replace these where possible with unpackaged, loose fruit and vegetable items as far as possible.

Convenience/ready meals. Plastics types considered safest in terms of chemical migration are recycling numbers 2 and 5. Avoid food prepared in packaging with recycling number 7, which includes many different types of polymer and mixed polymers, including polycarbonate, a source of BPA. Try to avoid foods that are designed to be heated in the microwave in their packaging.

Chocolate and ice cream. Individuals who report eating chocolate bars and ice cream on a regular basis have been reported to have higher than average BPA exposure. Try to avoid excessive consumption.

Non-food or food packaging routes of exposure

Although plastics found in consumer goods such as DVDs, CDs, computer goods and sunglasses do contain BPA, this is not an important route of exposure.

Till receipts often contain high levels of BPA, so wash your hands before eating or drinking if you have been handling them.

Dental sealants may contain BPA, so avoid any pre-planned dental work

Example daily diet

Food Item	Comments
**Breakfast**	
Cereal, Fruit	
Milk	Polypropylene or glass packaging
Bread	
Yoghurt	Choose polypropylene container
**Lunch**	
Meat or fish products	Check packaging and avoid those labelled no. 7. Avoid tinned ingredients
Cheese	
Salad items, Fruit	Choose unpackaged where possible, wash before use
Pasta	
**Dinner**	
Shepherds pie	Cooked in saucepan and oven rather than microwaved in plastic
Green beans	Fresh or frozen
Bread	
**Drinks**	
Water	Water direct from tap or use stainless steel or BPA free water bottle
Tea/coffee	Prepare in teapot or cafetiere, avoid commercial coffee makers
Carbonated drinks	Avoid canned drinks and those stored in reusable containers for prolonged periods
Milk	Polypropylene or glass packaging
**Snacks**	
Fruit	
Potato crisps	

For peer review only

Place participant barcode here	FOOD - DAY 1		DRINK – DAY 1	
**Contents of meal**	Was food packaged in plastic known or suspected to contain BPA?	Was food cooked in plastic known or suspected to contain BPA?	**Type of Drink**	Packaging (please describe) e.g. canned, plastic (Δ number), tetrapak
**Breakfast:**				
1.				
2.				
3.				
4.				
5.				
**Lunch**:				
1.				
2.				
3.				
4.				
5.				
**Dinner**:				
1.				
2.				
3.				
4.				
5.				
**Snacks**:				
1.				
2.				
3.				
4.				

For peer review only

Place participant barcode here	FOOD - DAY 2		DRINK – DAY 2	
**Contents of meal**	Was food packaged in plastic known or suspected to contain BPA?	Was food cooked in plastic known or suspected to contain BPA?	**Type of Drink**	Packaging (please describe) e.g. canned, plastic (Δ number), tetrapak
**Breakfast:**				
1.				
2.				
3.				
4.				
5.				
**Lunch**:				
1.				
2.				
3.				
4.				
5.				
**Dinner**:				
1.				
2.				
3.				
4.				
5.				
**Snacks**:				
1.				
2.				
3.				
4.				

For peer review only

Place participant barcode here	FOOD - DAY 3		DRINK – DAY 3	
**Contents of meal**	Was food packaged in plastic known or suspected to contain BPA?	Was food cooked in plastic known or suspected to contain BPA?	**Type of Drink**	Packaging (please describe) e.g. canned, plastic (Δ number), tetrapak
**Breakfast:**				
1.				
2.				
3.				
4.				
5.				
**Lunch**:				
1.				
2.				
3.				
4.				
5.				
**Dinner**:				
1.				
2.				
3.				
4.				
5.				
**Snacks**:				
1.				
2.				
3.				
4.				

For peer review only

Place participant barcode here	FOOD - DAY 4		DRINK – DAY 4	
**Contents of meal**	Was food packaged in plastic known or suspected to contain BPA?	Was food cooked in plastic known or suspected to contain BPA?	**Type of Drink**	Packaging (please describe) e.g. canned, plastic (Δ number), tetrapak
**Breakfast:**				
1.				
2.				
3.				
4.				
5.				
**Lunch**:				
1.				
2.				
3.				
4.				
5.				
**Dinner**:				
1.				
2.				
3.				
4.				
5.				
**Snacks**:				
1.				
2.				
3.				
4.				

For peer review only

Place participant barcode here	FOOD - DAY 5		DRINK – DAY 5	
**Contents of meal**	Was food packaged in plastic known or suspected to contain BPA?	Was food cooked in plastic known or suspected to contain BPA?	**Type of Drink**	Packaging (please describe) e.g. canned, plastic (Δ number), tetrapak
**Breakfast:**				
1.				
2.				
3.				
4.				
5.				
**Lunch**:				
1.				
2.				
3.				
4.				
5.				
**Dinner**:				
1.				
2.				
3.				
4.				
5.				
**Snacks**:				
1.				
2.				
3.				
4.				

For peer review only

Place participant barcode here	FOOD - DAY 6		DRINK – DAY 6	
**Contents of meal**	Was food packaged in plastic known or suspected to contain BPA?	Was food cooked in plastic known or suspected to contain BPA?	**Type of Drink**	Packaging (please describe) e.g. canned, plastic (Δ number), tetrapak
**Breakfast:**				
1.				
2.				
3.				
4.				
5.				
**Lunch**:				
1.				
2.				
3.				
4.				
5.				
**Dinner**:				
1.				
2.				
3.				
4.				
5.				
**Snacks**:				
1.				
2.				
3.				
4.				

For peer review only

Place participant barcode here	FOOD - DAY 7		DRINK – DAY 7	
**Contents of meal**	Was food packaged in plastic known or suspected to contain BPA?	Was food cooked in plastic known or suspected to contain BPA?	**Type of Drink**	Packaging (please describe) e.g. canned, plastic (Δ number), tetrapak
**Breakfast:**				
1.				
2.				
3.				
4.				
5.				
**Lunch**:				
1.				
2.				
3.				
4.				
5.				
**Dinner**:				
1.				
2.				
3.				
4.				
5.				
**Snacks**:				
1.				
2.				
3.				
4.				

For peer review only

Participant barcode

Additional study information.

Please do not feel obliged to answer these questions if you are uncomfortable doing so.

Gender

☐ Female
☐ Male
☐ Prefer not to say

Tobacco Usage – Have you used tobacco over the past week

☐ Yes
If so, what type and how much?____
☐ No
☐ Prefer not to say

Alcohol Usage – Have you used alcohol over the past week

☐ Yes
If so, what type and how much?____
☐ No
☐ Prefer not to say

Medication- Have you taken any medication over the last week?

☐ Yes
☐ No
☐ Prefer not to say

If so, Please name the medication______________________ ☐ Prefer not to say

Vegetarian/vegan diet - Have you eaten or drank any soya products over the past week?

☐ Yes
☐ No
☐ Prefer not to say

Your measurements - leave blank if you prefer not to say

Your height ____________________________

Your weight____________________________

For peer review only

Supplementary information file 3. Example daily diet diary. A score of 1 is given to each item containing suspected to contain BPA or be packaged in BPA-containing materials. Highly processed foods are also scored as 1, due to uncertainties in the processing procedures. The daily totals are summed to produce a BPA risk score for the 7 day intervention.

	**Item**	**Packaging**	**Score**
**Breakfast**			
	Homemade pancakes	None	0
	Sugar	None	0
	lemon	None	0
	milk	HDPE	0
**Lunch**			
	Homemade Cheese sandwich	none	0
	Homemade sultana cake	none	0
	water	glass	0
**Dinner**			
	Homemade omelette	none	0
	Sweetcorn	Can	1
	Rice	Cellophane	0
	Tomatoes	none	0
	water	glass	0
**Snacks**			
	Crisps (processed)	Cellophane	1
	Apple	none	0
	milk	HDPE	0
		**Total for day**	**2**

For peer review only

Participant Barcode

BPA: Myth and Reality diet questionnaire

1. Were there any times during the week that you knowingly/unknowingly did not stick to the diet? Please tick any that apply and give indication of frequency.

School meals ☐ _____ times

Restaurants/cafés ☐ _____ times

Friends' houses ☐ _____ times

Takeaway ☐ _____ times

Other ______________________________ ☐ _____ times

For peer review only

2. If you heated your food in a microwave, what was the food in? Tick any which apply and give indication of frequency.

A food storage container or bowl known or suspected to contain BPA ☐_____times

3. When you or your family drank water, where did your water come from? Tick any which apply and give indication of frequency.

Plastic filter jug known or suspected to contain BPA ☐_____times

Individual water bottle known or suspected to contain BPA ☐_____times

Larger water container known or suspected to contain BPA ☐_____times

4. How many times during the week did you eat food that had been stored or transported in plastic containers known or suspected to contain BPA?

____

5. How many times during the week did you eat tinned food or drink from cans?

____

6. Did the BPA reduced diet affect How much you spent on shopping?

Spent more ☐

Spent less ☐

No difference ☐

Participant questionnaire V3 25Jun15

Participant Barcode

7. Did it take longer to source your food than usual?

Yes ☐ No ☐

If so, why? ______________________________

____

8. Did it take longer to prepare food than usual?

Yes ☐ No ☐

If so, why? ______________________________

____

9. How restricted did you feel by your food choice?

☐ Very ☐ Slightly ☐ No difference

If you felt you were restricted by the diet, why was this? ______________________________

____

10. Did the diet affect your calorific intake?

Yes ☐ No ☐

If so, why? ______________________________

____

For peer review only - http://bmjopen.bmj.com/site/about/guidelines.xhtml

For peer review only

Participant Barcode

11. How easy would you find it to sustain this diet over a longer period of time?

Very easy ☐

Easy ☐

Hard ☐

Very hard ☐

Not sure ☐

12. Is there anything else about following the diet that you would like to add?

For peer review only - http://bmjopen.bmj.com/site/about/guidelines.xhtml

Thank you for reading this leaflet. If you wish to participate in this study, you will be asked to agree to the consent statements below in the presence of a member of the research team.

CONSENT STATEMENTS

1. I confirm that I have read this information sheet and have discussed participation in this project with my family. I have had opportunity to consider the information, ask questions and have had these questions answered satisfactorily. *You should not give consent until you are happy that you understand what the study involves.*

2. I understand that my participation is voluntary and that I am free to withdraw at any time without giving any reason, without my right to participate in the rest of the study being affected. *This means that even if you helped design this study you do not have to be a participant and you should not feel under any pressure to participate.*

3. I agree to participate in this study as a research subject. *This means that you agree to participate in a one-week diet and to provide two blood and urine samples.*

4. I understand that my anonymised blood and urine samples and linked anonymous questionnaire data will be sent to University of Exeter Medical School, Royal Devon & Exeter Hospital and my urine sample only will be sent to the Rolvaltain laboratory, a specialist BPA analysis company. *This means that laboratory staff will not know that samples belong to you, but dedicated staff at the University of Exeter, with training and experience in data protection ,will be able to link your sample data to your questionnaire data.*

5. I understand that RNA (genetic material) will be extracted from my blood and will be stored anonymously. *This means that Professor Harries' team will use our RNA to provide data that you will help analyse but may also do further research on the samples to identify reasons for any changes seen.*

6. I understand that data relating to my participation in the study will be returned anonymously to my school to be used for educational purposes. *This means that although you will get to analyse data from your samples there is no way you will know which data relates to your samples and which to other participants*.

Complaints:

If you have any complaints about the way in which this study has been carried out please contact the Chair of the University of Exeter Medical School Research Ethics Committee Peta Foxall PhD, Chair, UEMS Research Ethics Committee: P.J.D.Foxall@exeter.ac.uk.

This project has been reviewed and approved by the University of Exeter Medical School Research Ethics committee UEMS REC REFERENCE NUMBER: 15/07/074)

Information Sheet - Version 4 (2/8/15)

BPA: Myth or Reality?

A research study investigating the effect of chemicals in plastic on gene activity and whether dietary interventions can reduce BPA levels in teenagers.

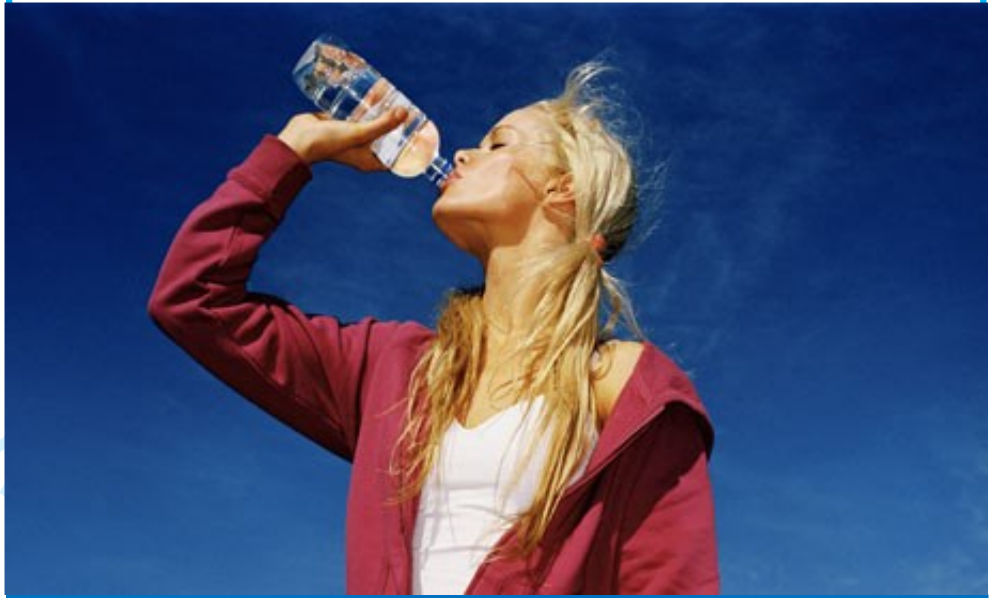

Involvement & Engagement

The aim of this year-long project is to involve teenagers in a research study that is relevant to them, by allowing them to help design a research project, analyse non-identifiable participant data and help to present and publish the outcomes.

Participation

Students will be asked to undertake a one week diet to reduce their intake of BPA, a chemical found in plastics. They will be asked to provide urine and blood samples before and after their diet.

For peer review only

For peer review only - http://bmjopen.bmj.com/site/about/guidelines.xhtml

What is BPA?

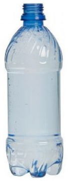

BPA (Bisphenol A) is a chemical used in the manufacture of plastics. Plastics containing BPA are found in a wide range of products including food and drink containers. BPA in these products can be ingested and there are concerns that high BPA levels in the blood could possibly affect human health. Research is therefore needed to understand its effects on the human body and how we can reduce its consumption by minor changes to our diet.

This project is being run as a student-involvement project to answer two specific questions:

1. **Can we see the effects of dietary BPA on our genes?**
2. **Can we effectively reduce BPA in our diet?**

In the past, small-scale experiments have shown that BPA levels in the human body can be reduced by rigid dietary interventions but these interventions would be difficult to implement in the "real world". In this study a one-week dietary intervention designed by teenagers will be used by them to determine whether BPA levels, and the activity of BPA-responsive genes can be effectively reduced in young people by avoiding food packaging that contains this chemical.

What will I need to do?

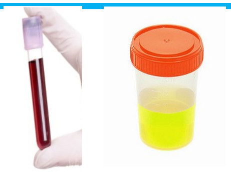

M

Day 1

- Provide a nurse with a 2.5ml blood sample and a urine sample.

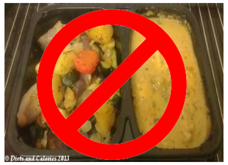

T

W

T

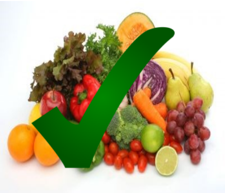

F

S

S

Day 2 - Day 8

- Follow a diet that you have helped to design .
- The diet will exclude sources of BPA as much as possible but will be nutritionally and calorifically similar to your usual diet.
- You will be asked to complete a food diary and answer a questionnaire about how easy it was to follow this diet.

Day 8

- Provide a nurse with a 2.5ml blood sample and a urine sample.

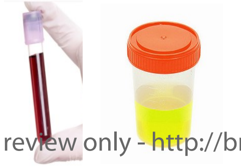

M

We recommend that you discuss the project with your family and involve them in planning what you eat and how you will prepare it.

What will happen to my samples and data?

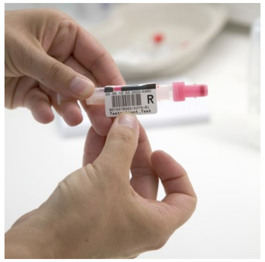

When you participate in the study you will be allocated with a numerical study ID. Your samples and data will be labelled with this number so that we can match your 'before' and 'after' diet samples with your food diary data. Once all data has been collated and coded it will be further anonymised by a person external to the project so that no data can be linked to any of the participants.

Urine samples from before and after the diet will be sent to the Royal Devon & Exeter NHS Foundation Trust for creatinine analysis and to the Rolvaltain laboratory for BPA analysis. RNA will be extracted from blood samples at the Royal Devon & Exeter Molecular Genetics Laboratory and the expression levels of two BPA-responsive genes will be measured in the samples taken before and after the diet. These anonymised RNA samples will be stored and used only by Professor Harries team for further research on the mechanisms behind our findings.

What are the benefits of taking part?

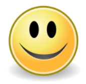

This project will help you to understand how you might be able to reduce BPA in your diet and your involvement in the design will give you an excellent insight into clinical research, community outreach and scientific practise. Your role as a participant is unlikely to have any direct health benefits.

Are there any risks in taking part?

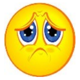

Blood samples will be taken by fully qualified and insured NHS personnel. Any potential discomfort or side-effects will be equivalent to that experienced giving a blood sample to your GP. All data will be fully anonymised before analysis. This means that you will not find out anything about your blood or urine samples. Following the diet may minimally increase the cost of your groceries for the week, but since fresh foods are usually less expensive than pre-packaged foods, we do not expect this to be an issue.

What will happen to the results of the research study?

You will be given the opportunity to help analyse anonymised data from this project and to help disseminate the outcomes of this research. It is hoped that the findings will be published in peer-reviewed journals and the wider media.

Who is organising this research?

The research is organised by Professors Lorna Harries & Tamara Galloway of the University of Exeter as part of their research program into BPA and part of the University's outreach program to involve schools in academic research.

For peer review only - http://bmjopen.bmj.com/site/about/guidelines.xhtml

Version 4 (2/8/2015)

BPA: Myth & Reality

STUDY ID

**CONSENT STATEMENTS**	**Please circle**
1. I confirm that I have read information sheet BPA PIS Version 4 and have discussed participation in this project with my family. I have had opportunity to consider the information, ask questions and have had these questions answered satisfactorily. *You should not give consent until you are happy that you understand what the study involves.*	**YES / NO**
2. I understand that my participation is voluntary and that I am free to withdraw at any time without giving any reason, without right to participate in the rest of the study being affected. *This means that even if you helped design this study you do not have to be a participant and you should not feel under any pressure to participate.*	**YES / NO**
3. I agree to participate in this study as a research subject. *This means that you agree to participate in a one-week diet and to provide two blood and urine samples.*	**YES / NO**
4. I understand that my anonymised blood and urine samples and linked anonymous questionnaire data will be sent to University of Exeter Medical School, Royal Devon & Hospital and my urine sample only will be sent to the Rolvaltain laboratory, a specialist BPA analysis company. *This means that laboratory staff will not know that samples belong to you, but dedicated staff at the University of Exeter, with training and experience in data protection, will be able to link your sample data to your questionnaire data.*	**YES / NO**
5. I understand that RNA (genetic material) will be extracted from my blood and will be stored anonymously. *This means that Professor Harries' team will use our RNA to provide data that you will help analyse but may also do further research on the samples to identify reasons for any changes seen.*	**YES / NO**
6. I understand that data relating to my participation in the study will be returned anonymously to my school to be used for educational purposes. *This means that although you will get to analyse data from your samples there is no way you will know which data relates to your samples and which to other participants*.	**YES / NO**

Name of Participant	Signature	Date
Name of Person Obtaining Consent	Signature	Date

I the above signed testify the participant is providing voluntary and fully informed consent to participate in this study. I am on the delegation log to obtain consent for this study and are trained in obtaining consent.

This project has been reviewed and approved by the University of Exeter Medical School Research Ethics Committee UEMS REC REFERENCE NUMBER: 15/07/074)

For peer review only

Version 4 (2/8/2015)

BPA: Myth & Reality

For peer review only

STROBE Statement—checklist of items that should be included in reports of observational studies

	Item No	Recommendation
**Title and abstract**	1	(*a*) Indicate the study's design with a commonly used term in the title or the abstract
		(*b*) Provide in the abstract an informative and balanced summary of what was done and what was found<br><br>**a) Page 1**<br>**b) Page 2**
**Introduction**		
Background/rationale	2	Explain the scientific background and rationale for the investigation being reported<br><br>**Page 6, line 82**
Objectives	3	State specific objectives, including any prespecified hypotheses<br><br>**Page 7, line 113**
**Methods**		
Study design	4	Present key elements of study design early in the paper<br><br>**Page 8, line 140**
Setting	5	Describe the setting, locations, and relevant dates, including periods of recruitment, exposure, follow-up, and data collection<br><br>**Page 8, line 152**
Participants	6	(*a*) *Cohort study*—Give the eligibility criteria, and the sources and methods of selection of participants. Describe methods of follow-up<br><br>**Page 7, line 123**<br><br>*Case-control study*—Give the eligibility criteria, and the sources and methods of case ascertainment and control selection. Give the rationale for the choice of cases and controls<br>*Cross-sectional study*—Give the eligibility criteria, and the sources and methods of selection of participants
		(*b*) *Cohort study*—For matched studies, give matching criteria and number of exposed and unexposed<br>*Case-control study*—For matched studies, give matching criteria and the number of controls per case
Variables	7	Clearly define all outcomes, exposures, predictors, potential confounders, and effect modifiers. Give diagnostic criteria, if applicable<br><br>Page 7, line 140, Page 8, line 152.

For peer review only

Data sources/ measurement	8*	For each variable of interest, give sources of data and details of methods of assessment (measurement). Describe comparability of assessment methods if there is more than one group<br><br>**Page 8, line 140, Page 8, line 152**
Bias	9	Describe any efforts to address potential sources of bias<br><br>**Page 8, line 154, page 9 line 165**
Study size	10	Explain how the study size was arrived at<br><br>**Page 7, line 127**
Quantitative variables	11	Explain how quantitative variables were handled in the analyses. If applicable, describe which groupings were chosen and why<br><br>**Page 9, line 157**
Statistical methods	12	(*a*) Describe all statistical methods, including those used to control for confounding<br><br>**Page 9, line 170**
		(*b*) Describe any methods used to examine subgroups and interactions<br><br>**Page 9, line 175**
		(*c*) Explain how missing data were addressed<br><br>**Page 9, line 164**
		(*d*) *Cohort study*—If applicable, explain how loss to follow-up was addressed<br><br>**Page 12, line 206**<br><br>*Case-control study*—If applicable, explain how matching of cases and controls was addressed<br>*Cross-sectional study*—If applicable, describe analytical methods taking account of sampling strategy
		(*e*) Describe any sensitivity analyses<br><br>**N/A**

Continued on next page

For peer review only - http://bmjopen.bmj.com/site/about/guidelines.xhtml

For peer review only

**Results**		
Participants	13*	(a) Report numbers of individuals at each stage of study—eg numbers potentially eligible, examined for eligibility, confirmed eligible, included in the study, completing follow-up, and analysed<br><br>**Table 1**
		(b) Give reasons for non-participation at each stage<br><br>**Page 12, line 206**
		(c) Consider use of a flow diagram<br><br>**N/A**
Descriptive data	14*	(a) Give characteristics of study participants (eg demographic, clinical, social) and information on exposures and potential confounders<br><br>**Table 1**
		(b) Indicate number of participants with missing data for each variable of interest<br><br>**Table 1**
		(c) *Cohort study*—Summarise follow-up time (eg, average and total amount)<br><br>**Page 9, line 172**
Outcome data	15*	*Cohort study*—Report numbers of outcome events or summary measures over time<br><br>**Table 1**
		Case-control study—Report numbers in each exposure category, or summary measures of exposure
		Cross-sectional study—Report numbers of outcome events or summary measures
Main results	16	(*a*) Give unadjusted estimates and, if applicable, confounder-adjusted estimates and their precision (eg, 95% confidence interval). Make clear which confounders were adjusted for and why they were included<br><br>**Page 12, line 213 to page 13 line 229**
		(*b*) Report category boundaries when continuous variables were categorized<br><br>**Page 9, line 176**
		(*c*) If relevant, consider translating estimates of relative risk into absolute risk for a meaningful

For peer review only - http://bmjopen.bmj.com/site/about/guidelines.xhtml

For peer review only

		time period<br><br>**N/A**
Other analyses	17	Report other analyses done—eg analyses of subgroups and interactions, and sensitivity analyses<br><br>**Page 13, line 234**
**Discussion**		
Key results	18	Summarise key results with reference to study objectives<br><br>**Page 15, line 276**
Limitations	19	Discuss limitations of the study, taking into account sources of potential bias or imprecision. Discuss both direction and magnitude of any potential bias<br><br>**Page 16, line 298**
Interpretation	20	Give a cautious overall interpretation of results considering objectives, limitations, multiplicity of analyses, results from similar studies, and other relevant evidence<br><br>**Page 17, line 334**
Generalisability	21	Discuss the generalisability (external validity) of the study results<br><br>**Page 18, line 344**
**Other information**		
Funding	22	Give the source of funding and the role of the funders for the present study and, if applicable, for the original study on which the present article is based<br><br>**Page 19, line 374**

*Give information separately for cases and controls in case-control studies and, if applicable, for exposed and unexposed groups in cohort and cross-sectional studies.

Note: An Explanation and Elaboration article discusses each checklist item and gives methodological background and published examples of transparent reporting. The STROBE checklist is best used in conjunction with this article (freely available on the Web sites of PLoS Medicine at http://www.plosmedicine.org/, Annals of Internal Medicine at http://www.annals.org/, and Epidemiology at http://www.epidem.com/). Information on the STROBE Initiative is available at www.strobe-statement.org.

For peer review only
